# Characteristics and outcomes of an international cohort of 600 000 hospitalized patients with COVID-19

**DOI:** 10.1093/ije/dyad012

**Published:** 2023-02-28

**Authors:** Christiana Kartsonaki, J Kenneth Baillie, Noelia García Barrio, Joaquín Baruch, Abigail Beane, Lucille Blumberg, Fernando Bozza, Tessa Broadley, Aidan Burrell, Gail Carson, Barbara Wanjiru Citarella, Andrew Dagens, Emmanuelle A Dankwa, Christl A Donnelly, Jake Dunning, Loubna Elotmani, Martina Escher, Nataly Farshait, Jean-Christophe Goffard, Bronner P Gonçalves, Matthew Hall, Madiha Hashmi, Benedict Sim Lim Heng, Antonia Ho, Waasila Jassat, Miguel Pedrera Jiménez, Cedric Laouenan, Samantha Lissauer, Ignacio Martin-Loeches, France Mentré, Laura Merson, Ben Morton, Daniel Munblit, Nikita A Nekliudov, Alistair D Nichol, Budha Charan Singh Oinam, David Ong, Prasan Kumar Panda, Michele Petrovic, Mark G Pritchard, Nagarajan Ramakrishnan, Grazielle Viana Ramos, Claire Roger, Oana Sandulescu, Malcolm G Semple, Pratima Sharma, Louise Sigfrid, Emily C Somers, Anca Streinu-Cercel, Fabio Taccone, Pavan Kumar Vecham, Bharath Kumar Tirupakuzhi Vijayaraghavan, Jia Wei, Evert-Jan Wils, Xin Ci Wong, Peter Horby, Amanda Rojek, Piero L Olliaro, ISARIC Clinical Characterisation Group, Ali Abbas, Sheryl Ann Abdukahil, Nurul Najmee Abdulkadir, Ryuzo Abe, Laurent Abel, Lara Absil, Subhash Acharya, Andrew Acker, Elisabeth Adam, Diana Adrião, Saleh Al Ageel, Shakeel Ahmed, Kate Ainscough, Eka Airlangga, Tharwat Aisa, Ali Ait Hssain, Younes Ait Tamlihat, Takako Akimoto, Ernita Akmal, Eman Al Qasim, Razi Alalqam, Angela Alberti, Tala Al-dabbous, Senthilkumar Alegesan, Cynthia Alegre, Marta Alessi, Beatrice Alex, Kévin Alexandre, Abdulrahman Al-Fares, Huda Alfoudri, Imran Ali, Adam Ali, Naseem Ali Shah, Kazali Enagnon Alidjnou, Jeffrey Aliudin, Qabas Alkhafajee, Clotilde Allavena, Nathalie Allou, Aneela Altaf, João Alves, Rita Alves, João Melo Alves, Maria Amaral, Nur Amira, Phoebe Ampaw, Roberto Andini, Claire Andréjak, Andrea Angheben, François Angoulvant, Séverine Ansart, Sivanesen Anthonidass, Massimo Antonelli, Carlos Alexandre Antunes de Brito, Ardiyan Apriyana, Yaseen Arabi, Irene Aragao, Francisco Arancibia, Carolline Araujo, Antonio Arcadipane, Patrick Archambault, Lukas Arenz, Jean-Benoît Arlet, Christel Arnold-Day, Lovkesh Arora, Rakesh Arora, Elise Artaud-Macari, Diptesh Aryal, Angel Asensio, Muhammad Ashraf, Namra Asif, Mohammad Asim, Jean Baptiste Assie, Amirul Asyraf, Anika Atique, A M Udara Lakshan Attanyake, Johann Auchabie, Hugues Aumaitre, Adrien Auvet, Laurène Azemar, Cecile Azoulay, Benjamin Bach, Delphine Bachelet, Claudine Badr, Nadia Baig, J Kevin Baird, Erica Bak, Agamemnon Bakakos, Nazreen Abu Bakar, Andriy Bal, Mohanaprasanth Balakrishnan, Valeria Balan, Firouzé Bani-Sadr, Renata Barbalho, Nicholas Yuri Barbosa, Wendy S Barclay, Saef Umar Barnett, Michaela Barnikel, Helena Barrasa, Audrey Barrelet, Cleide Barrigoto, Marie Bartoli, Mustehan Bashir, Romain Basmaci, Muhammad Fadhli Hassin Basri, Denise Battaglini, Jules Bauer, Diego Fernando Bautista Rincon, Denisse Bazan Dow, Alexandra Bedossa, Ker Hong Bee, Husna Begum, Sylvie Behilill, Albertus Beishuizen, Aleksandr Beljantsev, David Bellemare, Anna Beltrame, Beatriz Amorim Beltrão, Marine Beluze, Nicolas Benech, Lionel Eric Benjiman, Dehbia Benkerrou, Suzanne Bennett, Luís Bento, Jan-Erik Berdal, Delphine Bergeaud, Hazel Bergin, José Luis Bernal Sobrino, Giulia Bertoli, Lorenzo Bertolino, Simon Bessis, Sybille Bevilcaqua, Karine Bezulier, Amar Bhatt, Krishna Bhavsar, Claudia Bianco, Farah Nadiah Bidin, Moirangthem Bikram Singh, Felwa Bin Humaid, Mohd Nazlin Bin Kamarudin, François Bissuel, Patrick Biston, Laurent Bitker, Jonathan Bitton, Pablo Blanco-Schweizer, Catherine Blier, Frank Bloos, Mathieu Blot, Filomena Boccia, Laetitia Bodenes, Alice Bogaarts, Debby Bogaert, Anne-Hélène Boivin, Pierre-Adrien Bolze, François Bompart, Aurelius Bonfasius, Diogo Borges, Raphaël Borie, Hans Martin Bosse, Elisabeth Botelho-Nevers, Lila Bouadma, Olivier Bouchaud, Sabelline Bouchez, Dounia Bouhmani, Damien Bouhour, Kévin Bouiller, Laurence Bouillet, Camile Bouisse, Anne-Sophie Boureau, John Bourke, Maude Bouscambert, Aurore Bousquet, Jason Bouziotis, Bianca Boxma, Marielle Boyer-Besseyre, Maria Boylan, Axelle Braconnier, Cynthia Braga, Timo Brandenburger, Filipa Brás Monteiro, Luca Brazzi, Patrick Breen, Dorothy Breen, Patrick Breen, Kathy Brickell, Shaunagh Browne, Alex Browne, Nicolas Brozzi, Marjolein Brusse-Keizer, Nina Buchtele, Christian Buesaquillo, Polina Bugaeva, Marielle Buisson, Danilo Buonsenso, Erlina Burhan, Ingrid G Bustos, Denis Butnaru, André Cabie, Susana Cabral, Eder Caceres, Cyril Cadoz, Mia Callahan, Kate Calligy, Jose Andres Calvache, João Camões, Valentine Campana, Paul Campbell, Josie Campisi, Cecilia Canepa, Mireia Cantero, Pauline Caraux-Paz, Sheila Cárcel, Chiara Simona Cardellino, Sofia Cardoso, Filipe Cardoso, Filipa Cardoso, Nelson Cardoso, Simone Carelli, Nicolas Carlier, Thierry Carmoi, Gayle Carney, Inês Carqueja, Marie-Christine Carret, François Martin Carrier, Ida Carroll, Maire-Laure Casanova, Mariana Cascão, Siobhan Casey, José Casimiro, Bailey Cassandra, Silvia Castañeda, Nidyanara Castanheira, Guylaine Castor-Alexandre, Henry Castrillón, Ivo Castro, Ana Catarino, François-Xavier Catherine, Paolo Cattaneo, Roberta Cavalin, Giulio Giovanni Cavalli, Alexandros Cavayas, Adrian Ceccato, Minerva Cervantes-Gonzalez, Anissa Chair, Catherine Chakveatze, Adrienne Chan, Meera Chand, Christelle Chantalat Auger, Jean-Marc Chapplain, Julie Chas, Allegra Chatterjee, Mobin Chaudry, Jonathan Samuel Chávez Iñiguez, Anjellica Chen, Yih-Sharng Chen, Matthew Pellan Cheng, Antoine Cheret, Thibault Chiarabini, Julian Chica, Suresh Kumar Chidambaram, Leong Chin Tho, Catherine Chirouze, Davide Chiumello, Sung-Min Cho, Bernard Cholley, Marie-Charlotte Chopin, Ting Soo Chow, Yock Ping Chow, Hiu Jian Chua, Jonathan Chua, Jose Pedro Cidade, José Miguel Cisneros Herreros, Anna Ciullo, Jennifer Clarke, Emma Clarke, Rolando Claure-Del Granado, Sara Clohisey, Perren J Cobb, Cassidy Codan, Caitriona Cody, Alexandra Coelho, Megan Coles, Gwenhaël Colin, Michael Collins, Sebastiano Maria Colombo, Pamela Combs, Marie Connor, Anne Conrad, Sofía Contreras, Elaine Conway, Graham S Cooke, Mary Copland, Hugues Cordel, Amanda Corley, Sabine Cornelis, Alexander Daniel Cornet, Arianne Joy Corpuz, Andrea Cortegiani, Grégory Corvaisier, Emma Costigan, Camille Couffignal, Sandrine Couffin-Cadiergues, Roxane Courtois, Stéphanie Cousse, Rachel Cregan, Charles Crepy D'Orleans, Cosimo Cristella, Sabine Croonen, Gloria Crowl, Jonathan Crump, Claudina Cruz, Juan Luis Cruz Berm, Jaime Cruz Rojo, Marc Csete, Ailbhe Cullen, Matthew Cummings, Gerard Curley, Elodie Curlier, Colleen Curran, Paula Custodio, Ana da Silva Filipe, Charlene Da Silveira, Al-Awwab Dabaliz, Darren Dahly, Heidi Dalton, Jo Dalton, Seamus Daly, Nick Daneman, Corinne Daniel, Jorge Dantas, Frédérick D'Aragon, Menno de Jong, Gillian de Loughry, Diego de Mendoza, Etienne De Montmollin, Rafael Freitas de Oliveira França, Ana Isabel de Pinho Oliveira, Rosanna De Rosa, Cristina De Rose, Thushan de Silva, Peter de Vries, Jillian Deacon, David Dean, Alexa Debard, Marie-Pierre Debray, Nathalie DeCastro, William Dechert, Lauren Deconninck, Romain Decours, Eve Defous, Isabelle Delacroix, Eric Delaveuve, Karen Delavigne, Nathalie M Delfos, Ionna Deligiannis, Andrea Dell'Amore, Christelle Delmas, Pierre Delobel, Corine Delsing, Elisa Demonchy, Emmanuelle Denis, Dominique Deplanque, Pieter Depuydt, Mehul Desai, Diane Descamps, Mathilde Desvallées, Santi Dewayanti, Pathik Dhanger, Alpha Diallo, Sylvain Diamantis, André Dias, Juan Jose Diaz, Priscila Diaz, Rodrigo Diaz, Kévin Didier, Jean-Luc Diehl, Wim Dieperink, Jérôme Dimet, Vincent Dinot, Fara Diop, Alphonsine Diouf, Yael Dishon, Félix Djossou, Annemarie B Docherty, Helen Doherty, Arjen M Dondorp, Andy Dong, Maria Donnelly, Sean Donohue, Yoann Donohue, Chloe Donohue, Peter Doran, Céline Dorival, Eric D'Ortenzio, James Joshua Douglas, Renee Douma, Nathalie Dournon, Triona Downer, Joanne Downey, Mark Downing, Tom Drake, Aoife Driscoll, Murray Dryden, Murray Dryden, Claudio Duarte Fonseca, Vincent Dubee, François Dubos, Alexandre Ducancelle, Toni Duculan, Susanne Dudman, Abhijit Duggal, Paul Dunand, Mathilde Duplaix, Emanuele Durante-Mangoni, Lucian Durham, Bertrand Dussol, Juliette Duthoit, Xavier Duval, Anne Margarita Dyrhol-Riise, Sim Choon Ean, Marco Echeverria-Villalobos, Siobhan Egan, Carla Eira, Mohammed El Sanharawi, Subbarao Elapavaluru, Brigitte Elharrar, Jacobien Ellerbroek, Philippine Eloy, Tarek Elshazly, Iqbal Elyazar, Isabelle Enderle, Tomoyuki Endo, Chan Chee Eng, Ilka Engelmann, Vincent Enouf, Olivier Epaulard, Mariano Esperatti, Hélène Esperou, Marina Esposito-Farese, João Estevão, Manuel Etienne, Nadia Ettalhaoui, Anna Greti Everding, Mirjam Evers, Marc Fabre, Isabelle Fabre, Amna Faheem, Arabella Fahy, Cameron J Fairfield, Zul Fakar, Komal Fareed, Pedro Faria, Ahmed Farooq, Hanan Fateena, Arie Zainul Fatoni, Karine Faure, Raphaël Favory, Mohamed Fayed, Niamh Feely, Laura Feeney, Jorge Fernandes, Marília Andreia Fernandes, Susana Fernandes, François-Xavier Ferrand, Eglantine Ferrand Devouge, Joana Ferrão, Mário Ferraz, Sílvia Ferreira, Isabel Ferreira, Benigno Ferreira, Ricard Ferrer-Roca, Nicolas Ferriere, Céline Ficko, Claudia Figueiredo-Mello, William Finlayson, Juan Fiorda, Thomas Flament, Clara Flateau, Tom Fletcher, Letizia Lucia Florio, Deirdre Flynn, Claire Foley, Jean Foley, Victor Fomin, Tatiana Fonseca, Patricia Fontela, Simon Forsyth, Denise Foster, Giuseppe Foti, Erwan Fourn, Robert A Fowler, Marianne Fraher, Diego Franch-Llasat, John F Fraser, Christophe Fraser, Marcela Vieira Freire, Ana Freitas Ribeiro, Caren Friedrich, Ricardo Fritz, Stéphanie Fry, Nora Fuentes, Masahiro Fukuda, G Argin, Valérie Gaborieau, Rostane Gaci, Massimo Gagliardi, Jean-Charles Gagnard, Amandine Gagneux-Brunon, Sérgio Gaião, Linda Gail Skeie, Phil Gallagher, Carrol Gamble, Yasmin Gani, Arthur Garan, Rebekha Garcia, Julia Garcia-Diaz, Esteban Garcia-Gallo, Navya Garimella, Denis Garot, Valérie Garrait, Basanta Gauli, Nathalie Gault, Aisling Gavin, Anatoliy Gavrylov, Alexandre Gaymard, Johannes Gebauer, Eva Geraud, Louis Gerbaud Morlaes, Nuno Germano, praveen kumar ghisulal, Jade Ghosn, Marco Giani, Jess Gibson, Tristan Gigante, Morgane Gilg, Elaine Gilroy, Guillermo Giordano, Michelle Girvan, Valérie Gissot, Daniel Glikman, Petr Glybochko, Eric Gnall, Geraldine Goco, François Goehringer, Siri Goepel, Jin Yi Goh, Jonathan Golob, Rui Gomes, Kyle Gomez, Joan Gómez-Junyent, Marie Gominet, Alicia Gonzalez, Patricia Gordon, Isabelle Gorenne, Laure Goubert, Cécile Goujard, Tiphaine Goulenok, Margarite Grable, Jeronimo Graf, Edward Wilson Grandin, Pascal Granier, Giacomo Grasselli, Christopher A Green, Courtney Greene, William Greenhalf, Segolène Greffe, Domenico Luca Grieco, Matthew Griffee, Fiona Griffiths, Ioana Grigoras, Albert Groenendijk, Anja Grosse Lordemann, Heidi Gruner, Yusing Gu, Jérémie Guedj, Martin Guego, Dewi Guellec, Anne-Marie Guerguerian, Daniela Guerreiro, Romain Guery, Anne Guillaumot, Laurent Guilleminault, Maisa Guimarães de Castro, Thomas Guimard, Marieke Haalboom, Daniel Haber, Hannah Habraken, Ali Hachemi, Amy Hackmann, Nadir Hadri, Fakhir Haidri, Sheeba Hakak, Adam Hall, Sophie Halpin, Jawad Hameed, Ansley Hamer, Raph L Hamers, Rebecca Hamidfar, Terese Hammond, Lim Yuen Han, Rashan Haniffa, Kok Wei Hao, Hayley Hardwick, Ewen M Harrison, Janet Harrison, Samuel Bernard Ekow Harrison, Alan Hartman, Mohd Shahnaz Hasan, Junaid Hashmi, Muhammad Hayat, Ailbhe Hayes, Leanne Hays, Jan Heerman, Lars Heggelund, Ross Hendry, Martina Hennessy, Aquiles Henriquez-Trujillo, Maxime Hentzien, Jaime Hernandez-Montfort, Andrew Hershey, Liv Hesstvedt, Astarini Hidayah, Eibhilin Higgins, Dawn Higgins, Rupert Higgins, Rita Hinchion, Samuel Hinton, Hiroaki Hiraiwa, Haider Hirkani, Hikombo Hitoto, Yi Bin Ho, Alexandre Hoctin, Isabelle Hoffmann, Wei Han Hoh, Oscar Hoiting, Rebecca Holt, Jan Cato Holter, Juan Pablo Horcajada, Koji Hoshino, Ikram Houas, Catherine L Hough, Stuart Houltham, Jimmy Ming-Yang Hsu, Jean-Sébastien Hulot, Stella Huo, Abby Hurd, Iqbal Hussain, Samreen Ijaz, Hajnal-Gabriela Illes, Patrick Imbert, Mohammad Imran, Rana Imran Sikander, Aftab Imtiaz, Hugo Inácio, Carmen Infante Dominguez, Yun Sii Ing, Elias Iosifidis, Mariachiara Ippolito, Sarah Isgett, Tiago Isidoro, Nadiah Ismail, Margaux Isnard, Junji Itai, Daniel Ivulich, Danielle Jaafar, Salma Jaafoura, Julien Jabot, Clare Jackson, Nina Jamieson, Victoria Janes, Pierre Jaquet, Coline Jaud-Fischer, Stéphane Jaureguiberry, Jeffrey Javidfar, Denise Jaworsky, Florence Jego, Anilawati Mat Jelani, Synne Jenum, Ruth Jimbo-Sotomayor, Ong Yiaw Joe, Ruth N Jorge García, Cédric Joseph, Mark Joseph, Swosti Joshi, Mercé Jourdain, Philippe Jouvet, Hanna Jung, Anna Jung, Dafsah Juzar, Ouifiya Kafif, Florentia Kaguelidou, Neerusha Kaisbain, Thavamany Kaleesvran, Sabina Kali, Alina Kalicinska, Smaragdi Kalomoiri, Muhammad Aisar Ayadi Kamaluddin, Zul Amali Che Kamaruddin, Nadiah Kamarudin, Paul Kambiya, Kavita Kamineni, Darshana Hewa Kandamby, Chris Kandel, Kong Yeow Kang, Darakhshan Kanwal, Pratap Karpayah, Todd Karsies, Daisuke Kasugai, Anant Kataria, Kevin Katz, Aasmine Kaur, Christy Kay, Hannah Keane, Seán Keating, Pulak Kedia, Claire Kelly, Yvelynne Kelly, Andrea Kelly, Niamh Kelly, Aoife Kelly, Sadie Kelly, Maeve Kelsey, Ryan Kennedy, Kalynn Kennon, Maeve Kernan, Younes Kerroumi, Sharma Keshav, Imrana Khalid, Osama Khalid, Antoine Khalil, Coralie Khan, Irfan Khan, Quratul Ain Khan, Sushil Khanal, Abid Khatak, Amin Khawaja, Krish Kherajani, Michelle E Kho, Saye Khoo, Ryan Khoo, Denisa Khoo, Nasir Khoso, Khor How Kiat, Yuri Kida, Peter Kiiza, Beathe Kiland Granerud, Anders Benjamin Kildal, Jae Burm Kim, Antoine Kimmoun, Detlef Kindgen-Milles, Alexander King, Nobuya Kitamura, Paul Klenerman, Rob Klont, Gry Kloumann Bekken, Stephen R Knight, Robin Kobbe, Chamira Kodippily, Malte Kohns Vasconcelos, Sabin Koirala, Mamoru Komatsu, Caroline Kosgei, Arsène Kpangon, Karolina Krawczyk, Vinothini Krishnan, Sudhir Krishnan, Oksana Kruglova, Deepali Kumar, Ganesh Kumar, Mukesh Kumar, Dinesh Kuriakose, Ethan Kurtzman, Demetrios Kutsogiannis, Galyna Kutsyna, Konstantinos Kyriakoulis, Marie Lachatre, Marie Lacoste, John G Laffey, Marie Lagrange, Fabrice Laine, Olivier Lairez, Sanjay Lakhey, Antonio Lalueza, Marc Lambert, François Lamontagne, Marie Langelot-Richard, Vincent Langlois, Eka Yudha Lantang, Marina Lanza, Cédric Laouénan, Samira Laribi, Delphine Lariviere, Stéphane Lasry, Sakshi Lath, Naveed Latif, Odile Launay, Didier Laureillard, Yoan Lavie-Badie, Andy Law, Teresa Lawrence, Cassie Lawrence, Minh Le, Clément Le Bihan, Cyril Le Bris, Georges Le Falher, Lucie Le Fevre, Quentin Le Hingrat, Marion Le Maréchal, Soizic Le Mestre, Gwenaël Le Moal, Vincent Le Moing, Hervé Le Nagard, Paul Le Turnier, Ema Leal, Marta Leal Santos, Todd C Lee, James Lee, Jennifer Lee, Heng Gee Lee, Biing Horng Lee, Yi Lin Lee, Su Hwan Lee, Gary Leeming, Laurent Lefebvre, Bénédicte Lefebvre, Benjamin Lefevre, Sylvie LeGac, Jean-Daniel Lelievre, François Lellouche, Adrien Lemaignen, Véronique Lemee, Anthony Lemeur, Gretchen Lemmink, Ha Sha Lene, Jenny Lennon, Rafael León, Marc Leone, Michela Leone, Quentin Lepiller, François-Xavier Lescure, Olivier Lesens, Mathieu Lesouhaitier, Amy Lester-Grant, Bruno Levy, Yves Levy, Claire Levy-Marchal, Katarzyna Lewandowska, Erwan L'Her, Gianluigi Li Bassi, Janet Liang, Ali Liaquat, Geoffrey Liegeon, Wei Shen Lim, Kah Chuan Lim, Chantre Lima, Lim Lina, Bruno Lina, Andreas Lind, Maja Katherine Lingad, Guillaume Lingas, Sylvie Lion-Daolio, Keibun Liu, Marine Livrozet, Patricia Lizotte, Antonio Loforte, Navy Lolong, Leong Chee Loon, Diogo Lopes, Dalia Lopez-Colon, Jose W Lopez-Revilla, Anthony L Loschner, Paul Loubet, Bouchra Loufti, Guillame Louis, Silvia Lourenco, Lara Lovelace-Macon, Lee Lee Low, Marije Lowik, Jia Shyi Loy, Jean Christophe Lucet, Carlos Lumbreras Bermejo, Carlos M Luna, Olguta Lungu, Liem Luong, Nestor Luque, Dominique Luton, Nilar Lwin, Ruth Lyons, Olavi Maasikas, Oryane Mabiala, Moïse Machado, Gabriel Macheda, Hashmi Madiha, Guillermo Maestro de la Calle, Rafael Mahieu, Sophie Mahy, Ana Raquel Maia, Lars S Maier, Mylène Maillet, Thomas Maitre, Maximilian Malfertheiner, Nadia Malik, Paddy Mallon, Fernando Maltez, Denis Malvy, Victoria Manda, Laurent Mandelbrot, Frank Manetta, Julie Mankikian, Edmund Manning, Aldric Manuel, Ceila Maria Sant′Ana Malaque, Flávio Marino, Daniel Marino, Samuel Markowicz, Charbel Maroun Eid, Ana Marques, Catherine Marquis, Brian Marsh, Laura Marsh, Megan Marshal, John Marshall, Celina Turchi Martelli, Dori-Ann Martin, Emily Martin, Guillaume Martin-Blondel, Martin Martinot, Alejandro Martin-Quiros, João Martins, Ana Martins, Nuno Martins, Caroline Martins Rego, Gennaro Martucci, Olga Martynenko, Eva Miranda Marwali, Marsilla Marzukie, David Maslove, Sabina Mason, Sobia Masood, Basri Mat Nor, Moshe Matan, Meghena Mathew, Daniel Mathieu, Mathieu Mattei, Romans Matulevics, Laurence Maulin, Michael Maxwell, Javier Maynar, Thierry Mazzoni, Natalie Mc Evoy, Lisa Mc Sweeney, Colin McArthur, Colin McArthur, Anne McCarthy, Aine McCarthy, Colin McCloskey, Rachael McConnochie, Sherry McDermott, Sarah E McDonald, Aine McElroy, Samuel McElwee, Victoria McEneany, Allison McGeer, Chris McKay, Johnny McKeown, Kenneth A McLean, Paul McNally, Bairbre McNicholas, Elaine McPartlan, Edel Meaney, Cécile Mear-Passard, Maggie Mechlin, Maqsood Meher, Omar Mehkri, Ferruccio Mele, Luis Melo, Kashif Memon, Joao Joao Mendes, Ogechukwu Menkiti, Kusum Menon, Alexander J Mentzer, Emmanuelle Mercier, Noémie Mercier, Antoine Merckx, Mayka Mergeay-Fabre, Blake Mergler, António Mesquita, Roberta Meta, Osama Metwally, Agnès Meybeck, Dan Meyer, Alison M Meynert, Vanina Meysonnier, Amina Meziane, Mehdi Mezidi, Céline Michelanglei, Isabelle Michelet, Efstathia Mihelis, Vladislav Mihnovit, Hugo Miranda-Maldonado, Nor Arisah Misnan, Tahira Jamal Mohamed, Nik Nur Eliza Mohamed, Asma Moin, David Molina, Elena Molinos, Brenda Molloy, Mary Mone, Agostinho Monteiro, Claudia Montes, Giorgia Montrucchio, Shona C Moore, Sarah Moore, Lina Morales Cely, Lucia Moro, Catherine Motherway, Ana Motos, Hugo Mouquet, Clara Mouton Perrot, Julien Moyet, Caroline Mudara, Aisha Kalsoom Mufti, Ng Yong Muh, Dzawani Muhamad, Jimmy Mullaert, Fredrik Müller, Karl Erik Müller, Syed Muneeb, Nadeem Munir, Laveena Munshi, Aisling Murphy, Lorna Murphy, Aisling Murphy, Marlène Murris, Srinivas Murthy, Himed Musaab, Himasha Muvindi, Gugapriyaa Muyandy, Dimitra Melia Myrodia, Farah Nadia Mohd-Hanafiah, Dave Nagpal, Alex Nagrebetsky, Mangala Narasimhan, Nageswaran Narayanan, Rashid Nasim Khan, Alasdair Nazerali-Maitland, Nadège Neant, Holger Neb, Erni Nelwan, Raul Neto, Emily Neumann, Pauline Yeung Ng, Wing Yiu Ng, Anthony Nghi, Duc Nguyen, Orna Ni Choileain, Niamh Ni Leathlobhair, Prompak Nitayavardhana, Stephanie Nonas, Nurul Amani Mohd Noordin, Marion Noret, Nurul Faten Izzati Norharizam, Lisa Norman, Alessandra Notari, Mahdad Noursadeghi, Karolina Nowicka, Adam Nowinski, Saad Nseir, Jose I Nunez, Nurnaningsih Nurnaningsih, Dwi Utomo Nusantara, Elsa Nyamankolly, Fionnuala O Brien, Annmarie O Callaghan, Annmarie O'Callaghan, Giovanna Occhipinti, Derbrenn OConnor, Max O'Donnell, Tawnya Ogston, Takayuki Ogura, Tak-Hyuk Oh, Sophie O'Halloran, Katie O'Hearn, Shinichiro Ohshimo, Agnieszka Oldakowska, João Oliveira, Larissa Oliveira, Jee Yan Ong, Wilna Oosthuyzen, Anne Opavsky, Peter Openshaw, Saijad Orakzai, Claudia Milena Orozco-Chamorro, Jamel Ortoleva, Javier Osatnik, Linda O'Shea, Miriam O'Sullivan, Siti Zubaidah Othman, Nadia Ouamara, Rachida Ouissa, Eric Oziol, Maïder Pagadoy, Justine Pages, Mario Palacios, Amanda Palacios, Massimo Palmarini, Giovanna Panarello, Hem Paneru, Lai Hui Pang, Mauro Panigada, Nathalie Pansu, Aurélie Papadopoulos, Rachael Parke, Melissa Parker, Briseida Parra, Taha Pasha, Jérémie Pasquier, Bruno Pastene, Fabian Patauner, Drashti Patel, Mohan Dass Pathmanathan, Luís Patrão, Patricia Patricio, Juliette Patrier, Lisa Patterson, Rajyabardhan Pattnaik, Mical Paul, Christelle Paul, Jorge Paulos, William A Paxton, Jean-François Payen, Kalaiarasu Peariasamy, Giles J Peek, Florent Peelman, Nathan Peiffer-Smadja, Vincent Peigne, Mare Pejkovska, Paolo Pelosi, Ithan D Peltan, Rui Pereira, Daniel Perez, Luis Periel, Thomas Perpoint, Antonio Pesenti, Vincent Pestre, Lenka Petrou, Ventzislava Petrov-Sanchez, Frank Olav Pettersen, Gilles Peytavin, Scott Pharand, Michael Piagnerelli, Walter Picard, Olivier Picone, Maria de Piero, Carola Pierobon, Djura Piersma, Carlos Pimentel, Raquel Pinto, Catarina Pires, Isabelle Pironneau, Lionel Piroth, Ayodhia Pitaloka, Riinu Pius, Laurent Plantier, Hon Shen Png, Julien Poissy, Ryadh Pokeerbux, Maria Pokorska-Spiewak, Sergio Poli, Georgios Pollakis, Diane Ponscarme, Jolanta Popielska, Diego Bastos Porto, Andra-Maris Post, Douwe F Postma, Pedro Povoa, Diana Póvoas, Jeff Powis, Sofia Prapa, Sébastien Preau, Christian Prebensen, Jean-Charles Preiser, Anton Prinssen, Gamage Dona Dilanthi Priyadarshani, Lucia Proença, Sravya Pudota, Oriane Puéchal, Bambang Pujo Semedi, Mathew Pulicken, Gregory Purcell, Luisa Quesada, Vilmaris Quinones-Cardona, Víctor Quirós González, Else Quist-Paulsen, Mohammed Quraishi, Maia Rabaa, Christian Rabaud, Ebenezer Rabindrarajan, Aldo Rafael, Marie Rafiq, Gabrielle Ragazzo, Mutia Rahardjani, Rozanah Abd Rahman, Ahmad Kashfi Haji Ab Rahman, Arsalan Rahutullah, Fernando Rainieri, Giri Shan Rajahram, Pratheema Ramachandran, Ahmad Afiq Ramli, Blandine Rammaert, Asim Rana, Rajavardhan Rangappa, Ritika Ranjan, Christophe Rapp, Aasiyah Rashan, Thalha Rashan, Ghulam Rasheed, Menaldi Rasmin, Indrek Rätsep, Cornelius Rau, Tharmini Ravi, Ali Raza, Andre Real, Stanislas Rebaudet, Sarah Redl, Brenda Reeve, Attaur Rehman, Liadain Reid, Liadain Reid, Dag Henrik Reikvam, Renato Reis, Jordi Rello, Jonathan Remppis, Martine Remy, Hongru Ren, Hanna Renk, Anne-Sophie Resseguier, Matthieu Revest, Oleksa Rewa, Luis Felipe Reyes, Tiago Reyes, Maria Ines Ribeiro, Antonia Ricchiuto, David Richardson, Denise Richardson, Laurent Richier, Siti Nurul Atikah Ahmad Ridzuan, Jordi Riera, Ana L Rios, Asgar Rishu, Patrick Rispal, Karine Risso, Maria Angelica Rivera Nuñez, Nicholas Rizer, Chiara Robba, André Roberto, Stephanie Roberts, David L Robertson, Olivier Robineau, Ferran Roche-Campo, Paola Rodari, Simão Rodeia, Bernhard Roessler, Pierre-Marie Roger, Emmanuel Roilides, Juliette Romaru, Roberto Roncon-Albuquerque, Mélanie Roriz, Manuel Rosa-Calatrava, Michael Rose, Dorothea Rosenberger, Nurul Hidayah Mohammad Roslan, Andrea Rossanese, Matteo Rossetti, Bénédicte Rossignol, Patrick Rossignol, Stella Rousset, Carine Roy, Benoît Roze, Desy Rusmawatiningtyas, Clark D Russell, Maria Ryan, Maeve Ryan, Steffi Ryckaert, Aleksander Rygh Holten, Isabela Saba, Sairah Sadaf, Musharaf Sadat, Valla Sahraei, Maximilien Saint-Gilles, Pranya Sakiyalak, Nawal Salahuddin, Leonardo Salazar, Jodat Saleem, Gabriele Sales, Stéphane Sallaberry, Charlotte Salmon Gandonniere, Hélène Salvator, Olivier Sanchez, Angel Sanchez-Miralles, Vanessa Sancho-Shimizu, Gyan Sandhu, Zulfiqar Sandhu, Pierre-François Sandrine, Marlene Santos, Shirley Sarfo-Mensah, Bruno Sarmento Banheiro, Iam Claire E Sarmiento, Benjamine Sarton, Ankana Satya, Sree Satyapriya, Rumaisah Satyawati, Egle Saviciute, Parthena Savvidou, Yen Tsen Saw, Justin Schaffer, Tjard Schermer, Arnaud Scherpereel, Marion Schneider, Stephan Schroll, Michael Schwameis, Gary Schwartz, Brendan Scicluna, Janet T Scott, James Scott-Brown, Nicholas Sedillot, Tamara Seitz, Jaganathan Selvanayagam, Mageswari Selvarajoo, Caroline Semaille, Rasidah Bt Senian, Eric Senneville, Claudia Sepulveda, Filipa Sequeira, Tânia Sequeira, Ary Serpa Neto, Pablo Serrano Balazote, Ellen Shadowitz, Syamin Asyraf Shahidan, Mohammad Shamsah, Anuraj Shankar, Shaikh Sharjeel, Catherine A Shaw, Victoria Shaw, Ashraf Sheharyar, Rohan Shetty, Rajesh Mohan Shetty, Haixia Shi, Nisreen Shiban, Mohiuddin Shiekh, Nobuaki Shime, Hiroaki Shimizu, Keiki Shimizu, Sally Shrapnel, Pramesh Sundar Shrestha, Shubha Kalyan Shrestha, Hoi Ping Shum, Nassima Si Mohammed, Ng Yong Siang, Jeanne Sibiude, Atif Siddiqui, Piret Sillaots, Catarina Silva, Rogério Silva, Maria Joao Silva, Wai Ching Sin, Dario Sinatti, Punam Singh, Budha Charan Singh, Pompini Agustina Sitompul, Karisha Sivam, Vegard Skogen, Sue Smith, Benjamin Smood, Coilin Smyth, Michelle Smyth, Michelle Smyth, Morgane Snacken, Dominic So, Tze Vee Soh, Joshua Solomon, Tom Solomon, Agnès Sommet, Rima Song, Tae Song, Jack Song Chia, Michael Sonntagbauer, Azlan Mat Soom, Alberto Sotto, Edouard Soum, Marta Sousa, Ana Chora Sousa, Maria Sousa Uva, Vicente Souza-Dantas, Alexandra Sperry, Elisabetta Spinuzza, B P Sanka Ruwan Sri Darshana, Shiranee Sriskandan, Sarah Stabler, Thomas Staudinger, Stephanie-Susanne Stecher, Trude Steinsvik, Ymkje Stienstra, Birgitte Stiksrud, Eva Stolz, Amy Stone, Adrian Streinu-Cercel, David Stuart, Ami Stuart, Decy Subekti, Gabriel Suen, Jacky Y Suen, Asfia Sultana, Charlotte Summers, Dubravka Supic, Deepashankari Suppiah, Magdalena Surovcová, Suwarti Suwarti, Andrey Svistunov, Sarah Syahrin, Konstantinos Syrigos, Jaques Sztajnbok, Konstanty Szuldrzynski, Shirin Tabrizi, Lysa Tagherset, Shahdattul Mawarni Taib, Ewa Talarek, Sara Taleb, Jelmer Talsma, Renaud Tamisier, Maria Lawrensia Tampubolon, Kim Keat Tan, Yan Chyi Tan, Taku Tanaka, Hiroyuki Tanaka, Hayato Taniguchi, Huda Taqdees, Arshad Taqi, Coralie Tardivon, Pierre Tattevin, M Azhari Taufik, Hassan Tawfik, Richard S Tedder, Tze Yuan Tee, João Teixeira, Sofia Tejada, Marie-Capucine Tellier, Sze Kye Teoh, Vanessa Teotonio, François Téoulé, Pleun Terpstra, Olivier Terrier, Nicolas Terzi, Hubert Tessier-Grenier, Adrian Tey, Alif Adlan Mohd Thabit, Anand Thakur, Zhang Duan Tham, Suvintheran Thangavelu, Vincent Thibault, Simon-Djamel Thiberville, Benoît Thill, Jananee Thirumanickam, Shaun Thompson, Emma C Thomson, David Thomson, Surain Raaj Thanga Thurai, Ryan S Thwaites, Paul Tierney, Vadim Tieroshyn, Peter S Timashev, Jean-François Timsit, Noémie Tissot, Jordan Zhien Yang Toh, Maria Toki, Kristian Tonby, Sia Loong Tonnii, Margarida Torres, Antoni Torres, Rosario Maria Torres Santos-Olmo, Hernando Torres-Zevallos, Michael Towers, Tony Trapani, Théo Treoux, Cécile Tromeur, Ioannis Trontzas, Tiffany Trouillon, Jeanne Truong, Christelle Tual, Sarah Tubiana, Helen Tuite, Jean-Marie Turmel, Lance C W Turtle, Anders Tveita, Pawel Twardowski, Makoto Uchiyama, P G Ishara Udayanga, Andrew Udy, Roman Ullrich, Alberto Uribe, Asad Usman, Timothy M Uyeki, Cristinava Vajdovics, Piero Valentini, Luís Val-Flores, Amélie Valran, Stijn Van de Velde, Marcel van den Berge, Machteld Van der Feltz, Job van der Palen, Paul van der Valk, Nicky Van Der Vekens, Peter Van der Voort, Sylvie Van Der Werf, Laura van Gulik, Jarne Van Hattem, Carolien van Netten, Frank van Someren Greve, Ilonka van Veen, Hugo Van Willigen, Noémie Vanel, Henk Vanoverschelde, Pooja Varghese, Michael Varrone, Shoban Raj Vasudayan, Charline Vauchy, Shaminee Veeran, Aurélie Veislinger, Sebastian Vencken, Sara Ventura, Annelies Verbon, James Vickers, José Ernesto Vidal, César Vieira, Deepak Vijayan, Joy Ann Villanueva, Judit Villar, Pierre-Marc Villeneuve, Andrea Villoldo, Gayatri Vishwanathan, Benoit Visseaux, Hannah Visser, Chiara Vitiello, Harald Vonkeman, Fanny Vuotto, Suhaila Abdul Wahab, Noor Hidayu Wahab, Nadirah Abdul Wahid, Marina Wainstein, Wan Fadzlina Wan Muhd Shukeri, Chih-Hsien Wang, Steve Webb, Katharina Weil, Tan Pei Wen, Sanne Wesselius, T Eoin West, Murray Wham, Bryan Whelan, Nicole White, Paul Henri Wicky, Aurélie Wiedemann, Surya Otto Wijaya, Keith Wille, Suzette Willems, Virginie Williams, Calvin Wong, Yew Sing Wong, Teck Fung Wong, Natalie Wright, Gan Ee Xian, Lim Saio Xian, Kuan Pei Xuan, Ioannis Xynogalas, Siti Rohani Binti Mohd Yakop, Masaki Yamazaki, Yazdan Yazdanpanah, Nicholas Yee Liang Hing, Cécile Yelnik, Chian Hui Yeoh, Stephanie Yerkovich, Toshiki Yokoyama, Hodane Yonis, Obada Yousif, Saptadi Yuliarto, Akram Zaaqoq, Marion Zabbe, Kai Zacharowski, Masliza Zahid, Maram Zahran, Nor Zaila Binti Zaidan, Maria Zambon, Miguel Zambrano, Alberto Zanella, Konrad Zawadka, Nurul Zaynah, Hiba Zayyad, Alexander Zoufaly, David Zucman

**Affiliations:** Medical Research Council (MRC) Population Health Research Unit, Clinical Trials Service Unit and Epidemiological Studies Unit, Nuffield Department of Population Health, University of Oxford, Oxford, UK; Roslin Institute, University of Edinburgh, Edinburgh, UK; Intensive Care Unit, Royal Infirmary of Edinburgh, Edinburgh, UK; Hospital 12 de Octubre, Madrid, Spain; International Severe Acute Respiratory and emerging Infection Consortium (ISARIC) Global Support Centre, Pandemic Sciences Institute, Nuffield Department of Medicine, University of Oxford, Oxford, UK; Critical Care Asia, Bangkok, Thailand; National Institute for Communicable Diseases, Johannesburg, South Africa; National Institute of Infectious Disease Evandro Chagas, Oswaldo Cruz Foundation (INI-FIOCRUZ), Ministry of Health, and D'Or Institute of Research and Education (IDOR), Rio de Janeiro, São Paulo, Brazil; Monash University, Clayton, Melbourne, Australia; Monash University, Clayton, Melbourne, Australia; International Severe Acute Respiratory and emerging Infection Consortium (ISARIC) Global Support Centre, Pandemic Sciences Institute, Nuffield Department of Medicine, University of Oxford, Oxford, UK; International Severe Acute Respiratory and emerging Infection Consortium (ISARIC) Global Support Centre, Pandemic Sciences Institute, Nuffield Department of Medicine, University of Oxford, Oxford, UK; International Severe Acute Respiratory and emerging Infection Consortium (ISARIC) Global Support Centre, Pandemic Sciences Institute, Nuffield Department of Medicine, University of Oxford, Oxford, UK; Department of Statistics, University of Oxford, Oxford, UK; Department of Statistics, University of Oxford, Oxford, UK; MRC Centre for Global Infectious Disease Analysis, Abdul Latif Jameel Institute for Disease and Emergency Analytics and Department of Infectious Disease Epidemiology, Imperial College London, London, UK; International Severe Acute Respiratory and emerging Infection Consortium (ISARIC) Global Support Centre, Pandemic Sciences Institute, Nuffield Department of Medicine, University of Oxford, Oxford, UK; CHU Caremeau, Nîmes, France; International Severe Acute Respiratory and emerging Infection Consortium (ISARIC) Global Support Centre, Pandemic Sciences Institute, Nuffield Department of Medicine, University of Oxford, Oxford, UK; Humber River Hospital, Toronto, Canada; Cliniques Universitaires de Bruxelles (CUB) Hopital Erasme, Anderlecht, Belgium; International Severe Acute Respiratory and emerging Infection Consortium (ISARIC) Global Support Centre, Pandemic Sciences Institute, Nuffield Department of Medicine, University of Oxford, Oxford, UK; Big Data Institute, Nuffield Department of Medicine, University of Oxford, Oxford, UK; Critical Care Asia and Ziauddin University, Karachi, Pakistan; Hospital Sungai Buloh, Ministry of Health, Sungai Buloh, Malaysia; Medical Research Council-University of Glasgow Centre for Virus Research, Glasgow, UK Department of Infectious Diseases, Queen Elizabeth University Hospital, Glasgow, UK; National Institute for Communicable Diseases, Johannesburg, South Africa; Hospital 12 de Octubre, Madrid, Spain; Un iversité de Paris, France, Infection, Antimicrobials, Modelling, Evolution (IAME), INSERM, Paris, France; Malawi-Liverpool Wellcome Trust, Blantyre, Malawi; St James's Hospital, Dublin, Ireland; Un iversité de Paris, France, Infection, Antimicrobials, Modelling, Evolution (IAME), INSERM, Paris, France; International Severe Acute Respiratory and emerging Infection Consortium (ISARIC) Global Support Centre, Pandemic Sciences Institute, Nuffield Department of Medicine, University of Oxford, Oxford, UK; Infectious Diseases Data Observatory, Centre for Tropical Medicine and Global Health, University of Oxford, Oxford, UK; Liverpool School of Tropical Medicine, Liverpool, UK; Department of Paediatrics and Paediatric Infectious Diseases, Institute of Child’s Health, Sechenov First Moscow State Medical University (Sechenov University), Moscow, Russia; Inflammation, Repair and Development Section, National Heart and Lung Institute, Faculty of Medicine, Imperial College London, London, UK; Sechenov University, Moscow, Russia; Irish Critical Care Critical Clinical Trials Network, Dublin, Ireland; All India Institute of Medical Sciences (AIIMS), Rishikesh, India; Franciscus Gasthuis & Vlietland, Rotterdam, Netherlands; All India Institute of Medical Sciences (AIIMS), Rishikesh, India; Humber River Hospital, Toronto, Canada; International Severe Acute Respiratory and emerging Infection Consortium (ISARIC) Global Support Centre, Pandemic Sciences Institute, Nuffield Department of Medicine, University of Oxford, Oxford, UK; Apollo Hospitals Chennai, Chennai, India; National Institute of Infectious Disease Evandro Chagas, Oswaldo Cruz Foundation (INI-FIOCRUZ), Ministry of Health, and D'Or Institute of Research and Education (IDOR), Rio de Janeiro, São Paulo, Brazil; CHU Caremeau, Nîmes, France; Carol Davila University of Medicine and Pharmacy, Bucharest, Romania; National Institute for Infectious Diseases ‘Prof. Dr. Matei Bals’, Bucharest, Romania; Institute of Infection, Veterinary and Ecological Sciences, Faculty of Health and Life Sciences, University of Liverpool, Liverpool, UK; UK Respiratory Medicine, Alder Hey Children’s NHS Foundation Trust, Liverpool, UK; University of Michigan Schools of Medicine & Public Health, Ann Arbor, Michigan, USA; International Severe Acute Respiratory and emerging Infection Consortium (ISARIC) Global Support Centre, Pandemic Sciences Institute, Nuffield Department of Medicine, University of Oxford, Oxford, UK; University of Michigan Schools of Medicine & Public Health, Ann Arbor, Michigan, USA; Carol Davila University of Medicine and Pharmacy, Bucharest, Romania; Cliniques Universitaires de Bruxelles (CUB) Hopital Erasme, Anderlecht, Belgium; Apollo Hospitals Chennai, Chennai, India; Department of Critical Care Medicine, Apollo Hospitals, Chennai, India; The George Institute for Global Health, New Delhi, India; Big Data Institute, Nuffield Department of Medicine, University of Oxford, Oxford, UK; Franciscus Gasthuis & Vlietland, Rotterdam, Netherlands; National Institutes of Health (NIH), Ministry of Health, Shah Alam, Malaysia; International Severe Acute Respiratory and emerging Infection Consortium (ISARIC) Global Support Centre, Pandemic Sciences Institute, Nuffield Department of Medicine, University of Oxford, Oxford, UK; International Severe Acute Respiratory and emerging Infection Consortium (ISARIC) Global Support Centre, Pandemic Sciences Institute, Nuffield Department of Medicine, University of Oxford, Oxford, UK; Royal Melbourne Hospital, Melbourne, Australia; Centre for Integrated Critical Care, University of Melbourne, Melbourne, Australia; International Severe Acute Respiratory and emerging Infection Consortium (ISARIC) Global Support Centre, Pandemic Sciences Institute, Nuffield Department of Medicine, University of Oxford, Oxford, UK

**Keywords:** COVID-19, SARS-CoV-2, cohort study, risk of death, co-morbidities, symptoms, treatments

## Abstract

**Background:**

We describe demographic features, treatments and clinical outcomes in the International Severe Acute Respiratory and emerging Infection Consortium (ISARIC) COVID-19 cohort, one of the world's largest international, standardized data sets concerning hospitalized patients.

**Methods:**

The data set analysed includes COVID-19 patients hospitalized between January 2020 and January 2022 in 52 countries. We investigated how symptoms on admission, co-morbidities, risk factors and treatments varied by age, sex and other characteristics. We used Cox regression models to investigate associations between demographics, symptoms, co-morbidities and other factors with risk of death, admission to an intensive care unit (ICU) and invasive mechanical ventilation (IMV).

**Results:**

Data were available for 689 572 patients with laboratory-confirmed (91.1%) or clinically diagnosed (8.9%) SARS-CoV-2 infection from 52 countries. Age [adjusted hazard ratio per 10 years 1.49 (95% CI 1.48, 1.49)] and male sex [1.23 (1.21, 1.24)] were associated with a higher risk of death. Rates of admission to an ICU and use of IMV increased with age up to age 60 years then dropped. Symptoms, co-morbidities and treatments varied by age and had varied associations with clinical outcomes. The case-fatality ratio varied by country partly due to differences in the clinical characteristics of recruited patients and was on average 21.5%.

**Conclusions:**

Age was the strongest determinant of risk of death, with a ∼30-fold difference between the oldest and youngest groups; each of the co-morbidities included was associated with up to an almost 2-fold increase in risk. Smoking and obesity were also associated with a higher risk of death. The size of our international database and the standardized data collection method make this study a comprehensive international description of COVID-19 clinical features. Our findings may inform strategies that involve prioritization of patients hospitalized with COVID-19 who have a higher risk of death.

Key Messages

- Several studies have investigated the risks of severe illness and death due to infection with SARS-CoV-2, providing estimates of the case-fatality ratio in different settings and risk factors for these outcomes, but these tended to be national studies conducted over a short time period.
- We show how clinical presentation and risks of death and admission to an intensive care unit vary with patient characteristics based on a very large number of patient records from 52 countries, collected using standardized data collection tools.
- Age was the strongest determinant of risk; pre-existing co-morbidities and male sex were also associated with higher risk of death.
- Smoking and obesity are modifiable risk factors that are associated with a higher risk of death.
- Our findings may inform strategies that involve prioritization of patients, globally, who have a higher risk of adverse outcomes if hospitalized with COVID-19, as well as prevention strategies.

## Introduction

To respond to COVID-19, policymakers and clinicians need robust data to drive the decision-making processes that save or cost lives. Observational cohort data describing clinical characteristics and the likelihood of severe outcomes can guide health policy development, produce research hypotheses for clinical trials and improve clinical guidelines for patient care.^1^ Across the world, multiple cohort studies have described the clinical impact of the COVID-19 pandemic^2–8^ but heterogeneity in study features makes combining and comparing the findings challenging.

The Clinical Characterisation Protocol (CCP) developed by the International Severe Acute Respiratory and emerging Infection Consortium (ISARIC) and the World Health Organization (WHO)^9^ has helped researchers across the world to collect and analyse clinical data.^10^ Thanks to this international co-operation, it has been possible to produce a truly global cohort study using standardized clinical data. The growing data set has been analysed regularly, with results shared via reports,^11^ and the data set has been used to address specific research questions.^12^^,^^13^

We present data from an international cohort of almost 700 000 patients from 1380 sites across 52 countries. We summarize the demographic features and clinical presentation of hospitalized patients with COVID-19 in low-, middle- and high-resource settings. We characterize the variability in clinical features in these patients and explore the risk factors associated with mortality, and the need for intensive care and mechanical ventilation, on a global scale. We aimed to report a general description of this international data set 2 years after the beginning of the pandemic, to estimate the frequency of co-morbidities, risk factors, symptoms and use of different treatments, and to estimate the risk of severe clinical outcomes and the associations of various factors with risk of these outcomes.

## Methods

### Study

We used international prospective observational data of clinical features and outcomes of patients admitted to hospital with COVID-19. The ISARIC/WHO CCP, incorporating Short PeRiod IncideNce sTudy of Severe Acute Respiratory Infection (SPRINT-SARI),^14^ is a standardized protocol for investigations of (re-)emerging pathogens of public health interest. All data were stored at a central repository at the University of Oxford, England. Patients with clinically suspected or laboratory-confirmed COVID-19 infection were enrolled in the study. Participating sites used the ISARIC case report form^15^ to enter data onto a Research Electronic Data Capture (REDCap, version 8.11.11, Vanderbilt University, Nashville, TN) database or used local databases (Supplementary Methods, available as Supplementary data at *IJE* online) before uploading to the central data repository. Centrally collated data were wrangled and mapped to the structure and controlled terminologies of Study Data Tabulation Model (version 1.7, Clinical Data Interchange Standards Consortium, Austin, TX) using Trifacta^®^ software. The data collection, aggregation, curation and harmonization process has been previously described.^16^ The first patient was enrolled on 30 January 2020. This analysis includes all patients whose data were entered up to 5 January 2022.

### Participants

We included hospitalized patients of any age with clinically or laboratory-diagnosed COVID-19. This analysis included patients admitted to hospital in any of the countries which contributed data. It also includes a small subset of asymptomatic patients who were admitted to the hospital purely for isolation (<1%).

### Statistical analysis

**Figure 1:**
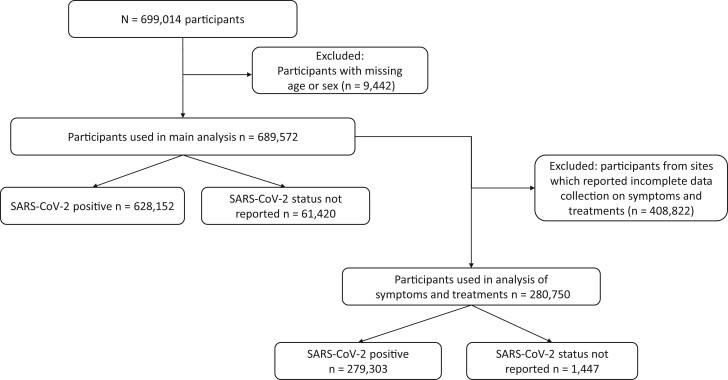
Numbers of participants. 6321 (0.92%) of the participants included in the main analysis were admitted to hospital for isolation

We excluded patients with missing age or sex from all analyses (Figure 1). Additionally, we excluded from analyses of symptoms or treatments sites that did not record information on these two sets of variables. We calculated medians and interquartile ranges (IQRs) for continuous variables and proportions for categorical variables. We calculated proportions of patients who met each of the WHO, Centers for Disease Control and Prevention (CDC) of the USA, European Centre for Disease Prevention and Control (ECDC) and Public Health England (PHE) symptom-based case definitions (Supplementary Methods, available as Supplementary data at *IJE* online). We calculated case-fatality ratios (CFRs) overall, by country and by month, using the method suggested by Ghani *et al.*^17^ We calculated inverse-variance weighted CFRs by country.

Patients were followed up from hospital admission to death, discharge or censoring, whichever occurred first. Cox proportional hazards models were used to assess the association of demographic variables, co-morbidities and symptoms at admission (unless symptom onset was after admission) with risk of death, admission to an intensive care unit (ICU) or a high-dependency unit (hereafter referred to collectively as ICU) or use of invasive mechanical ventilation (IMV). Individuals were censored if they were lost to follow-up (e.g. transferred to another facility) or remained in hospital on 6 January 2022. Time from symptom onset to the time of death or censoring, whichever occurred earlier, was used as the timescale. Patients were considered at risk from the time of symptom onset or admission, whichever occurred later. For all outcomes, censoring times of discharged patients were modified and set to be equal to the maximum time to censoring/event (to account for informative censoring). For associations with admission to an ICU, the timescale was from symptom onset to the earliest of admission to an ICU, death, discharge and censoring. The event was admission to an ICU. For associations with receipt of IMV, the timescale was from symptom onset to the earliest of IMV, death, discharge and censoring. The event was IMV. Models were adjusted for age and sex, and stratified by country. We grouped countries with <50 individuals into a single category. Hazard ratios (HRs) and 95% CIs were estimated. We assessed the proportional hazards assumption using scaled Schoenfeld residuals. For explanatory variables with multiple categories (such as age groups), we used quasi-standard errors^18^ to facilitate comparisons between any two groups.

We repeated the main analyses for patients with laboratory-confirmed SARS-CoV-2 only, as a sensitivity analysis. For associations of age and sex with risk of death, we also estimated HRs within each country and calculated an overall HR using inverse-variance weighting. For age, sex and co-morbidities, we also estimated associations within the first (2020) and second year (2021) of the pandemic.

In total, 9442 individuals had missing age or sex (Figure 1) and were excluded from the analysis. Missingness for other variables among included patients varied from 2% to 93%. For analyses on the prevalence of co-morbidities, risk factors, symptoms or treatments, ‘missing’ is shown as a separate category in all tables and figures. For analyses on associations with outcomes, a complete case approach was used.

Analysis was performed using R version 4.1.1 and packages *survival*, *ggplot2*, *qvcalc* and *finalfit*.

### Patient and public involvement

This was an urgent public health research study in response to a Public Health Emergency of International Concern. Patients or the public were not involved in the design, conduct or reporting of this rapid response research. ISARIC, ISARIC4C, the National Institute of Communicable Diseases South Africa and other collaborators have public facing websites (isaric.org; isaric4c.net; nicd.ac.za) and social media accounts to disseminate findings. The contributing individuals and institutions engage with print and internet press, television, radio, news and documentary programme makers to share evidence with the public and invite feedback.

## Results

### Participants’ characteristics

**Figure 2:**
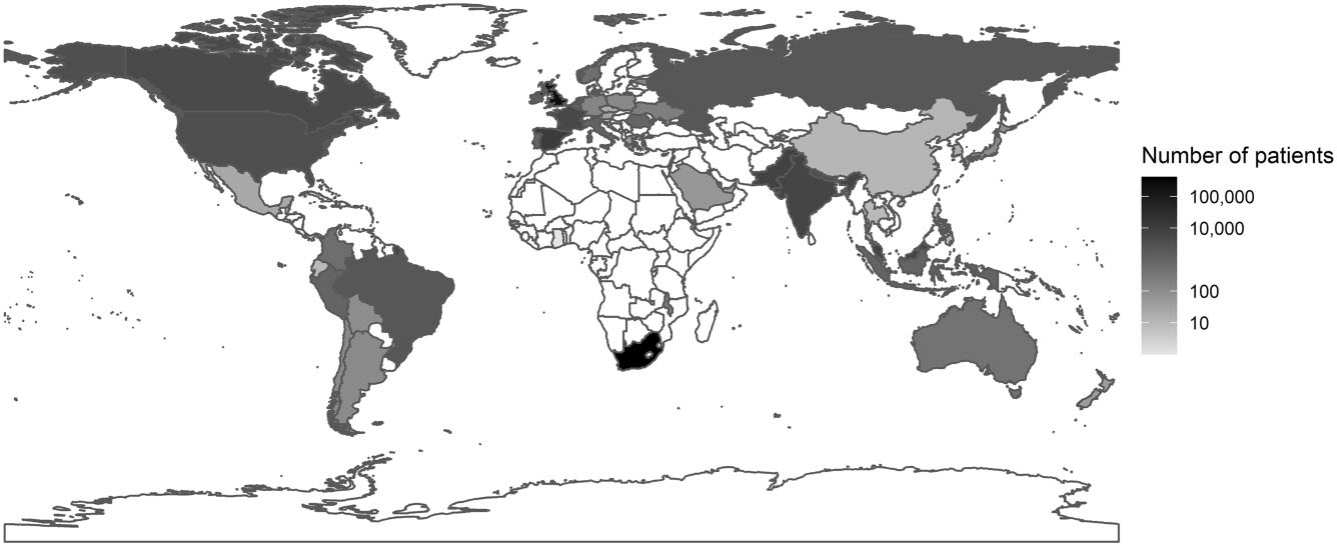
Numbers of patients by country

In total, 689 572 patients (Figure 1) were included from 1380 sites in 52 countries (Figure 2 and Supplementary Figure S1, available as Supplementary data at *IJE* online). Overall, 91.1% of the participants included in the primary analysis had a positive SARS-CoV-2 PCR test (Table 1); 49.4% were male and the median age was 58 years (range 0 to 119, IQR 30). The median time from the latest of symptom onset or admission to discharge, death or date last known to be alive was 6 (IQR 10) days. Among 679 194 individuals with a known ICU admission status, 15.9% were admitted to an ICU, a third of whom were admitted directly (on the day of hospitalization). Oxygen saturation on presentation to hospital was reported for 34.8% of patients with a median SpO2 of 96% (IQR 4%).

**Table 1:** Participants’ characteristics

	*N* recorded (%)		Female	Male	Total
Total *N*			349 267	340 305	689 572
Age	689 572 (100.0)	Median (IQR)	57.0 (39.0 to 72.0)	58.1 (45.0 to 72.0)	58.0 (42.0 to 72.0)
		0–9	6863 (2.0)	8624 (2.5)	15 487 (2.2)
		10–19	9023 (2.6)	6594 (1.9)	15 617 (2.3)
		20–29	27 684 (7.9)	15 233 (4.5)	42 917 (6.2)
		30–39	44 367 (12.7)	32 124 (9.4)	76 491 (11.1)
		40–49	44 562 (12.8)	48 309 (14.2)	92 871 (13.5)
		50–59	60 856 (17.4)	66 106 (19.4)	126 962 (18.4)
		60–69	56 001 (16.0)	63 357 (18.6)	119 358 (17.3)
		70–79	48 288 (13.8)	54 735 (16.1)	103 023 (14.9)
		80–89	38 383 (11.0)	36 251 (10.7)	74 634 (10.8)
		90+	13 240 (3.8)	8972 (2.6)	22 212 (3.2)
Region	689 572 (100.0)	East Asia and Pacific	2551 (0.7)	5565 (1.6)	8116 (1.2)
		Europe and Central Asia	108 670 (31.1)	133 306 (39.2)	241 976 (35.1)
		Latin America and Caribbean	1451 (0.4)	2618 (0.8)	4069 (0.6)
		Middle East and North Africa	400 (0.1)	608 (0.2)	1008 (0.1)
		North America	3346 (1.0)	4733 (1.4)	8079 (1.2)
		South Asia	5882 (1.7)	11 226 (3.3)	17 108 (2.5)
		Sub-Saharan Africa	226 967 (65.0)	182 249 (53.6)	409 216 (59.3)
Onset to admission (days)	619 812 (89.9)	Median (IQR)	0.0 (0.0 to 3.0)	0.0 (0.0 to 5.0)	0.0 (0.0 to 4.0)
Length of hospital stay (days)	689 572 (100.0)	Median (IQR)	6.0 (2.0 to 11.0)	6.0 (3.0 to 12.0)	6.0 (2.0 to 12.0)
Body mass index (kg/m²)	18 582 (2.7)	Median (IQR)	27.7 (23.5 to 32.5)	27.3 (24.2 to 30.9)	27.5 (24.0 to 31.2)
Heart rate on admission (b.p.m.)	229 125 (33.2)	Median (IQR)	90.0 (79.0 to 103.0)	90.0 (78.0 to 103.0)	90.0 (79.0 to 103.0)
Systolic blood pressure on admission (mmHg)	236 114 (34.2)	Median (IQR)	128.0 (114.0 to 144.0)	129.0 (116.0 to 143.0)	129.0 (115.0 to 144.0)
Diastolic blood pressure on admission (mmHg)	236 097 (34.2)	Median (IQR)	73.0 (65.0 to 82.0)	75.0 (67.0 to 84.0)	74.0 (66.0 to 83.0)
Temperature on admission (degrees C)	238 084 (34.5)	Median (IQR)	37.0 (36.5 to 37.7)	37.0 (36.5 to 37.8)	37.0 (36.5 to 37.8)
Oxygen saturation on admission (%)	240 068 (34.8)	Median (IQR)	96.0 (93.0 to 98.0)	95.0 (92.0 to 97.0)	96.0 (93.0 to 97.0)
− On oxygen therapy	78 961 (32.9%*)	Median (IQR)	95.0 (92.0 to 97.0)	95.0 (92.0 to 97.0)	95.0 (92.0 to 97.0)
− In room air	150 585 (62.7%*)	Median (IQR)	96.0 (94.0 to 98.0)	96.0 (93.0 to 97.0)	96.0 (93.0 to 98.0)
− Unknown oxygenation status	10 689 (4.5%*)	Median (IQR)	96.0 (93.0 to 98.0)	96.0 (93.0 to 97.0)	96.0 (93.0 to 98.0)
Respiratory rate on admission	229 421 (33.3)	Median (IQR)	20.0 (18.0 to 24.0)	21.0 (18.0 to 26.0)	20.0 (18.0 to 25.0)
Admission to ICU	674 753 (97.9)	Never	300 046 (87.6)	270 984 (81.6)	571 030 (84.6)
		Later admission	30 290 (8.8)	38 720 (11.7)	69 010 (10.2)
		Direct admission	12 212 (3.6)	22 501 (6.8)	34 713 (5.1)
Outcome	689 572 (100.0)	Unknown outcome	28 189 (8.1)	33 706 (9.9)	61 895 (9.0)
		Death	69 878 (20.0)	80 589 (23.7)	150 467 (21.8)
		Discharge	251 200 (71.9)	226 010 (66.4)	477 210 (69.2)
SARS-CoV-2 status	689 572 (100.0)	Positive	314 139 (89.9)	314 013 (92.3)	628 152 (91.1)
		Unknown	35 128 (10.1)	26 292 (7.7)	61 420 (8.9)

* Percentage of individuals with oxygen saturation recorded.

The majority of patients were recruited in South Africa (59.3%) and 31.1% in the UK (Table 1). Participants’ characteristics varied between patients admitted directly to an ICU, those admitted at a later time point and those never admitted (Supplementary Table S1, available as Supplementary data at *IJE* online). In particular there were differences in age, region and vital signs (heart and respiratory rate and blood pressure). Anthropometric variables and vital signs varied by age (Supplementary Table S2, available as Supplementary data at *IJE* online).

### Presenting symptoms

**Figure 3:**
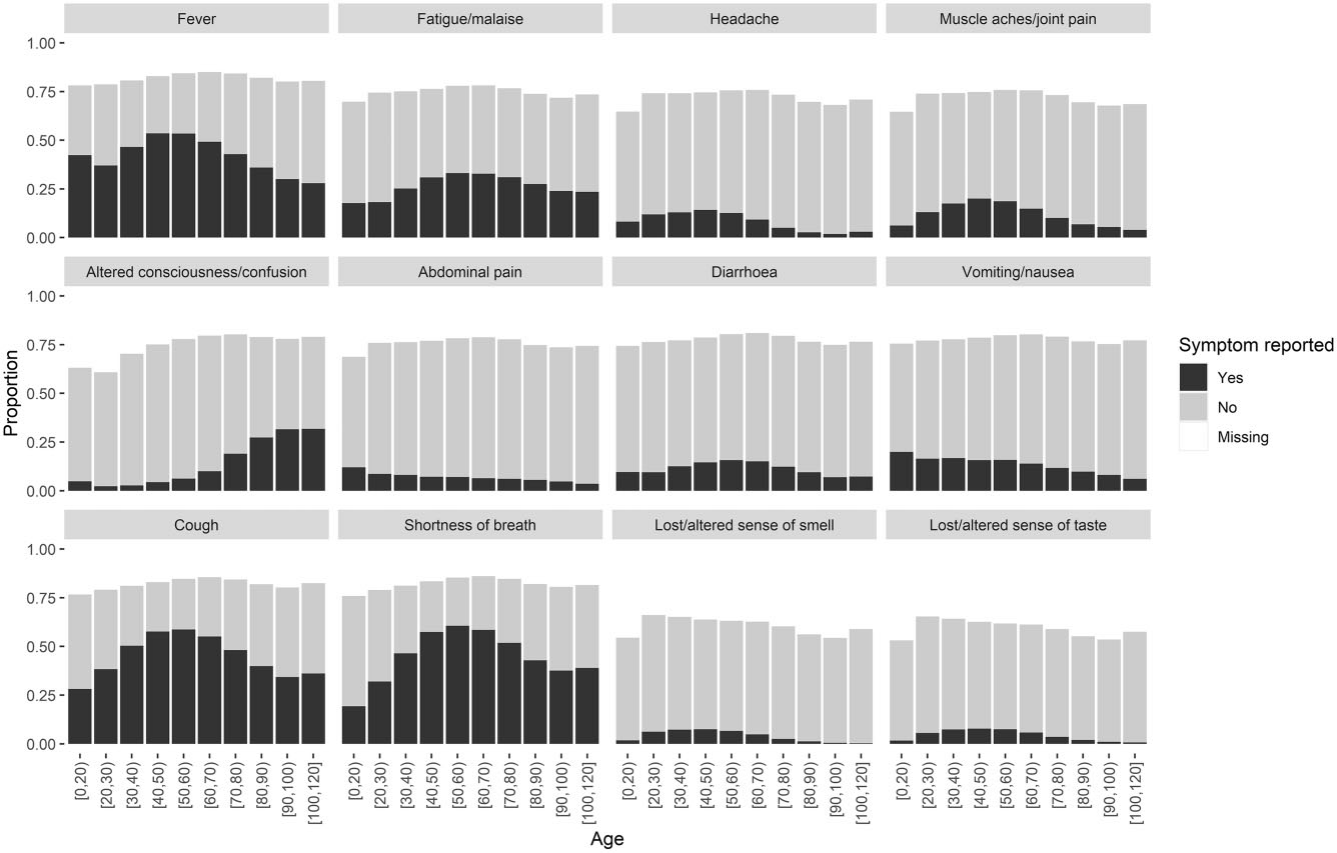
Symptom prevalence by age (*n* = 290 750)

The most common symptoms on presentation were shortness of breath (50%), cough (48.5%) and fever (44.3%) (Figure 3 and Supplementary Table S3, available as Supplementary data at *IJE* online). There was some variation by age and country (Supplementary Figure S2, available as Supplementary data at *IJE* online). Fatigue/malaise, cough and shortness of breath were most prevalent amongst patients who were 40–70 years old. The prevalence of altered consciousness/confusion increased with age and was reported in 28.4% of patients of >80 years of age. Loss or altered smell or taste were not commonly reported but there was a high proportion of missing values for these two symptoms (39.3% for loss of smell and 40.5% for taste). We have previously described the associations of age and gender with presenting symptoms.^11^ Prevalence of symptoms by age was similar when we restricted our analysis to patients with laboratory-confirmed SARS-CoV-2 (Supplementary Figure S3, available as Supplementary data at *IJE* online) but there were more missing values among individuals with only a clinical diagnosis (Supplementary Figure S4, available as Supplementary data at *IJE* online). Altered consciousness/confusion, cough, fatigue/malaise, fever, shortness of breath and vomiting/nausea were more frequently reported in patients with laboratory-confirmed SARS-CoV-2 infection than in those with a clinical diagnosis alone (Supplementary Figure S5, available as Supplementary data at *IJE* online).

**Figure 4:**
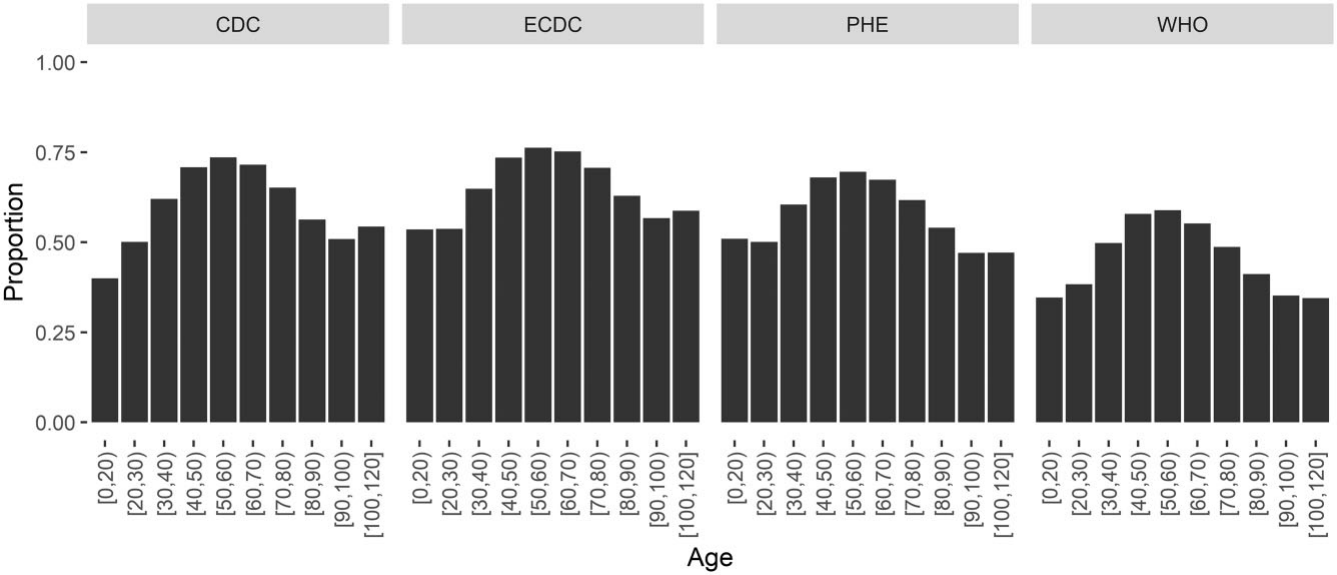
Proportions meeting each symptom definition by age (*n* = 290 750). CDC, Centers for Disease Control and Prevention; ECDC, European Centre for Disease Prevention and Control; PHE, Public Health England; WHO, World Health Organization

Overall, 50–70% of patients met each of the international symptom-based case definitions. These proportions were higher among those with laboratory-confirmed SARS-CoV-2 infection (CDC met by 64.0% vs 33.7% among laboratory-confirmed and clinically diagnosed, respectively, ECDC 68.7% vs 31.3%, PHE 61.3% vs 33.7%, WHO 49.2% vs 30.2%) (Supplementary Figure S6, available as Supplementary data at *IJE* online). Individuals aged 40–70 years were more likely to meet each of the four case definitions based on symptoms than patients at the extremes of the age distribution (Figure 4). Adults with laboratory-confirmed SARS-CoV-2 infection were more likely to meet one of the four case definitions based on symptoms than those with only a clinical diagnosis of SARS-CoV-2 infection, but the opposite was true amongst patients aged <20 years (Supplementary Figure S7, available as Supplementary data at *IJE* online).

Routine blood test results are shown in Supplementary Table S4 (available as Supplementary data at *IJE* online). The median white blood cell count (7.2$\times 10^{9};$ IQR 5.4–9.8$\times 10^{9}$ cells per litre) was normal but the median lymphocyte count was low (0.9$\times 10^{9};$ IQR 0.6–1.3$\times 10^{9}$ cells per litre) among participants with available data. The median C-reactive protein was high (74.0; IQR 30.0–138.0 mg/litre) but the median liver transaminase and median urea levels were unremarkable. Several median values for routine blood test results varied with age; with increasing age, the median lymphocyte count was lower, whereas the median urea was higher (Supplementary Figure S8, available as Supplementary data at *IJE* online).

### Pre-existing co-morbidities and risk factors

**Figure 5:**
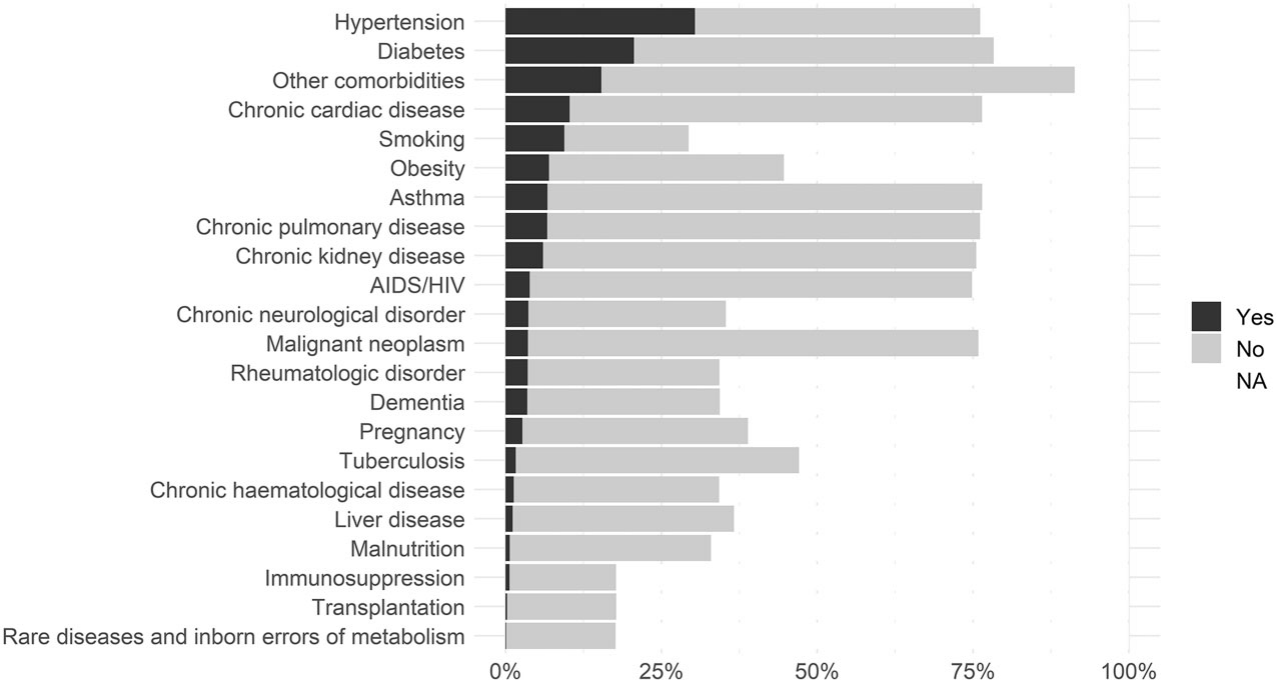
Prevalence of pre-existing co-morbidities and risk factors (*n* = 689 572)

The most common pre-existing co-morbidities were hypertension, diabetes and chronic cardiac disease (Figure 5 and Supplementary Table S5, available as Supplementary data at *IJE* online). Among 538 974 individuals with data available for any five or more co-morbidities or risk factors, 165 987 (30.8%) had no co-morbidities reported. The prevalence of most co-morbidities varied by age (Supplementary Figure S9, available as Supplementary data at *IJE* online). The prevalence of chronic cardiac disease, chronic kidney disease, dementia, hypertension and rheumatologic disorder increased with age. The prevalence of diabetes was highest in individuals aged 60–80 years. There were 26 776 patients with HIV infection, 11 384 with tuberculosis and 5044 with both; 25 368 of the patients with HIV infection and 11 137 patients with tuberculosis were from South Africa. Obesity was reported for 48 077 participants, smoking for 65 056 and pregnancy for 18 669.

### Treatments

Of 275 051 patients with available data on oxygen therapy (98.0% of individuals included in the treatment analyses), 179 448 (65.2%) received oxygen therapy at any time during hospitalization, which was delivered via a high-flow nasal cannula to 55 541 (19.8%), by non-invasive ventilation (NIV) to 44 280 (15.8%) and IMV to 38 440 (13.7%) (Table 2). Type of oxygen supplementation received was not specified for 48.7%. As might be expected, a proportion of patients received multiple types of oxygen delivery systems during their admission [37 330 (20.8%)]. For instance, 41.8% of those receiving IMV also received oxygen delivered via NIV.

**Table 2:** Oxygen supplementation among patients who received at least one type of oxygen supplementation

	*N* recorded (%)		Female	Male	Total
Total *N* (%)			72 484 (40.4)	106 964 (59.6)	179 448
Extracorporeal membrane oxygenation	173 972 (96.9)	No	69 984 (99.1)	102 052 (98.7)	172 036 (98.9)
		Yes	603 (0.9)	1333 (1.3)	1936 (1.1)
Ever received invasive mechanical ventilation	176 135 (98.2)	No	58 745 (82.5)	78 950 (75.2)	137 695 (78.2)
		Yes	12 462 (17.5)	25 978 (24.8)	38 440 (21.8)
Ever received non-invasive ventilation	177 313 (98.8)	No	56 111 (78.3)	76 922 (72.8)	133 033 (75.0)
		Yes	15 594 (21.7)	28 686 (27.2)	44 280 (25.0)
High-flow nasal cannula	163 908 (91.3)	No	45 488 (68.5)	62 879 (64.5)	108 367 (66.1)
		Yes	20 891 (31.5)	34 650 (35.5)	55 541 (33.9)
Oxygen therapy via mask	45 (0.0)	No	17 (89.5)	21 (80.8)	38 (84.4)
		Yes	2 (10.5)	5 (19.2)	7 (15.6)
Nasal oxygen therapy	45 (0.0)	No	0 (0.0)	2 (7.7)	2 (4.4)
		Yes	19 (100.0)	24 (92.3)	43 (95.6)
Other or unspecified type of oxygen supplementation	87 328 (48.7)		38 922 (100.0)	48 406 (100.0)	87 328 (100.0)
Number of types of oxygen supplementation	92 120 (51.3)	1	20 998 (62.6)	33 792 (57.7)	54 790 (59.5)
		2	9240 (27.5)	17 672 (30.2)	26 912 (29.2)
		3	3203 (9.5)	6836 (11.7)	10 039 (10.9)
		4	121 (0.4)	258 (0.4)	379 (0.4)

* Extracorporeal membrane oxygenation, invasive mechanical ventilation, non-invasive ventilation or high-flow nasal cannula.

**Figure 6:**
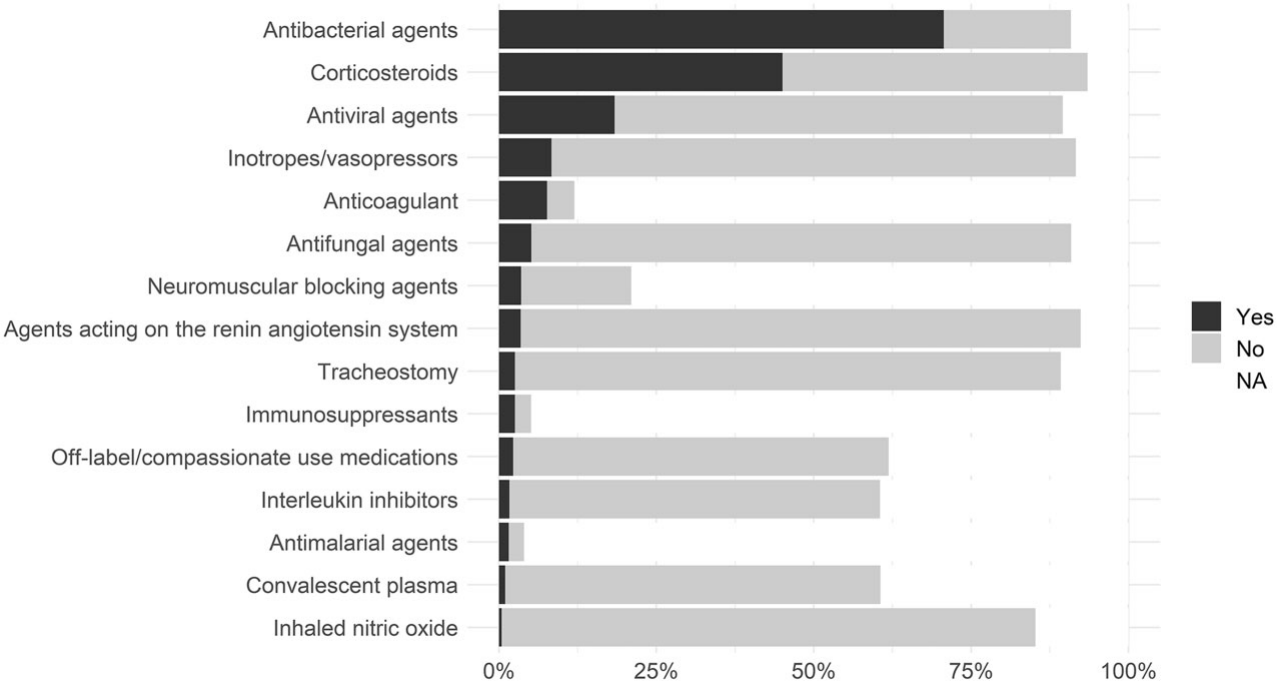
Proportion who have received each treatment (*n* = 290 750)

The most used treatments were oxygen therapy, antibacterial agents and corticosteroids (Figure 6 and Supplementary Table S6, available as Supplementary data at *IJE* online). The proportion of patients receiving antibacterial agents increased with age, as did the proportion receiving corticosteroids up to ages 70–80 years (Supplementary Figure S10, available as Supplementary data at *IJE* online). Information on antibacterial treatment was available for 255 031 patients, 198 295 (77.8%) of whom received antibacterial agents; 126 391 of 262 385 of patients with data available (48.2%) received corticosteroids. The use of corticosteroids increased after the publication of results of the RECOVERY trial^19^ in June 2020 (Supplementary Figure S11, available as Supplementary data at *IJE* online), in particular among patients who received oxygen supplementation, in line with the trial results.

### CFR

**Figure 7:**
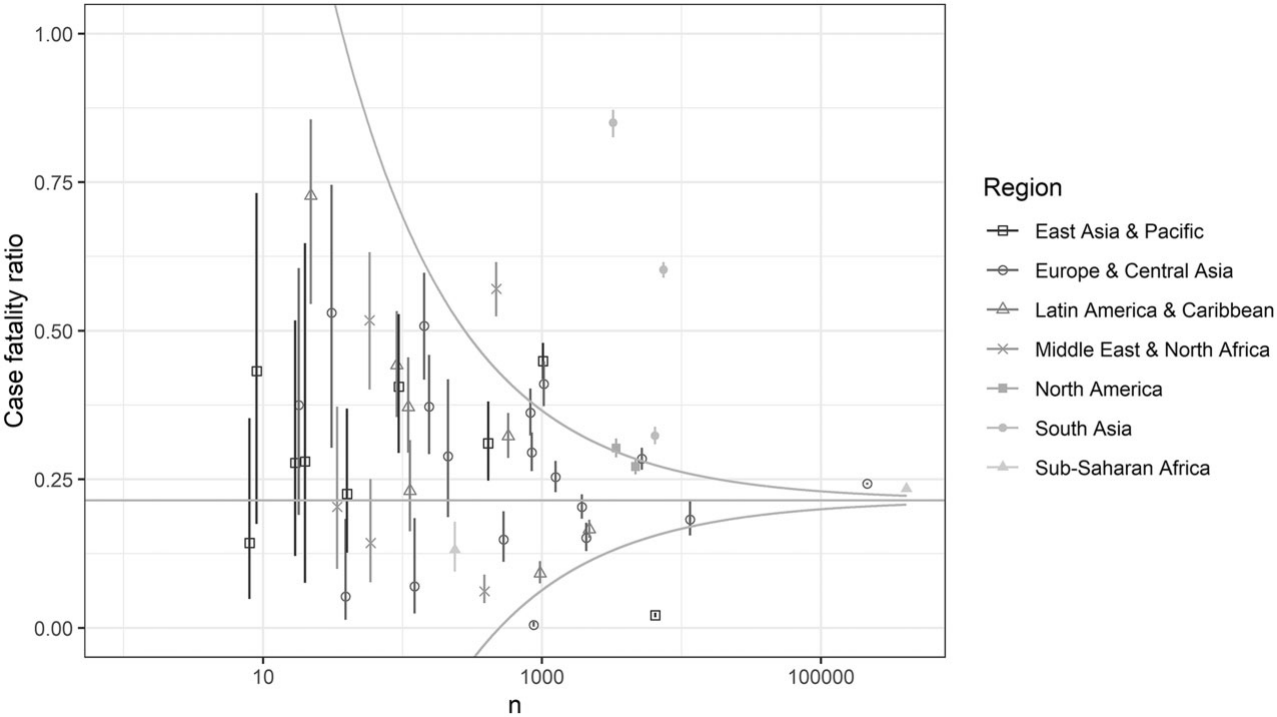
Case-fatality ratio by country. Each point is a country and points are coloured and have different shapes by region. The horizontal line is the inverse-variance weighted average case-fatality ratio. The funnel plot shows the 95% confidence limits. The *x*-axis is on a log10 scale

The CFR varied by country (Figure 7), likely because of the different features of different sites in different countries where patients of different disease severity may be admitted. The weighted average CFR was 0.215 [standard error (SE) 0.000462]. Among patients for whom reporting commenced in the ICU, the CFR was 0.469 (SE 0.00287). Among patients admitted to the ICU but for whom reporting did not commence in the ICU, the CFR was 0.341 (SE 0.00178). The CFR was 0.214 (SE 0.00048) among patients with laboratory-confimed SARS-CoV-2 and 0.231 (SE 0.00161) among patients with clinically diagnosed COVID-19. The CFR varied over time during the study, as did patient recruitment at different sites (Supplementary Figure S12, available as Supplementary data at *IJE* online). Admission criteria likely varied by country and time, contributing to the heterogeneity in illness severity. Death and discharge rates increased over the first 40 days from hospital admission (or symptom onset if this occurred after admission) (Supplementary Figure S13, available as Supplementary data at *IJE* online).

### Associations with death

**Figure 8:**
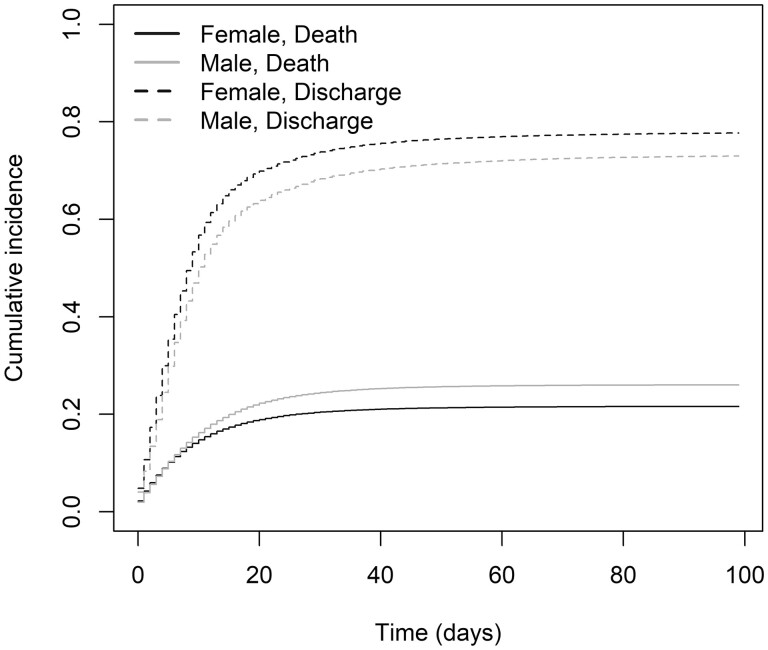
Cumulative incidence curves of death and discharge by sex (*n* = 689 572)

**Figure 9:**
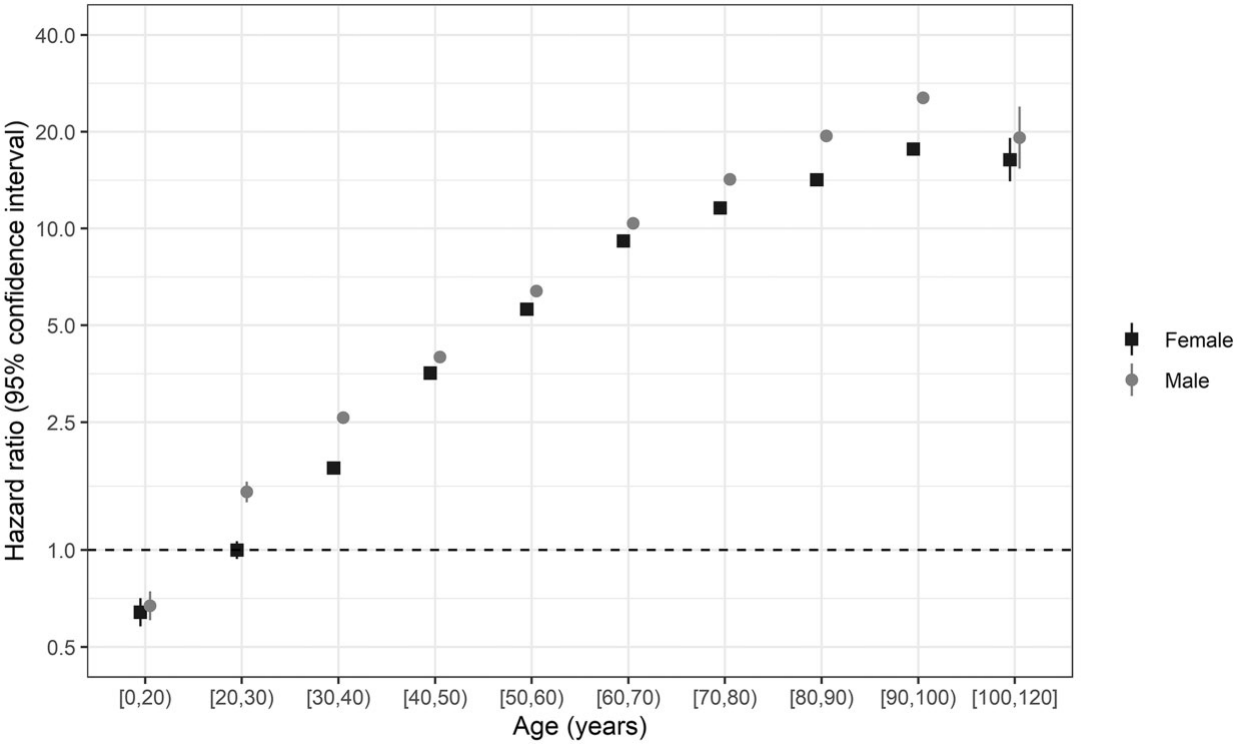
Hazard ratios and 95% confidence intervals for death by age group and sex (*n* = 689 572). The model is stratified by country. The reference group is females of age [20,30). The *y*-axis is plotted on a logarithmic scale

The risk of death was higher for males than for females (Figure 8). Older age was associated with a significantly higher risk of death, with a HR of 1.49 (95% CI 1.48, 1.49) per 10 years higher age, adjusting for sex and stratifying by country, with individuals aged 90–100 years having an HR of 17.45 (95% CI 16.54, 18.40) compared with the group aged 20–30 years (Supplementary Figure S14, available as Supplementary data at *IJE* online). Males had a significantly higher risk of death than females, with an HR of 1.23 (95% CI 1.21, 1.24), adjusting for age (in 10-year groups) and stratifying by country. There was evidence of deviation from the proportional hazards assumption for both variables. There was no particular trend over follow-up for the magnitude of the association of age with death, but the magnitude of the association of male sex with death appeared to increase with increasing time from admission (or symptom onset for patients who developed symptoms after admission). A model stratified by sex was fitted to estimate associations of age with death taking this time-varying association of sex into account. HRs for age estimated by the two models were very similar. HRs were also estimated by sex and age, stratifying (allowing different baseline hazards) by country (Figure 9). HRs for age (Supplementary Figure S15, available as Supplementary data at *IJE* online) and sex (Supplementary Figure S16, available as Supplementary data at *IJE* online) varied by country. Country-specific HRs for a 10-year increase in age varied from 1.15 (Italy; 95% CI 1.05, 1.26) to 3.50 (Poland; 1.19, 10.29), with a pooled estimate of 1.49 (1.48, 1.49), which is the same as the estimate from the main analysis. For sex (males vs females), HRs were between 0.61 (Malawi; 95% CI 0.28, 1.31) and 3.57 (Japan; 1.05, 12.07), with a weighted average of 1.26 (1.25, 1.27) [compared with 1.23 (1.21, 1.24) in the main analysis]. When fitting a model including age (continuous) and sex, and stratified by country, within each year of the pandemic, HRs (95% CI) per 10 years of higher age were 1.50 (1.49, 1.51) for 2020 and 1.47 (1.47, 1.48) for 2021, and for males vs females 1.31 (1.29, 1.33) and 1.20 (1.19, 1.22), respectively.

**Figure 10:**
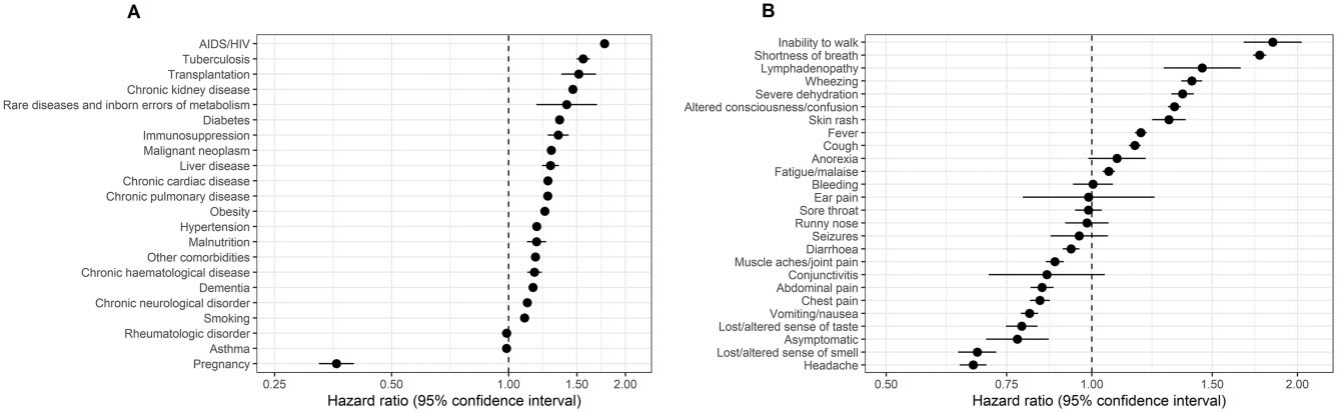
Associations of (A) co-morbidities (*n* = 689 572) and (B) symptoms (*n* = 290 750) with risk of death. Dots are hazard ratios and lines are 95% confidence intervals of death by each variable at a time (the reference group is not having the particular symptom/comorbidity/risk factor). Models were adjusted for age and age^2^, stratified by sex and country

Overall, HIV infection [HR 1.77 (95% CI 1.72, 1.81), adjusting for age, age^2^ and sex, and stratifying by country] and tuberculosis [1.56 (1.50, 1.62)] were associated with the highest relative risks of death; there was no evidence of an interaction (*P* = 0.18). Since many patients with these co-morbidities were from South Africa, we assessed the associations separately among patients from South Africa and patients from all other countries by fitting a model including HIV, tuberculosis, age and age stratified by sex and country. The associations with risk of death did not significantly differ between patients from South Africa and patients from other countries, although estimates were numerically higher for patients from South Africa: respectively for HIV HR (95% CI) 1.52 (1.47, 1.58) and 1.18 (0.83, 1.69) and for tuberculosis 1.35 (1.29, 1.41) and 1.18 (0.82, 1.70). All reported co-morbidities were associated with a higher risk of death, except rheumatologic disorder and asthma for which there was no evidence of an association (Figure 10 and Supplementary Table S7, available as Supplementary data at *IJE* online). Obesity was associated with a higher risk of death [HR 1.24 (1.21, 1.27)]. Smoking was also associated with a higher risk of death [1.10 (1.07, 1.12)]. Pregnancy was associated with a lower risk of death [HR 0.37 (0.33, 0.41) among females aged 15–45 years, adjusting for age, age^2^ and stratifying by country]. There were small differences in the magnitude of associations of co-morbidities with risk of death between calendar years (Supplementary Figure S17, available as Supplementary data at *IJE* online).

Inability to walk [HR 1.84 (95% CI 1.67, 2.03), adjusting for age, age^2^ and sex, and stratifying by country], shortness of breath [1.76 (1.72, 1.80)], lymphadenopathy [1.45 (1.27, 1.65)], wheezing [1.40 (1.35, 1.45)], severe dehydration [1.36 (1.31, 1.41)], altered consciousness/confusion [1.32 (1.29, 1.35)], skin rash [1.30 (1.22, 1.37)], cough [1.16 (1.13, 1.18)], fever [1.18 (1.16, 1.20)] and fatigue/malaise [1.06 (1.04, 1.08)] were associated with a higher risk of death (Figure 10 and Supplementary Table S8, available as Supplementary data at *IJE* online). In general, gastrointestinal, musculoskeletal symptoms and loss of or altered taste or smell were associated with a lower risk of dying; e.g. nausea/vomiting had a HR of 0.81 (0.79, 0.83), abdominal pain 0.84 (0.81, 0.88), diarrhoea 0.93 (0.91, 0.96) and muscle aches/joint pain 0.88 (0.86, 0.91).

### Associations with admission to an ICU and with use of IMV

**Figure 11:**
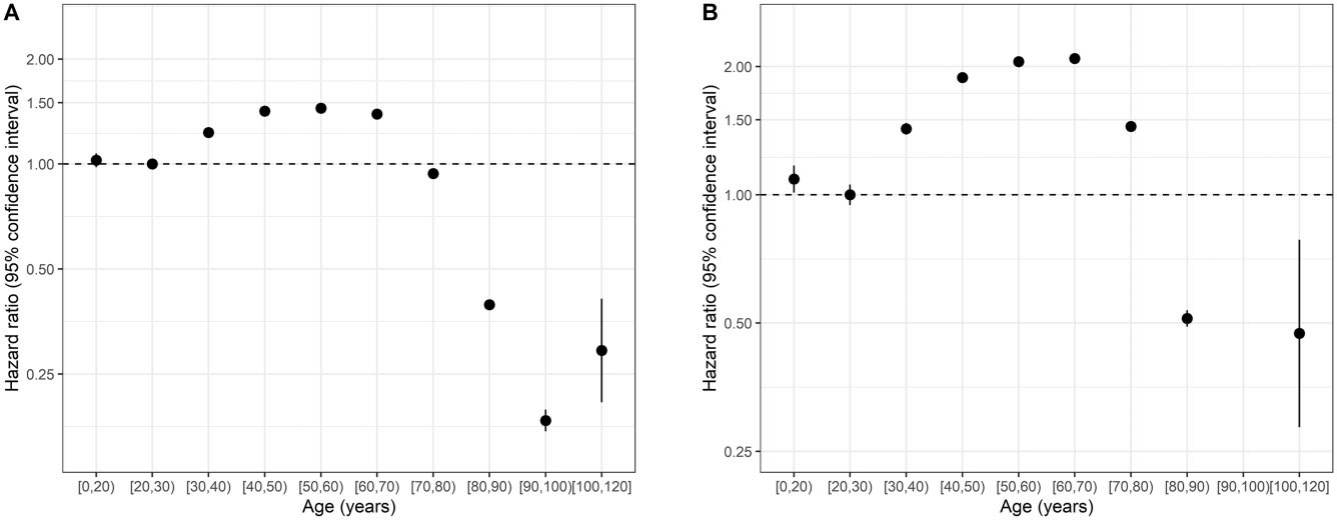
Hazard ratios and 95% confidence intervals for (A) admission to an intensive care unit and (B) use of invasive mechanical ventilation by age (*n* = 689 572)

The risk of admission to an ICU increased with age after age 30 years and started decreasing from age 60 years, with patients aged >80 years being unlikely to be admitted to an ICU. Men were more likely to be admitted to an ICU overall, with a HR of 1.17 (1.15, 1.18). There was evidence of non-proportional hazards, indicating that the relative risk changed with time since symptom onset (or hospitalization). There were similar patterns for risk of IMV (Figure 11). There was some variation in risks by age in different countries (Supplementary Figure S18, available as Supplementary data at *IJE* online).

## Discussion

The ISARIC international cohort study included at the time of these analyses standardized data on almost 700 000 patients from 1380 sites across 52 countries. To our knowledge, this is the most extensive in-hospital COVID-19 cohort study in the world. The size and breadth of the study allow us to evaluate the contribution of individual risk factors to outcomes such as death, admission to an ICU and use of mechanical ventilation. The value of the international cohort design is its capacity to cover the breadth of COVID-19 characteristics unencumbered by differences in classification and reporting. Furthermore, our international cohort design allowed us to explore risk factors that are globally uncommon, or uncommon in cohorts from high-income countries. For example, our data set is the largest prospective cohort study of COVID-19 patients with HIV infection, tuberculosis, malnutrition, pregnancy and transplantation. Although the study population includes children and we have presented some of their characteristics, a detailed analysis of the children population is beyond the scope of this paper.

Across the cohort, the most common presenting symptoms were fever, shortness of breath and cough. Among other symptoms reported, the most common were altered consciousness in older patients and gastrointestinal symptoms in younger patients. Our data show that about one-third of patients do not meet one of the four most widely used case definitions at the time of hospitalization, particularly those in the younger and older age groups. These differences are relevant when defining testing or isolation and for early detection of new clusters and variants. Although case definitions must be simple, age-specific definitions may improve sensitivity. This has implications also for case management; about one-third of patients did not require any oxygen therapy during their hospitalization.

Our study confirms that the strong association between age and risk of death from COVID-19 is a global phenomenon. The elderly are at a significantly higher risk of death from COVID-19. Every decade of life adds a 50% risk of dying, with those aged >90 years having a 17-fold higher risk than 20- to 30-year-olds. Although similar results were shown globally for non-hospitalized cases,^20^^,^^21^ our study reproduces these results globally and amongst hospitalized patients. There were differences between the sexes, with men having an increased risk of death around one-third higher than the corresponding female 10-year age group. A meta-analysis published early in the pandemic showed that male patients had an odds ratio (OR) of 2.84 (95% CI 2.06, 3.92) for intensive treatment unit admission and an OR of 1.39 (1.31, 1.47) for death compared with females, despite not having a higher risk of infection.^22^ A US study of hospitalized patients also reported a higher risk of death among males.^23^ Such age- and sex-specific CFRs with a global perspective are critical to understanding the global in-hospital burden of COVID-19. The pattern holds across lower- and higher-income countries. Interestingly, non-respiratory presentations were associated with lower risk of death.

We found five co-morbidities to be strongly associated with risk of death. The most substantial risk factor was HIV infection. There was a high proportion of people living with HIV (PLWH) in this cohort. Whilst retrospective health records analyses have been performed previously,^24^^,^^25^ this is the most extensive international cohort study of COVID-19 in PLWH. A recent cohort study performed in South Africa^26^ demonstrated that PLWH had an adjusted OR of death of 1.34 (95% CI 1.27, 1.43). Our study reinforces these findings and suggests that a higher risk is observed in other countries as well. Unfortunately, we have no further detail on how well controlled the HIV infection was or on the levels of immunocompromise for PLWH in our study. The second strongest association with the risk of death was a diagnosis of tuberculosis. To our knowledge, this is also the largest international cohort of patients co-infected with SARS-CoV-2 and tuberculosis (>5000 patients); however other studies have reported a higher risk of death among patients with COVID-19 and tuberculosis.^26^^,^^27^ There have been few studies on the effect of COVID-19 on transplant patients. The 1606 transplant patients included in our data set make it one of the largest cohorts to date. Overall, risk of death was 52% higher in transplant patients and 34% higher in those on immunosuppressive therapies. Several studies have shown that tobacco smoking is associated with worse COVID-19 outcomes.^28^^,^^29^ We found a 10% higher risk of death among smokers; however, smoking information was available only on ∼30% of patients and detailed information on the quantity and duration of smoking were not available. Obesity is a recognized risk factor for hospitalization and poor outcomes in patients with COVID-19 and has been one of the conditions for which individuals are prioritized for access to vaccines and treatments. Obesity was associated with a 24% increase in risk; obesity status was available for ∼45% of patients. Both smoking and obesity are potentially modifiable risk factors that could be targeted by public health measures. Our results suggest pregnancy is associated with a lower risk of death among people admitted to hospital, which appears to contrast with other studies suggesting an increased risk of death, intubation or ICU admission for pregnant women.^30^ However, the UK Obstetric Surveillance System found that 55% of hospital admissions for pregnant women with COVID-19 were for the purpose of giving birth,^31^ whereas very few other elective and semi-elective admissions were taking place during the pandemic; this is likely to have increased the proportion of pregnant women in hospital with less severe COVID-19 compared with the broader cohort, confounding our observed lower risk of death for pregnant women.

Globally, CFRs were much higher in the 5% of patients who were admitted to an ICU on the first day of their admission than those who required an ICU later during their admission. The risk of admission to an ICU increased with age, but then started decreasing from age 60 years, with patients aged >80 years being very unlikely to be admitted to an ICU. It has been suggested that patients who have severe illness and are not treated in an ICU have poorer outcomes.^32^ This may reflect decisions to admit patients to an ICU during periods when resources were limited, taking into account their overall health. Compared with other studies, these results are consistent for patients aged <60 years but not for those aged >60 years. For example, in a study from the USA^33^ and in a separate meta-analysis,^34^ elderly patients were more likely to be admitted to an ICU than their younger counterparts;^35^ this may reflect geographical variation in clinical practice.

This international cohort study overcomes some of the traditional problems of multicentre observational studies by using standardized variables and outcome measures. Our data are likely to be of value in modelling and health system planning. For example, we note the greatly increased risk of death amongst patients with tuberculosis and malnutrition in our cohort and protecting such individuals from COVID-19 must be a critical public health priority for countries with high prevalence rates of these conditions. Equally concerning is our finding of increased risk of death amongst PLWH. Many PLWH reside in sub-Saharan Africa and our data may indicate a phenomenon that is currently hidden due to under-testing for SARS-CoV-2^36^ across Africa.

### Limitations

Whereas our study includes a broad range of data from different countries, various sites have different levels of data completeness. For example, we cannot evaluate the proportion of patients with HIV infection or tuberculosis who were taking appropriate, effective treatments. We have no further detail on the type of organ received by the transplantation cohort. Missing data may have affected the estimates of prevalence of symptoms, co-morbidities and other patient characteristics, as well as estimates of associations between these and risks of outcomes. We presented missing data in each variable in tables and figures. Specifically for symptoms and treatments, data from South Africa were not available and aggregated estimates might largely reflect data from the UK. Whereas we have produced a summary of the associations of risk factors with outcomes in COVID-19, pandemics are complex, dynamic phenomena. There is variation in the amount and completeness of data between countries and between sites within each country. Although we have adjusted for potential confounders, inclusion of patients in the data varies by country/site, which may lead to bias in estimates of association and of absolute risks such as CFR. Our findings will increasingly be influenced by the provision of vaccination, which we have not examined in this study, and effective treatments, as well as the variability in access to these measures in the global context. We do not include data on SARS-CoV-2 variants of concern in this paper. The majority of submitted case records come from two countries: the UK and South Africa; however, there were 22 countries with data on >500 patients.

## Conclusion

This paper represents the largest international cohort of hospitalized COVID-19 patients published to date. We demonstrate several associations of global importance, including an increased risk of death in patients with HIV and tuberculosis. Co-morbidities were associated with a higher risk of death, each associated with up to a 2-fold increase. Smoking and obesity were also associated with a higher risk of death. Age was most strongly associated with risk of death, with a ∼30-fold difference between the oldest and youngest groups. These findings may be used to inform strategies that involve prioritization of high-risk patients hospitalized with COVID-19 and prevention strategies. The ISARIC global collaboration continues to collect standardized data which will enable continued data-led comparisons as the world implements vaccination, treatment and public health control strategies.

## Notes

ISARIC Clinical Characterisation Group: Ali Abbas; Sheryl Ann Abdukahil; Nurul Najmee Abdulkadir; Ryuzo Abe; Laurent Abel; Lara Absil; Subhash Acharya; Andrew Acker; Elisabeth Adam; Diana Adrião; Saleh Al Ageel; Shakeel Ahmed; Kate Ainscough; Eka Airlangga; Tharwat Aisa; Ali Ait Hssain; Younes Ait Tamlihat; Takako Akimoto; Ernita Akmal; Eman Al Qasim; Razi Alalqam; Angela Alberti; Tala Al-dabbous; Senthilkumar Alegesan; Cynthia Alegre; Marta Alessi; Beatrice Alex; Kévin Alexandre; Abdulrahman Al-Fares; Huda Alfoudri; Imran Ali; Adam Ali; Naseem Ali Shah; Kazali Enagnon Alidjnou; Jeffrey Aliudin; Qabas Alkhafajee; Clotilde Allavena; Nathalie Allou; Aneela Altaf; João Alves; Rita Alves; João Melo Alves; Maria Amaral; Nur Amira; Phoebe Ampaw; Roberto Andini; Claire Andréjak; Andrea Angheben; François Angoulvant; Séverine Ansart; Sivanesen Anthonidass; Massimo Antonelli; Carlos Alexandre Antunes de Brito; Ardiyan Apriyana; Yaseen Arabi; Irene Aragao; Francisco Arancibia; Carolline Araujo; Antonio Arcadipane; Patrick Archambault; Lukas Arenz; Jean-Benoît Arlet; Christel Arnold-Day; Lovkesh Arora; Rakesh Arora; Elise Artaud-Macari; Diptesh Aryal; Angel Asensio; Muhammad Ashraf; Namra Asif; Mohammad Asim; Jean Baptiste Assie; Amirul Asyraf; Anika Atique; AM Udara Lakshan Attanyake; Johann Auchabie; Hugues Aumaitre; Adrien Auvet; Laurène Azemar; Cecile Azoulay; Benjamin Bach; Delphine Bachelet; Claudine Badr; Nadia Baig; J Kevin Baird; Erica Bak; Agamemnon Bakakos; Nazreen Abu Bakar; Andriy Bal; Mohanaprasanth Balakrishnan; Valeria Balan; Firouzé Bani-Sadr; Renata Barbalho; Nicholas Yuri Barbosa; Wendy S. Barclay; Saef Umar Barnett; Michaela Barnikel; Helena Barrasa; Audrey Barrelet; Cleide Barrigoto; Marie Bartoli; Mustehan Bashir; Romain Basmaci; Muhammad Fadhli Hassin Basri; Denise Battaglini; Jules Bauer; Diego Fernando Bautista Rincon; Denisse Bazan Dow; Alexandra Bedossa; Ker Hong Bee; Husna Begum; Sylvie Behilill; Albertus Beishuizen; Aleksandr Beljantsev; David Bellemare; Anna Beltrame; Beatriz Amorim Beltrão; Marine Beluze; Nicolas Benech; Lionel Eric Benjiman; Dehbia Benkerrou; Suzanne Bennett; Luís Bento; Jan-Erik Berdal; Delphine Bergeaud; Hazel Bergin; José Luis Bernal Sobrino; Giulia Bertoli; Lorenzo Bertolino; Simon Bessis; Sybille Bevilcaqua; Karine Bezulier; Amar Bhatt; Krishna Bhavsar; Claudia Bianco; Farah Nadiah Bidin; Moirangthem Bikram Singh; Felwa Bin Humaid; Mohd Nazlin Bin Kamarudin; François Bissuel; Patrick Biston; Laurent Bitker; Jonathan Bitton; Pablo Blanco-Schweizer; Catherine Blier; Frank Bloos; Mathieu Blot; Filomena Boccia; Laetitia Bodenes; Alice Bogaarts; Debby Bogaert; Anne-Hélène Boivin; Pierre-Adrien Bolze; François Bompart; Aurelius Bonfasius; Diogo Borges; Raphaël Borie; Hans Martin Bosse; Elisabeth Botelho-Nevers; Lila Bouadma; Olivier Bouchaud; Sabelline Bouchez; Dounia Bouhmani; Damien Bouhour; Kévin Bouiller; Laurence Bouillet; Camile Bouisse; Anne-Sophie Boureau; John Bourke; Maude Bouscambert; Aurore Bousquet; Jason Bouziotis; Bianca Boxma; Marielle Boyer-Besseyre; Maria Boylan; Axelle Braconnier; Cynthia Braga; Timo Brandenburger; Filipa Brás Monteiro; Luca Brazzi; Patrick Breen; Dorothy Breen; Patrick Breen; Kathy Brickell; Shaunagh Browne; Alex Browne; Nicolas Brozzi; Marjolein Brusse-Keizer; Nina Buchtele; Christian Buesaquillo; Polina Bugaeva; Marielle Buisson; Danilo Buonsenso; Erlina Burhan; Ingrid G. Bustos; Denis Butnaru; André Cabie; Susana Cabral; Eder Caceres; Cyril Cadoz; Mia Callahan; Kate Calligy; Jose Andres Calvache; João Camões; Valentine Campana; Paul Campbell; Josie Campisi; Cecilia Canepa; Mireia Cantero; Pauline Caraux-Paz; Sheila Cárcel; Chiara Simona Cardellino; Sofia Cardoso; Filipe Cardoso; Filipa Cardoso; Nelson Cardoso; Simone Carelli; Nicolas Carlier; Thierry Carmoi; Gayle Carney; Inês Carqueja; Marie-Christine Carret; François Martin Carrier; Ida Carroll; Maire-Laure Casanova; Mariana Cascão; Siobhan Casey; José Casimiro; Bailey Cassandra; Silvia Castañeda; Nidyanara Castanheira; Guylaine Castor-Alexandre; Henry Castrillón; Ivo Castro; Ana Catarino; François-Xavier Catherine; Paolo Cattaneo; Roberta Cavalin; Giulio Giovanni Cavalli; Alexandros Cavayas; Adrian Ceccato; Minerva Cervantes-Gonzalez; Anissa Chair; Catherine Chakveatze; Adrienne Chan; Meera Chand; Christelle Chantalat Auger; Jean-Marc Chapplain; Julie Chas; Allegra Chatterjee; Mobin Chaudry; Jonathan Samuel Chávez Iñiguez; Anjellica Chen; Yih-Sharng Chen; Matthew Pellan Cheng; Antoine Cheret; Thibault Chiarabini; Julian Chica; Suresh Kumar Chidambaram; Leong Chin Tho; Catherine Chirouze; Davide Chiumello; Sung-Min Cho; Bernard Cholley; Marie-Charlotte Chopin; Ting Soo Chow; Yock Ping Chow; Hiu Jian Chua; Jonathan Chua; Jose Pedro Cidade; José Miguel Cisneros Herreros; Anna Ciullo; Jennifer Clarke; Emma Clarke; Rolando Claure-Del Granado; Sara Clohisey; Perren J. Cobb; Cassidy Codan; Caitriona Cody; Alexandra Coelho; Megan Coles; Gwenhaël Colin; Michael Collins; Sebastiano Maria Colombo; Pamela Combs; Marie Connor; Anne Conrad; Sofía Contreras; Elaine Conway; Graham S. Cooke; Mary Copland; Hugues Cordel; Amanda Corley; Sabine Cornelis; Alexander Daniel Cornet; Arianne Joy Corpuz; Andrea Cortegiani; Grégory Corvaisier; Emma Costigan; Camille Couffignal; Sandrine Couffin-Cadiergues; Roxane Courtois; Stéphanie Cousse; Rachel Cregan; Charles Crepy D'Orleans; Cosimo Cristella; Sabine Croonen; Gloria Crowl; Jonathan Crump; Claudina Cruz; Juan Luis Cruz Berm; Jaime Cruz Rojo; Marc Csete; Ailbhe Cullen; Matthew Cummings; Gerard Curley; Elodie Curlier; Colleen Curran; Paula Custodio; Ana da Silva Filipe; Charlene Da Silveira; Al-Awwab Dabaliz; Darren Dahly; Heidi Dalton; Jo Dalton; Seamus Daly; Nick Daneman; Corinne Daniel; Jorge Dantas; Frédérick D'Aragon; Menno de Jong; Gillian de Loughry; Diego de Mendoza; Etienne De Montmollin; Rafael Freitas de Oliveira França; Ana Isabel de Pinho Oliveira; Rosanna De Rosa; Cristina De Rose; Thushan de Silva; Peter de Vries; Jillian Deacon; David Dean; Alexa Debard; Marie-Pierre Debray; Nathalie DeCastro; William Dechert; Lauren Deconninck; Romain Decours; Eve Defous; Isabelle Delacroix; Eric Delaveuve; Karen Delavigne; Nathalie M Delfos; Ionna Deligiannis; Andrea Dell'Amore; Christelle Delmas; Pierre Delobel; Corine Delsing; Elisa Demonchy; Emmanuelle Denis; Dominique Deplanque; Pieter Depuydt; Mehul Desai; Diane Descamps; Mathilde Desvallées; Santi Dewayanti; Pathik Dhanger; Alpha Diallo; Sylvain Diamantis; André Dias; Juan Jose Diaz; Priscila Diaz; Rodrigo Diaz; Kévin Didier; Jean-Luc Diehl; Wim Dieperink; Jérôme Dimet; Vincent Dinot; Fara Diop; Alphonsine Diouf; Yael Dishon; Félix Djossou; Annemarie B. Docherty; Helen Doherty; Arjen M Dondorp; Andy Dong; Maria Donnelly; Sean Donohue; Yoann Donohue; Chloe Donohue; Peter Doran; Céline Dorival; Eric D'Ortenzio; James Joshua Douglas; Renee Douma; Nathalie Dournon; Triona Downer; Joanne Downey; Mark Downing; Tom Drake; Aoife Driscoll; Murray Dryden; Murray Dryden; Claudio Duarte Fonseca; Vincent Dubee; François Dubos; Alexandre Ducancelle; Toni Duculan; Susanne Dudman; Abhijit Duggal; Paul Dunand; Mathilde Duplaix; Emanuele Durante-Mangoni; Lucian Durham III; Bertrand Dussol; Juliette Duthoit; Xavier Duval; Anne Margarita Dyrhol-Riise; Sim Choon Ean; Marco Echeverria-Villalobos; Siobhan Egan; Carla Eira; Mohammed El Sanharawi; Subbarao Elapavaluru; Brigitte Elharrar; Jacobien Ellerbroek; Philippine Eloy; Tarek Elshazly; Iqbal Elyazar; Isabelle Enderle; Tomoyuki Endo; Chan Chee Eng; Ilka Engelmann; Vincent Enouf; Olivier Epaulard; Mariano Esperatti; Hélène Esperou; Marina Esposito-Farese; João Estevão; Long COVID India Etienne; Manuel Etienne; Nadia Ettalhaoui; Anna Greti Everding; Mirjam Evers; Marc Fabre; Isabelle Fabre; Amna Faheem; Arabella Fahy; Cameron J. Fairfield; Zul Fakar; Komal Fareed; Pedro Faria; Ahmed Farooq; Hanan Fateena; Arie Zainul Fatoni; Karine Faure; Raphaël Favory; Mohamed Fayed; Niamh Feely; Laura Feeney; Jorge Fernandes; Marília Andreia Fernandes; Susana Fernandes; François-Xavier Ferrand; Eglantine Ferrand Devouge; Joana Ferrão; Mário Ferraz; Sílvia Ferreira; Isabel Ferreira; Benigno Ferreira; Ricard Ferrer-Roca; Nicolas Ferriere; Céline Ficko; Claudia Figueiredo-Mello; William Finlayson; Juan Fiorda; Thomas Flament; Clara Flateau; Tom Fletcher; Letizia Lucia Florio; Deirdre Flynn; Claire Foley; Jean Foley; Victor Fomin; Tatiana Fonseca; Patricia Fontela; Simon Forsyth; Denise Foster; Giuseppe Foti; Erwan Fourn; Robert A. Fowler; Marianne Fraher; Diego Franch-Llasat; John F Fraser; Christophe Fraser; Marcela Vieira Freire; Ana Freitas Ribeiro; Caren Friedrich; Ricardo Fritz; Stéphanie Fry; Nora Fuentes; Masahiro Fukuda; Argin G; Valérie Gaborieau; Rostane Gaci; Massimo Gagliardi; Jean-Charles Gagnard; Amandine Gagneux-Brunon; Sérgio Gaião; Linda Gail Skeie; Phil Gallagher; Carrol Gamble; Yasmin Gani; Arthur Garan; Rebekha Garcia; Julia Garcia-Diaz; Esteban Garcia-Gallo; Navya Garimella; Denis Garot; Valérie Garrait; Basanta Gauli; Nathalie Gault; Aisling Gavin; Anatoliy Gavrylov; Alexandre Gaymard; Johannes Gebauer; Eva Geraud; Louis Gerbaud Morlaes; Nuno Germano; praveen kumar ghisulal; Jade Ghosn; Marco Giani; Jess Gibson; Tristan Gigante; Morgane Gilg; Elaine Gilroy; Guillermo Giordano; Michelle Girvan; Valérie Gissot; Daniel Glikman; Petr Glybochko; Eric Gnall; Geraldine Goco; François Goehringer; Siri Goepel; Jin Yi Goh; Jonathan Golob; Rui Gomes; Kyle Gomez; Joan Gómez-Junyent; Marie Gominet; Alicia Gonzalez; Patricia Gordon; Isabelle Gorenne; Laure Goubert; Cécile Goujard; Tiphaine Goulenok; Margarite Grable; Jeronimo Graf; Edward Wilson Grandin; Pascal Granier; Giacomo Grasselli; Christopher A. Green; Courtney Greene; William Greenhalf; Segolène Greffe; Domenico Luca Grieco; Matthew Griffee; Fiona Griffiths; Ioana Grigoras; Albert Groenendijk; Anja Grosse Lordemann; Heidi Gruner; Yusing Gu; Jérémie Guedj; Martin Guego; Dewi Guellec; Anne-Marie Guerguerian; Daniela Guerreiro; Romain Guery; Anne Guillaumot; Laurent Guilleminault; Maisa Guimarães de Castro; Thomas Guimard; Marieke Haalboom; Daniel Haber; Hannah Habraken; Ali Hachemi; Amy Hackmann; Nadir Hadri; Fakhir Haidri; Sheeba Hakak; Adam Hall; Sophie Halpin; Jawad Hameed; Ansley Hamer; Raph L. Hamers; Rebecca Hamidfar; Terese Hammond; Lim Yuen Han; Rashan Haniffa; Kok Wei Hao; Hayley Hardwick; Ewen M Harrison; Janet Harrison; Samuel Bernard Ekow Harrison; Alan Hartman; Mohd Shahnaz Hasan; Junaid Hashmi; Muhammad Hayat; Ailbhe Hayes; Leanne Hays; Jan Heerman; Lars Heggelund; Ross Hendry; Martina Hennessy; Aquiles Henriquez-Trujillo; Maxime Hentzien; Jaime Hernandez-Montfort; Andrew Hershey; Liv Hesstvedt; Astarini Hidayah; Eibhilin Higgins; Dawn Higgins; Rupert Higgins; Rita Hinchion; Samuel Hinton; Hiroaki Hiraiwa; Haider Hirkani; Hikombo Hitoto; Yi Bin Ho; Alexandre Hoctin; Isabelle Hoffmann; Wei Han Hoh; Oscar Hoiting; Rebecca Holt; Jan Cato Holter; Juan Pablo Horcajada; Koji Hoshino; Ikram Houas; Catherine L. Hough; Stuart Houltham; Jimmy Ming-Yang Hsu; Jean-Sébastien Hulot; Stella Huo; Abby Hurd; Iqbal Hussain; Samreen Ijaz; Hajnal-Gabriela Illes; Patrick Imbert; Mohammad Imran; Rana Imran Sikander; Aftab Imtiaz; Hugo Inácio; Carmen Infante Dominguez; Yun Sii Ing; Elias Iosifidis; Mariachiara Ippolito; Sarah Isgett; Tiago Isidoro; Nadiah Ismail; Margaux Isnard; Junji Itai; Daniel Ivulich; Danielle Jaafar; Salma Jaafoura; Julien Jabot; Clare Jackson; Nina Jamieson; Victoria Janes; Pierre Jaquet; Coline Jaud-Fischer; Stéphane Jaureguiberry; Jeffrey Javidfar; Denise Jaworsky; Florence Jego; Anilawati Mat Jelani; Synne Jenum; Ruth Jimbo-Sotomayor; Ong Yiaw Joe; Ruth N. Jorge García; Cédric Joseph; Mark Joseph; Swosti Joshi; Mercé Jourdain; Philippe Jouvet; Hanna Jung; Anna Jung; Dafsah Juzar; Ouifiya Kafif; Florentia Kaguelidou; Neerusha Kaisbain; Thavamany Kaleesvran; Sabina Kali; Alina Kalicinska; Smaragdi Kalomoiri; Muhammad Aisar Ayadi Kamaluddin; Zul Amali Che Kamaruddin; Nadiah Kamarudin; Paul Kambiya; Kavita Kamineni; Darshana Hewa Kandamby; Chris Kandel; Kong Yeow Kang; Darakhshan Kanwal; Pratap Karpayah; Todd Karsies; Daisuke Kasugai; Anant Kataria; Kevin Katz; Aasmine Kaur; Christy Kay; Hannah Keane; Seán Keating; Pulak Kedia; Claire Kelly; Yvelynne Kelly; Andrea Kelly; Niamh Kelly; Aoife Kelly; Sadie Kelly; Maeve Kelsey; Ryan Kennedy; Kalynn Kennon; Maeve Kernan; Younes Kerroumi; Sharma Keshav; Imrana Khalid; Osama Khalid; Antoine Khalil; Coralie Khan; Irfan Khan; Quratul Ain Khan; Sushil Khanal; Abid Khatak; Amin Khawaja; Krish Kherajani; Michelle E Kho; Saye Khoo; Ryan Khoo; Denisa Khoo; Nasir Khoso; Khor How Kiat; Yuri Kida; Peter Kiiza; Beathe Kiland Granerud; Anders Benjamin Kildal; Jae Burm Kim; Antoine Kimmoun; Detlef Kindgen-Milles; Alexander King; Nobuya Kitamura; Paul Klenerman; Rob Klont; Gry Kloumann Bekken; Stephen R Knight; Robin Kobbe; Chamira Kodippily; Malte Kohns Vasconcelos; Sabin Koirala; Mamoru Komatsu; ISARIC Collaborator Korten; Caroline Kosgei; Arsène Kpangon; Karolina Krawczyk; Vinothini Krishnan; Sudhir Krishnan; Oksana Kruglova; Deepali Kumar; Ganesh Kumar; Mukesh Kumar; Dinesh Kuriakose; Ethan Kurtzman; Demetrios Kutsogiannis; Galyna Kutsyna; Konstantinos Kyriakoulis; Marie Lachatre; Marie Lacoste; John G. Laffey; Marie Lagrange; Fabrice Laine; Olivier Lairez; Sanjay Lakhey; Antonio Lalueza; Marc Lambert; François Lamontagne; Marie Langelot-Richard; Vincent Langlois; Eka Yudha Lantang; Marina Lanza; Cédric Laouénan; Samira Laribi; Delphine Lariviere; Stéphane Lasry; Sakshi Lath; Naveed Latif; Odile Launay; Didier Laureillard; Yoan Lavie-Badie; Andy Law; Teresa Lawrence; Cassie Lawrence; Minh Le; Clément Le Bihan; Cyril Le Bris; Georges Le Falher; Lucie Le Fevre; Quentin Le Hingrat; Marion Le Maréchal; Soizic Le Mestre; Gwenaël Le Moal; Vincent Le Moing; Hervé Le Nagard; Paul Le Turnier; Ema Leal; Marta Leal Santos; Todd C. Lee; James Lee; Jennifer Lee; Heng Gee Lee; Biing Horng Lee; Yi Lin Lee; Su Hwan Lee; Gary Leeming; Laurent Lefebvre; Bénédicte Lefebvre; Benjamin Lefevre; Sylvie LeGac; Jean-Daniel Lelievre; François Lellouche; Adrien Lemaignen; Véronique Lemee; Anthony Lemeur; Gretchen Lemmink; Ha Sha Lene; Jenny Lennon; Rafael León; Marc Leone; Michela Leone; Quentin Lepiller; François-Xavier Lescure; Olivier Lesens; Mathieu Lesouhaitier; Amy Lester-Grant; Bruno Levy; Yves Levy; Claire Levy-Marchal; Katarzyna Lewandowska; Erwan L'Her; Gianluigi Li Bassi; Janet Liang; Ali Liaquat; Geoffrey Liegeon; Wei Shen Lim; Kah Chuan Lim; Chantre Lima; Lim Lina; Bruno Lina; Andreas Lind; Maja Katherine Lingad; Guillaume Lingas; Sylvie Lion-Daolio; Keibun Liu; Marine Livrozet; Patricia Lizotte; Antonio Loforte; Navy Lolong; Leong Chee Loon; Diogo Lopes; Dalia Lopez-Colon; Jose W. Lopez-Revilla; Anthony L. Loschner; Paul Loubet; Bouchra Loufti; Guillame Louis; Silvia Lourenco; Lara Lovelace-Macon; Lee Lee Low; Marije Lowik; Jia Shyi Loy; Jean Christophe Lucet; Carlos Lumbreras Bermejo; Carlos M Luna; Olguta Lungu; Liem Luong; Nestor Luque; Dominique Luton; Nilar Lwin; Ruth Lyons; Olavi Maasikas; Oryane Mabiala; Moïse Machado; Gabriel Macheda; Hashmi Madiha; Guillermo Maestro de la Calle; Rafael Mahieu; Sophie Mahy; Ana Raquel Maia; Lars S. Maier; Mylène Maillet; Thomas Maitre; Maximilian Malfertheiner; Nadia Malik; Paddy Mallon; Fernando Maltez; Denis Malvy; Victoria Manda; Laurent Mandelbrot; Frank Manetta; Julie Mankikian; Edmund Manning; Aldric Manuel; Ceila Maria Sant′Ana Malaque; Flávio Marino; Daniel Marino; Samuel Markowicz; Charbel Maroun Eid; Ana Marques; Catherine Marquis; Brian Marsh; Laura Marsh; Megan Marshal; John Marshall; Celina Turchi Martelli; Dori-Ann Martin; Emily Martin; Guillaume Martin-Blondel; Martin Martinot; Alejandro Martin-Quiros; João Martins; Ana Martins; Nuno Martins; Caroline Martins Rego; Gennaro Martucci; Olga Martynenko; Eva Miranda Marwali; Marsilla Marzukie; David Maslove; Sabina Mason; Sobia Masood; Basri Mat Nor; Moshe Matan; Meghena Mathew; Daniel Mathieu; Mathieu Mattei; Romans Matulevics; Laurence Maulin; Michael Maxwell; Javier Maynar; Thierry Mazzoni; Natalie Mc Evoy; Lisa Mc Sweeney; Colin McArthur; Colin McArthur; Anne McCarthy; Aine McCarthy; Colin McCloskey; Rachael McConnochie; Sherry McDermott; Sarah E. McDonald; Aine McElroy; Samuel McElwee; Victoria McEneany; Allison McGeer; Chris McKay; Johnny McKeown; Kenneth A. McLean; Paul McNally; Bairbre McNicholas; Elaine McPartlan; Edel Meaney; Cécile Mear-Passard; Maggie Mechlin; Maqsood Meher; Omar Mehkri; Ferruccio Mele; Luis Melo; Kashif Memon; Joao Joao Mendes; Ogechukwu Menkiti; Kusum Menon; Alexander J. Mentzer; Emmanuelle Mercier; Noémie Mercier; Antoine Merckx; Mayka Mergeay-Fabre; Blake Mergler; António Mesquita; Roberta Meta; Osama Metwally; Agnès Meybeck; Dan Meyer; Alison M Meynert; Vanina Meysonnier; Amina Meziane; Mehdi Mezidi; Céline Michelanglei; Isabelle Michelet; Efstathia Mihelis; Vladislav Mihnovit; Hugo Miranda-Maldonado; Nor Arisah Misnan; Tahira Jamal Mohamed; Nik Nur Eliza Mohamed; Asma Moin; David Molina; Elena Molinos; Brenda Molloy; Mary Mone; Agostinho Monteiro; Claudia Montes; Giorgia Montrucchio; Shona C. Moore; Sarah Moore; Lina Morales Cely; Lucia Moro; Catherine Motherway; Ana Motos; Hugo Mouquet; Clara Mouton Perrot; Julien Moyet; Caroline Mudara; Aisha Kalsoom Mufti; Ng Yong Muh; Dzawani Muhamad; Jimmy Mullaert; Fredrik Müller; Karl Erik Müller; Syed Muneeb; Nadeem Munir; Laveena Munshi; Aisling Murphy; Lorna Murphy; Aisling Murphy; Marlène Murris; Srinivas Murthy; Himed Musaab; Himasha Muvindi; Gugapriyaa Muyandy; Dimitra Melia Myrodia; Farah Nadia Mohd-Hanafiah; Dave Nagpal; Alex Nagrebetsky; Mangala Narasimhan; Nageswaran Narayanan; Rashid Nasim Khan; Alasdair Nazerali-Maitland; Nadège Neant; Holger Neb; Erni Nelwan; Raul Neto; Emily Neumann; Pauline Yeung Ng; Wing Yiu Ng; Anthony Nghi; Duc Nguyen; Orna Ni Choileain; Niamh Ni Leathlobhair; Prompak Nitayavardhana; Stephanie Nonas; Nurul Amani Mohd Noordin; Marion Noret; Nurul Faten Izzati Norharizam; Lisa Norman; Alessandra Notari; Mahdad Noursadeghi; Karolina Nowicka; Adam Nowinski; Saad Nseir; Jose I Nunez; Nurnaningsih Nurnaningsih; Dwi Utomo Nusantara; Elsa Nyamankolly; Fionnuala O Brien; Annmarie O Callaghan; Annmarie O'Callaghan; Giovanna Occhipinti; Derbrenn OConnor; Max O'Donnell; Tawnya Ogston; Takayuki Ogura; Tak-Hyuk Oh; Sophie O'Halloran; Katie O'Hearn; Shinichiro Ohshimo; Agnieszka Oldakowska; João Oliveira; Larissa Oliveira; Jee Yan Ong; Wilna Oosthuyzen; Anne Opavsky; Peter Openshaw; Saijad Orakzai; Claudia Milena Orozco-Chamorro; Jamel Ortoleva; Javier Osatnik; Linda O'Shea; Miriam O'Sullivan; Siti Zubaidah Othman; Nadia Ouamara; Rachida Ouissa; Eric Oziol; Maïder Pagadoy; Justine Pages; Mario Palacios; Amanda Palacios; Massimo Palmarini; Giovanna Panarello; Hem Paneru; Lai Hui Pang; Mauro Panigada; Nathalie Pansu; Aurélie Papadopoulos; Rachael Parke; Melissa Parker; Briseida Parra; Taha Pasha; Jérémie Pasquier; Bruno Pastene; Fabian Patauner; Drashti Patel; Mohan Dass Pathmanathan; Luís Patrão; Patricia Patricio; Juliette Patrier; Lisa Patterson; Rajyabardhan Pattnaik; Mical Paul; Christelle Paul; Jorge Paulos; William A. Paxton; Jean-François Payen; Kalaiarasu Peariasamy; Giles J. Peek; Florent Peelman; Nathan Peiffer-Smadja; Vincent Peigne; Mare Pejkovska; Paolo Pelosi; Ithan D. Peltan; Rui Pereira; Daniel Perez; Luis Periel; Thomas Perpoint; Antonio Pesenti; Vincent Pestre; Lenka Petrou; Ventzislava Petrov-Sanchez; Frank Olav Pettersen; Gilles Peytavin; Scott Pharand; Michael Piagnerelli; Walter Picard; Olivier Picone; Maria de Piero; Carola Pierobon; Djura Piersma; Carlos Pimentel; Raquel Pinto; Catarina Pires; Isabelle Pironneau; Lionel Piroth; Ayodhia Pitaloka; Riinu Pius; Laurent Plantier; Hon Shen Png; Julien Poissy; Ryadh Pokeerbux; Maria Pokorska-Spiewak; Sergio Poli; Georgios Pollakis; Diane Ponscarme; Jolanta Popielska; Diego Bastos Porto; Andra-Maris Post; Douwe F. Postma; Pedro Povoa; Diana Póvoas; Jeff Powis; Sofia Prapa; Sébastien Preau; Christian Prebensen; Jean-Charles Preiser; Anton Prinssen; Gamage Dona Dilanthi Priyadarshani; Lucia Proença; Sravya Pudota; Oriane Puéchal; Bambang Pujo Semedi; Mathew Pulicken; Gregory Purcell; Luisa Quesada; Vilmaris Quinones-Cardona; Víctor Quirós González; Else Quist-Paulsen; Mohammed Quraishi; Maia Rabaa; Christian Rabaud; Ebenezer Rabindrarajan; Aldo Rafael; Marie Rafiq; Gabrielle Ragazzo; Mutia Rahardjani; Rozanah Abd Rahman; Ahmad Kashfi Haji Ab Rahman; Arsalan Rahutullah; Fernando Rainieri; Giri Shan Rajahram; Pratheema Ramachandran; Ahmad Afiq Ramli; Blandine Rammaert; Asim Rana; Rajavardhan Rangappa; Ritika Ranjan; Christophe Rapp; Aasiyah Rashan; Thalha Rashan; Ghulam Rasheed; Menaldi Rasmin; Indrek Rätsep; Cornelius Rau; Tharmini Ravi; Ali Raza; Andre Real; Stanislas Rebaudet; Sarah Redl; Brenda Reeve; Attaur Rehman; Liadain Reid; Liadain Reid; Dag Henrik Reikvam; Renato Reis; Jordi Rello; Jonathan Remppis; Martine Remy; Hongru Ren; Hanna Renk; Anne-Sophie Resseguier; Matthieu Revest; Oleksa Rewa; Luis Felipe Reyes; Tiago Reyes; Maria Ines Ribeiro; Antonia Ricchiuto; David Richardson; Denise Richardson; Laurent Richier; Siti Nurul Atikah Ahmad Ridzuan; Jordi Riera; Ana L Rios; Asgar Rishu; Patrick Rispal; Karine Risso; Maria Angelica Rivera Nuñez; Nicholas Rizer; Chiara Robba; André Roberto; Stephanie Roberts; David L. Robertson; Olivier Robineau; Ferran Roche-Campo; Paola Rodari; Simão Rodeia; Bernhard Roessler; Pierre-Marie Roger; Emmanuel Roilides; Juliette Romaru; Roberto Roncon-Albuquerque Jr; Mélanie Roriz; Manuel Rosa-Calatrava; Michael Rose; Dorothea Rosenberger; Nurul Hidayah Mohammad Roslan; Andrea Rossanese; Matteo Rossetti; Bénédicte Rossignol; Patrick Rossignol; Stella Rousset; Carine Roy; Benoît Roze; Desy Rusmawatiningtyas; Clark D. Russell; Maria Ryan; Maeve Ryan; Steffi Ryckaert; Aleksander Rygh Holten; Isabela Saba; Sairah Sadaf; Musharaf Sadat; Valla Sahraei; Maximilien Saint-Gilles; Pranya Sakiyalak; Nawal Salahuddin; Leonardo Salazar; Jodat Saleem; Gabriele Sales; Stéphane Sallaberry; Charlotte Salmon Gandonniere; Hélène Salvator; Olivier Sanchez; Angel Sanchez-Miralles; Vanessa Sancho-Shimizu; Gyan Sandhu; Zulfiqar Sandhu; Pierre-François Sandrine; Marlene Santos; Shirley Sarfo-Mensah; Bruno Sarmento Banheiro; Iam Claire E. Sarmiento; Benjamine Sarton; Ankana Satya; Sree Satyapriya; Rumaisah Satyawati; Egle Saviciute; Parthena Savvidou; Yen Tsen Saw; Justin Schaffer; Tjard Schermer; Arnaud Scherpereel; Marion Schneider; Stephan Schroll; Michael Schwameis; Gary Schwartz; Brendan Scicluna; Janet T. Scott; James Scott-Brown; Nicholas Sedillot; Tamara Seitz; Jaganathan Selvanayagam; Mageswari Selvarajoo; Caroline Semaille; Rasidah Bt Senian; Eric Senneville; Claudia Sepulveda; Filipa Sequeira; Tânia Sequeira; Ary Serpa Neto; Pablo Serrano Balazote; Ellen Shadowitz; Syamin Asyraf Shahidan; Mohammad Shamsah; Anuraj Shankar; Shaikh Sharjeel; Catherine A. Shaw; Victoria Shaw; Ashraf Sheharyar; Rohan Shetty; Dr Rajesh Mohan Shetty; Haixia Shi; Nisreen Shiban; Mohiuddin Shiekh; Nobuaki Shime; Hiroaki Shimizu; Keiki Shimizu; Sally Shrapnel; Pramesh Sundar Shrestha; Shubha Kalyan Shrestha; Hoi Ping Shum; Nassima Si Mohammed; Ng Yong Siang; Jeanne Sibiude; Atif Siddiqui; Piret Sillaots; Catarina Silva; Rogério Silva; Maria Joao Silva; Wai Ching Sin; Dario Sinatti; Punam Singh; Budha Charan Singh; Pompini Agustina Sitompul; Karisha Sivam; Vegard Skogen; Sue Smith; Benjamin Smood; Coilin Smyth; Michelle Smyth; Michelle Smyth; Morgane Snacken; Dominic So; Tze Vee Soh; Joshua Solomon; Tom Solomon; Agnès Sommet; Rima Song; Tae Song; Jack Song Chia; Michael Sonntagbauer; Azlan Mat Soom; Alberto Sotto; Edouard Soum; Marta Sousa; Ana Chora Sousa; Maria Sousa Uva; Vicente Souza-Dantas; Alexandra Sperry; Elisabetta Spinuzza; B. P. Sanka Ruwan Sri Darshana; Shiranee Sriskandan; Sarah Stabler; Thomas Staudinger; Stephanie-Susanne Stecher; Trude Steinsvik; Ymkje Stienstra; Birgitte Stiksrud; Eva Stolz; Amy Stone; Adrian Streinu-Cercel; David Stuart; Ami Stuart; Decy Subekti; Gabriel Suen; Jacky Y. Suen; Asfia Sultana; Charlotte Summers; Dubravka Supic; Deepashankari Suppiah; Magdalena Surovcová; Suwarti Suwarti; Andrey Svistunov; Sarah Syahrin; Konstantinos Syrigos; Jaques Sztajnbok; Konstanty Szuldrzynski; Shirin Tabrizi; Lysa Tagherset; Shahdattul Mawarni Taib; Ewa Talarek; Sara Taleb; Jelmer Talsma; Renaud Tamisier; Maria Lawrensia Tampubolon; Kim Keat Tan; Yan Chyi Tan; Taku Tanaka; Hiroyuki Tanaka; Hayato Taniguchi; Huda Taqdees; Arshad Taqi; Coralie Tardivon; Pierre Tattevin; M Azhari Taufik; Hassan Tawfik; Richard S. Tedder; Tze Yuan Tee; João Teixeira; Sofia Tejada; Marie-Capucine Tellier; Sze Kye Teoh; Vanessa Teotonio; François Téoulé; Pleun Terpstra; Olivier Terrier; Nicolas Terzi; Hubert Tessier-Grenier; Adrian Tey; Alif Adlan Mohd Thabit; Anand Thakur; Zhang Duan Tham; Suvintheran Thangavelu; Vincent Thibault; Simon-Djamel Thiberville; Benoît Thill; Jananee Thirumanickam; Shaun Thompson; Emma C. Thomson; David Thomson; Surain Raaj Thanga Thurai; Ryan S. Thwaites; Paul Tierney; Vadim Tieroshyn; Peter S Timashev; Jean-François Timsit; Noémie Tissot; Jordan Zhien Yang Toh; Maria Toki; Kristian Tonby; Sia Loong Tonnii; Margarida Torres; Antoni Torres; Rosario Maria Torres Santos-Olmo; Hernando Torres-Zevallos; Michael Towers; Tony Trapani; Théo Treoux; Cécile Tromeur; Ioannis Trontzas; Tiffany Trouillon; Jeanne Truong; Christelle Tual; Sarah Tubiana; Helen Tuite; Jean-Marie Turmel; Lance C.W. Turtle; Anders Tveita; Pawel Twardowski; Makoto Uchiyama; PG Ishara Udayanga; Andrew Udy; Roman Ullrich; Alberto Uribe; Asad Usman; Timothy M Uyeki; Cristinava Vajdovics; Piero Valentini; Luís Val-Flores; Amélie Valran; Stijn Van de Velde; Marcel van den Berge; Machteld Van der Feltz; Job van der Palen; Paul van der Valk; Nicky Van Der Vekens; Peter Van der Voort; Sylvie Van Der Werf; Laura van Gulik; Jarne Van Hattem; Carolien van Netten; Frank van Someren Greve; Ilonka van Veen; Hugo Van Willigen; Noémie Vanel; Henk Vanoverschelde; Pooja Varghese; Michael Varrone; Shoban Raj Vasudayan; Charline Vauchy; Shaminee Veeran; Aurélie Veislinger; Sebastian Vencken; Sara Ventura; Annelies Verbon; James Vickers; José Ernesto Vidal; César Vieira; Deepak Vijayan; Joy Ann Villanueva; Judit Villar; Pierre-Marc Villeneuve; Andrea Villoldo; Gayatri Vishwanathan; Benoit Visseaux; Hannah Visser; Chiara Vitiello; Harald Vonkeman; Fanny Vuotto; Suhaila Abdul Wahab; Noor Hidayu Wahab; Nadirah Abdul Wahid; Marina Wainstein; Wan Fadzlina Wan Muhd Shukeri; Chih-Hsien Wang; Steve Webb; Katharina Weil; Tan Pei Wen; Sanne Wesselius; T. Eoin West; Murray Wham; Bryan Whelan; Nicole White; Paul Henri Wicky; Aurélie Wiedemann; Surya Otto Wijaya; Keith Wille; Suzette Willems; Virginie Williams; Calvin Wong; Yew Sing Wong; Teck Fung Wong; Natalie Wright; Gan Ee Xian; Lim Saio Xian; Kuan Pei Xuan; Ioannis Xynogalas; Siti Rohani Binti Mohd Yakop; Masaki Yamazaki; Yazdan Yazdanpanah; Nicholas Yee Liang Hing; Cécile Yelnik; Chian Hui Yeoh; Stephanie Yerkovich; Toshiki Yokoyama; Hodane Yonis; Obada Yousif; Saptadi Yuliarto; Akram Zaaqoq; Marion Zabbe; Kai Zacharowski; Masliza Zahid; Maram Zahran; Nor Zaila Binti Zaidan; Maria Zambon; Miguel Zambrano; Alberto Zanella; Konrad Zawadka; Nurul Zaynah; Hiba Zayyad; Alexander Zoufaly; David Zucman; Mazankowski Heart Institute; The Western Australian COVID-19 Research Response.

## Ethics approval

Ethics Committee approval was given by the WHO Ethics Review Committee (RPC571 and RPC572, 25 April 2013). Institutional approval was additionally obtained by participating sites including the South Central—Oxford C Research Ethics Committee in England (Ref. 13/SC/0149), the Scotland A Research Ethics Committee (Ref. 20/SS/0028) for the UK and the Human Research Ethics Committee (Medical) at the University of the Witwatersrand in South Africa as part of a national surveillance programme (M160667), which collectively represent the majority of the data. Other institutional and national approvals are in place as per local requirements.

## Supplementary Material

dyad012_Supplementary_Data

## Data Availability

The ISARIC-WHO CCP, case report form and consent forms are openly available on the ISARIC website at https://isaric.org/research/covid-19-clinical-research-resources/clinical-characterisation-protocol-ccp/. The statistical analysis plan is openly available on the ISARIC website at https://isaric.org/research/covid-19-clinical-research-resources/accessing-covid-19-clinical-data/approved-uses-of-platform-data/. Most individual patient data are available to researchers approved by the Data Access Committee. The data inventory, application form and terms of access for the COVID-19 Data Platform, hosted by the Infectious Diseases Data Observatory (IDDO), are available at https://www.iddo.org/covid19/data-sharing/accessing-data. All individual participant data are available to individuals from sites who have contributed to the ISARIC COVID-19 Platform via the ISARIC Partner Analysis Scheme. See details via this link: https://isaric.org/research/isaric-partner-analysis-frequently-asked-questions/.
