# Supplementary material for: Characteristics and outcomes of an international cohort of 600 000 hospitalized patients with COVID-19
Source: Int J Epidemiol. 2023 Feb 28;52(2):355–76. doi: 10.1093/ije/dyad012 (PMC10114094; doi:10.1093/ije/dyad012)
Supplement: dyad012_Supplementary_Data [file dyad012_supplementary_data.docx]

Supplementary material

Characteristics and outcomes of an international cohort of 600,000 hospitalised patients with COVID-19

**Figure S1: Numbers of patients by country**


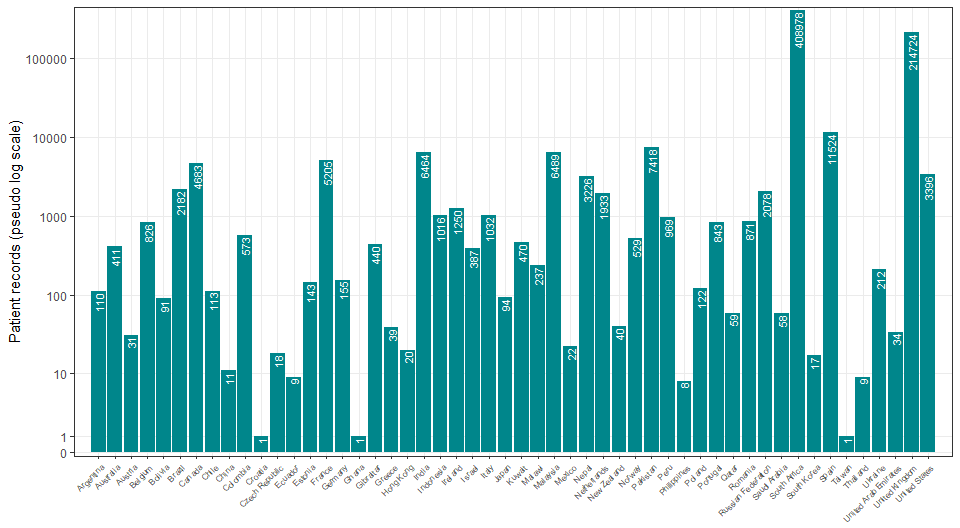


**Table S1: Participants’ characteristics by admission to an intensive care unit (ICU).** Numbers are counts with proportions within columns in parentheses, unless otherwise specified.

| Admission to ICU | Total N |  | Direct admission | Later admission | Never | (Missing) | Total |
| --- | --- | --- | --- | --- | --- | --- | --- |
| Total N (%) |  |  | 34713 (5.0) | 69010 (10.0) | 571030 (82.8) | 14819 (2.1) | 689572 |
| Age | 674753 (100.0) | Median (IQR) | 59.0 (48.0 to 69.0) | 58.0 (47.0 to 69.0) | 57.0 (41.0 to 73.0) | 63.0 (47.0 to 78.0) | 58.0 (42.0 to 72.0) |
|  | 674753 (100.0) | 0-9 | 314 (0.9) | 711 (1.0) | 14102 (2.5) | 360 (2.4) | 15487 (2.2) |
|  |  | 10-19 | 347 (1.0) | 752 (1.1) | 14219 (2.5) | 299 (2.0) | 15617 (2.3) |
|  |  | 20-29 | 1131 (3.3) | 2424 (3.5) | 38604 (6.8) | 758 (5.1) | 42917 (6.2) |
|  |  | 30-39 | 2886 (8.3) | 6137 (8.9) | 66235 (11.6) | 1233 (8.3) | 76491 (11.1) |
|  |  | 40-49 | 4891 (14.1) | 10218 (14.8) | 76166 (13.3) | 1596 (10.8) | 92871 (13.5) |
|  |  | 50-59 | 7960 (22.9) | 16054 (23.3) | 100704 (17.6) | 2244 (15.1) | 126962 (18.4) |
|  |  | 60-69 | 8944 (25.8) | 16006 (23.2) | 91952 (16.1) | 2456 (16.6) | 119358 (17.3) |
|  |  | 70-79 | 6111 (17.6) | 11589 (16.8) | 82672 (14.5) | 2651 (17.9) | 103023 (14.9) |
|  |  | 80-89 | 1871 (5.4) | 4416 (6.4) | 65903 (11.5) | 2444 (16.5) | 74634 (10.8) |
|  |  | 90+ | 258 (0.7) | 703 (1.0) | 20473 (3.6) | 778 (5.3) | 22212 (3.2) |
| Region | 674753 (100.0) | East Asia & Pacific | 775 (2.2) | 804 (1.2) | 6424 (1.1) | 113 (0.8) | 8116 (1.2) |
|  |  | Europe & Central Asia | 14247 (41.0) | 28799 (41.7) | 187216 (32.8) | 11714 (79.0) | 241976 (35.1) |
|  |  | Latin America & Caribbean | 1631 (4.7) | 673 (1.0) | 1588 (0.3) | 177 (1.2) | 4069 (0.6) |
|  |  | Middle East & North Africa | 222 (0.6) | 291 (0.4) | 364 (0.1) | 131 (0.9) | 1008 (0.1) |
|  |  | North America | 1983 (5.7) | 2154 (3.1) | 3486 (0.6) | 456 (3.1) | 8079 (1.2) |
|  |  | South Asia | 12368 (35.6) | 1338 (1.9) | 3122 (0.5) | 280 (1.9) | 17108 (2.5) |
|  |  | Sub-Saharan Africa | 3487 (10.0) | 34951 (50.6) | 368830 (64.6) | 1948 (13.1) | 409216 (59.3) |
| Onset to admission (days) | 610602 (90.5) | Median (IQR) | 5.0 (2.0 to 9.0) | 0.0 (0.0 to 6.0) | 0.0 (0.0 to 4.0) | 2.0 (0.0 to 7.0) | 0.0 (0.0 to 4.0) |
| Length of hospital stay (days) | 674753 (100.0) | Median (IQR) | 7.0 (2.0 to 15.0) | 10.0 (5.0 to 18.0) | 6.0 (2.0 to 11.0) | 2.0 (0.0 to 13.0) | 6.0 (2.0 to 12.0) |
| Body mass index (BMI) (kg/m²) | 18000 (2.7) | Median (IQR) | 27.3 (24.2 to 31.1) | 27.9 (24.5 to 32.4) | 27.3 (23.4 to 31.1) | 28.0 (25.1 to 32.6) | 27.5 (24.0 to 31.2) |
| Heart rate on admission (bpm) | 222187 (32.9) | Median (IQR) | 92.0 (82.0 to 106.0) | 92.0 (82.0 to 105.0) | 89.0 (78.0 to 102.0) | 90.0 (78.0 to 103.0) | 90.0 (79.0 to 103.0) |
| Systolic blood pressure on admission (mmHg) | 228944 (33.9) | Median (IQR) | 130.0 (115.0 to 141.0) | 127.0 (115.0 to 141.0) | 129.0 (115.0 to 144.0) | 128.0 (115.0 to 144.0) | 129.0 (115.0 to 144.0) |
| Diastolic blood pressure on admission (mmHg) | 228963 (33.9) | Median (IQR) | 74.0 (66.0 to 82.0) | 74.0 (67.0 to 82.0) | 75.0 (66.0 to 83.0) | 74.0 (65.0 to 82.0) | 74.0 (66.0 to 83.0) |
| Temperature on admission (degrees C) | 230898 (34.2) | Median (IQR) | 37.0 (36.7 to 37.6) | 37.1 (36.5 to 38.0) | 37.0 (36.5 to 37.8) | 37.0 (36.5 to 37.8) | 37.0 (36.5 to 37.8) |
| Oxygen saturation on admission (%) | 232777 (34.5) | Median (IQR) | 93.0 (89.0 to 96.0) | 94.0 (91.0 to 96.0) | 96.0 (94.0 to 98.0) | 96.0 (93.0 to 97.0) | 96.0 (93.0 to 97.0) |
| - On oxygen therapy | 76517 (11.3) | Median (IQR) | 93.0 (89.0 to 96.0) | 95.0 (92.0 to 96.0) | 95.0 (93.0 to 97.0) | 95.0 (92.0 to 97.0) | 95.0 (92.0 to 97.0) |
| - In room air | 146041 (21.6) | Median (IQR) | 93.9 (88.0 to 97.0) | 94.0 (89.0 to 96.0) | 96.0 (94.0 to 98.0) | 96.0 (94.0 to 98.0) | 96.0 (93.0 to 98.0) |
| - Unknown oxygenation status | 10377 (1.5) | Median (IQR) | 94.0 (91.0 to 96.0) | 95.0 (91.5 to 97.0) | 96.0 (93.0 to 98.0) | 96.0 (93.0 to 98.0) | 96.0 (93.0 to 98.0) |
| Respiratory rate on admission | 222259 (32.9) | Median (IQR) | 24.0 (20.0 to 30.0) | 22.0 (20.0 to 28.0) | 20.0 (18.0 to 24.0) | 20.0 (18.0 to 24.0) | 20.0 (18.0 to 25.0) |
| Outcome | 674753 (100.0) | Censored | 9291 (26.8) | 6300 (9.1) | 39747 (7.0) | 6557 (44.2) | 61895 (9.0) |
|  |  | Death | 11688 (33.7) | 20829 (30.2) | 115508 (20.2) | 2442 (16.5) | 150467 (21.8) |
|  |  | Discharge | 13734 (39.6) | 41881 (60.7) | 415775 (72.8) | 5820 (39.3) | 477210 (69.2) |
| SARS-CoV-2 status | 674753 (100.0) | Positive | 34066 (98.1) | 64649 (93.7) | 515134 (90.2) | 14303 (96.5) | 628152 (91.1) |
|  |  | Unknown | 647 (1.9) | 4361 (6.3) | 55896 (9.8) | 516 (3.5) | 61420 (8.9) |

**Table S2: Participants’ characteristics by age.** Numbers are counts with proportions within columns in parentheses, unless otherwise specified.

| Age | Total N |  | [0,10) | [10,20) | [20,50) | [50,80) | [80,120] | Total |
| --- | --- | --- | --- | --- | --- | --- | --- | --- |
| Total N (%) |  |  | 15487 (2.2) | 15617 (2.3) | 212279 (30.8) | 349343 (50.7) | 96846 (14.0) | 689572 |
| Body mass index (BMI) (kg/m²) | 18582 (2.7) | Median (IQR) | 15.1 (13.1 to 16.6) | 22.9 (18.9 to 28.1) | 27.8 (24.3 to 32.2) | 27.7 (24.3 to 31.3) | 25.6 (22.1 to 29.2) | 27.5 (24.0 to 31.2) |
| Height (cm) | 22677 (3.3) | Median (IQR) | 85.5 (61.5 to 110.0) | 162.0 (155.0 to 170.0) | 168.0 (162.0 to 175.0) | 168.0 (160.0 to 175.0) | 165.0 (158.0 to 170.0) | 167.6 (160.0 to 174.0) |
| Heart rate on admission (bpm) | 229125 (33.2) | Median (IQR) | 140.0 (118.0 to 158.0) | 100.0 (86.0 to 113.0) | 96.0 (85.0 to 109.0) | 90.0 (79.0 to 102.0) | 84.0 (73.0 to 97.0) | 90.0 (79.0 to 103.0) |
| Systolic blood pressure on admission (mmHg) | 236114 (34.2) | Median (IQR) | 100.0 (87.0 to 111.0) | 118.0 (109.0 to 128.0) | 124.0 (114.0 to 136.0) | 130.0 (116.0 to 144.0) | 132.0 (117.0 to 149.0) | 129.0 (115.0 to 144.0) |
| Diastolic blood pressure on admission (mmHg) | 236097 (34.2) | Median (IQR) | 60.0 (48.0 to 70.0) | 71.0 (63.0 to 79.0) | 77.0 (70.0 to 85.0) | 75.0 (67.0 to 83.0) | 72.0 (64.0 to 82.0) | 74.0 (66.0 to 83.0) |
| Temperature on admission (degrees C) | 238084 (34.5) | Median (IQR) | 37.1 (36.6 to 37.9) | 36.9 (36.5 to 37.4) | 37.0 (36.6 to 37.9) | 37.0 (36.6 to 37.9) | 36.9 (36.4 to 37.6) | 37.0 (36.5 to 37.8) |
| Oxygen saturation on admission (%) | 240068 (34.8) | Median (IQR) | 98.0 (97.0 to 100.0) | 98.0 (97.0 to 99.0) | 96.0 (94.0 to 98.0) | 95.0 (92.0 to 97.0) | 96.0 (93.0 to 97.0) | 96.0 (93.0 to 97.0) |
| - On oxygen therapy | 78961 (11.5) | Median (IQR) | 97.0 (94.0 to 99.0) | 96.0 (94.0 to 98.0) | 95.0 (93.0 to 97.0) | 95.0 (92.0 to 97.0) | 95.0 (92.0 to 97.0) | 95.0 (92.0 to 97.0) |
| - In room air | 150585 (21.8) | Median (IQR) | 98.0 (97.0 to 100.0) | 98.0 (97.0 to 99.0) | 97.0 (95.0 to 98.0) | 95.0 (92.0 to 97.0) | 96.0 (94.0 to 97.0) | 96.0 (93.0 to 98.0) |
| - Unknown oxygenation status | 10689 (1.6) | Median (IQR) | 98.0 (97.0 to 100.0) | 98.0 (96.0 to 99.0) | 96.0 (94.0 to 98.0) | 95.0 (92.0 to 97.0) | 96.0 (93.0 to 97.0) | 96.0 (93.0 to 98.0) |
| Respiratory rate on admission | 229421 (33.3) | Median (IQR) | 35.0 (26.0 to 43.0) | 20.0 (18.0 to 22.0) | 20.0 (18.0 to 24.0) | 21.0 (18.0 to 26.0) | 20.0 (18.0 to 24.0) | 20.0 (18.0 to 25.0) |
| Admission to ICU | 674753 (97.9) | Never | 14102 (93.2) | 14219 (92.8) | 181005 (86.7) | 275328 (80.5) | 86376 (92.3) | 571030 (84.6) |
|  |  | Later admission | 711 (4.7) | 752 (4.9) | 18779 (9.0) | 43649 (12.8) | 5119 (5.5) | 69010 (10.2) |
|  |  | Direct admission | 314 (2.1) | 347 (2.3) | 8908 (4.3) | 23015 (6.7) | 2129 (2.3) | 34713 (5.1) |
| Ever received invasive mechanical ventilation | 678815 (98.4) | No | 14801 (96.6) | 14934 (96.7) | 193379 (92.4) | 299102 (87.0) | 91857 (96.9) | 614073 (90.5) |
|  |  | Yes | 525 (3.4) | 511 (3.3) | 15894 (7.6) | 44884 (13.0) | 2928 (3.1) | 64742 (9.5) |
| Ever received non-invasive ventilation | 448973 (65.1) | No | 8317 (97.9) | 10462 (98.1) | 114482 (92.8) | 194394 (86.1) | 75152 (93.3) | 402807 (89.7) |
|  |  | Yes | 178 (2.1) | 204 (1.9) | 8868 (7.2) | 31491 (13.9) | 5425 (6.7) | 46166 (10.3) |
| High flow nasal cannula | 654198 (94.9) | No | 14232 (94.6) | 13993 (95.3) | 169043 (84.1) | 254054 (76.5) | 74402 (81.4) | 525724 (80.4) |
|  |  | Yes | 806 (5.4) | 684 (4.7) | 31892 (15.9) | 78077 (23.5) | 17015 (18.6) | 128474 (19.6) |
| Oxygen therapy via mask | 58 (0.0) | No | 0 (NaN) | 0 (NaN) | 5 (100.0) | 35 (85.4) | 11 (91.7) | 51 (87.9) |
|  |  | Yes | 0 (NaN) | 0 (NaN) | 0 (0.0) | 6 (14.6) | 1 (8.3) | 7 (12.1) |
| Ever received any oxygen supplementation | 683873 (99.2) | No | 12454 (81.0) | 12625 (81.5) | 124271 (59.0) | 132818 (38.3) | 37242 (39.0) | 319410 (46.7) |
|  |  | Yes | 2919 (19.0) | 2869 (18.5) | 86340 (41.0) | 213973 (61.7) | 58362 (61.0) | 364463 (53.3) |
| Outcome | 689572 (100.0) | Censored | 1034 (6.7) | 1205 (7.7) | 16977 (8.0) | 32054 (9.2) | 10625 (11.0) | 61895 (9.0) |
|  |  | Death | 400 (2.6) | 409 (2.6) | 19350 (9.1) | 92776 (26.6) | 37532 (38.8) | 150467 (21.8) |
|  |  | Discharge | 14053 (90.7) | 14003 (89.7) | 175952 (82.9) | 224513 (64.3) | 48689 (50.3) | 477210 (69.2) |
| SARS-CoV-2 status | 689572 (100.0) | Positive | 12555 (81.1) | 12923 (82.7) | 188282 (88.7) | 321509 (92.0) | 92883 (95.9) | 628152 (91.1) |
|  |  | Unknown | 2932 (18.9) | 2694 (17.3) | 23997 (11.3) | 27834 (8.0) | 3963 (4.1) | 61420 (8.9) |

**Table S3: Symptom prevalence.** Numbers are counts with proportions within columns in parentheses, unless otherwise specified.

| Symptom | N recorded (%) |  | N (%) |
| --- | --- | --- | --- |
| Abdominal pain | 215216 (76.7) | No | 196722 (70.1) |
|  |  | Yes | 18494 (6.6) |
|  |  | (Missing) | 65534 (23.3) |
| Altered consciousness/confusion | 215864 (76.9) | No | 175575 (62.5) |
|  |  | Yes | 40289 (14.4) |
|  |  | (Missing) | 64886 (23.1) |
| Asymptomatic | 137868 (49.1) | No | 128030 (45.6) |
|  |  | Yes | 9838 (3.5) |
|  |  | (Missing) | 142882 (50.9) |
| Bleeding | 214161 (76.3) | No | 210329 (74.9) |
|  |  | Yes | 3832 (1.4) |
|  |  | (Missing) | 66589 (23.7) |
| Chest pain | 217104 (77.3) | No | 188392 (67.1) |
|  |  | Yes | 28712 (10.2) |
|  |  | (Missing) | 63646 (22.7) |
| Conjunctivitis | 205050 (73.0) | No | 204316 (72.8) |
|  |  | Yes | 734 (0.3) |
|  |  | (Missing) | 75700 (27.0) |
| Cough | 233265 (83.1) | No | 97182 (34.6) |
|  |  | Yes | 136083 (48.5) |
|  |  | (Missing) | 47485 (16.9) |
| Diarrhoea | 220241 (78.4) | No | 185408 (66.0) |
|  |  | Yes | 34833 (12.4) |
|  |  | (Missing) | 60509 (21.6) |
| Ear pain | 173134 (61.7) | No | 172507 (61.4) |
|  |  | Yes | 627 (0.2) |
|  |  | (Missing) | 107616 (38.3) |
| Fatigue/malaise | 212771 (75.8) | No | 130906 (46.6) |
|  |  | Yes | 81865 (29.2) |
|  |  | (Missing) | 67979 (24.2) |
| Headache | 204976 (73.0) | No | 182565 (65.0) |
|  |  | Yes | 22411 (8.0) |
|  |  | (Missing) | 75774 (27.0) |
| Fever | 232723 (82.9) | No | 108271 (38.6) |
|  |  | Yes | 124452 (44.3) |
|  |  | (Missing) | 48027 (17.1) |
| Lost/altered sense of smell | 170472 (60.7) | No | 159067 (56.7) |
|  |  | Yes | 11405 (4.1) |
|  |  | (Missing) | 110278 (39.3) |
| Lost/altered sense of taste | 167088 (59.5) | No | 153793 (54.8) |
|  |  | Yes | 13295 (4.7) |
|  |  | (Missing) | 113662 (40.5) |
| Lymphadenopathy | 198293 (70.6) | No | 197358 (70.3) |
|  |  | Yes | 935 (0.3) |
|  |  | (Missing) | 82457 (29.4) |
| Muscle ache/joint pain | 204599 (72.9) | No | 168978 (60.2) |
|  |  | Yes | 35621 (12.7) |
|  |  | (Missing) | 76151 (27.1) |
| Runny nose | 198474 (70.7) | No | 192333 (68.5) |
|  |  | Yes | 6141 (2.2) |
|  |  | (Missing) | 82276 (29.3) |
| Seizures | 209568 (74.6) | No | 207239 (73.8) |
|  |  | Yes | 2329 (0.8) |
|  |  | (Missing) | 71182 (25.4) |
| Severe dehydration | 100244 (35.7) | No | 88350 (31.5) |
|  |  | Yes | 11894 (4.2) |
|  |  | (Missing) | 180506 (64.3) |
| Shortness of breath | 234165 (83.4) | No | 93654 (33.4) |
|  |  | Yes | 140511 (50.0) |
|  |  | (Missing) | 46585 (16.6) |
| Skin rash | 208399 (74.2) | No | 203641 (72.5) |
|  |  | Yes | 4758 (1.7) |
|  |  | (Missing) | 72351 (25.8) |
| Sore throat | 199221 (71.0) | No | 185147 (65.9) |
|  |  | Yes | 14074 (5.0) |
|  |  | (Missing) | 81529 (29.0) |
| Vomiting/nausea | 220002 (78.4) | No | 182863 (65.1) |
|  |  | Yes | 37139 (13.2) |
|  |  | (Missing) | 60748 (21.6) |
| Wheezing | 208295 (74.2) | No | 196062 (69.8) |
|  |  | Yes | 12233 (4.4) |
|  |  | (Missing) | 72455 (25.8) |
| WHO | 137785 (49.1) | Yes | 137785 (49.1) |
|  |  | (Missing) | 142965 (50.9) |
| CDC | 179233 (63.8) | Yes | 179233 (63.8) |
|  |  | (Missing) | 101517 (36.2) |
| PHE | 171594 (61.1) | Yes | 171594 (61.1) |
|  |  | (Missing) | 109156 (38.9) |
| ECDC | 192361 (68.5) | Yes | 192361 (68.5) |
|  |  | (Missing) | 88389 (31.5) |

**Figure S2: Proportions reporting each symptom by age for each country.** Countries with fewer than 500 are grouped into ‘other’.


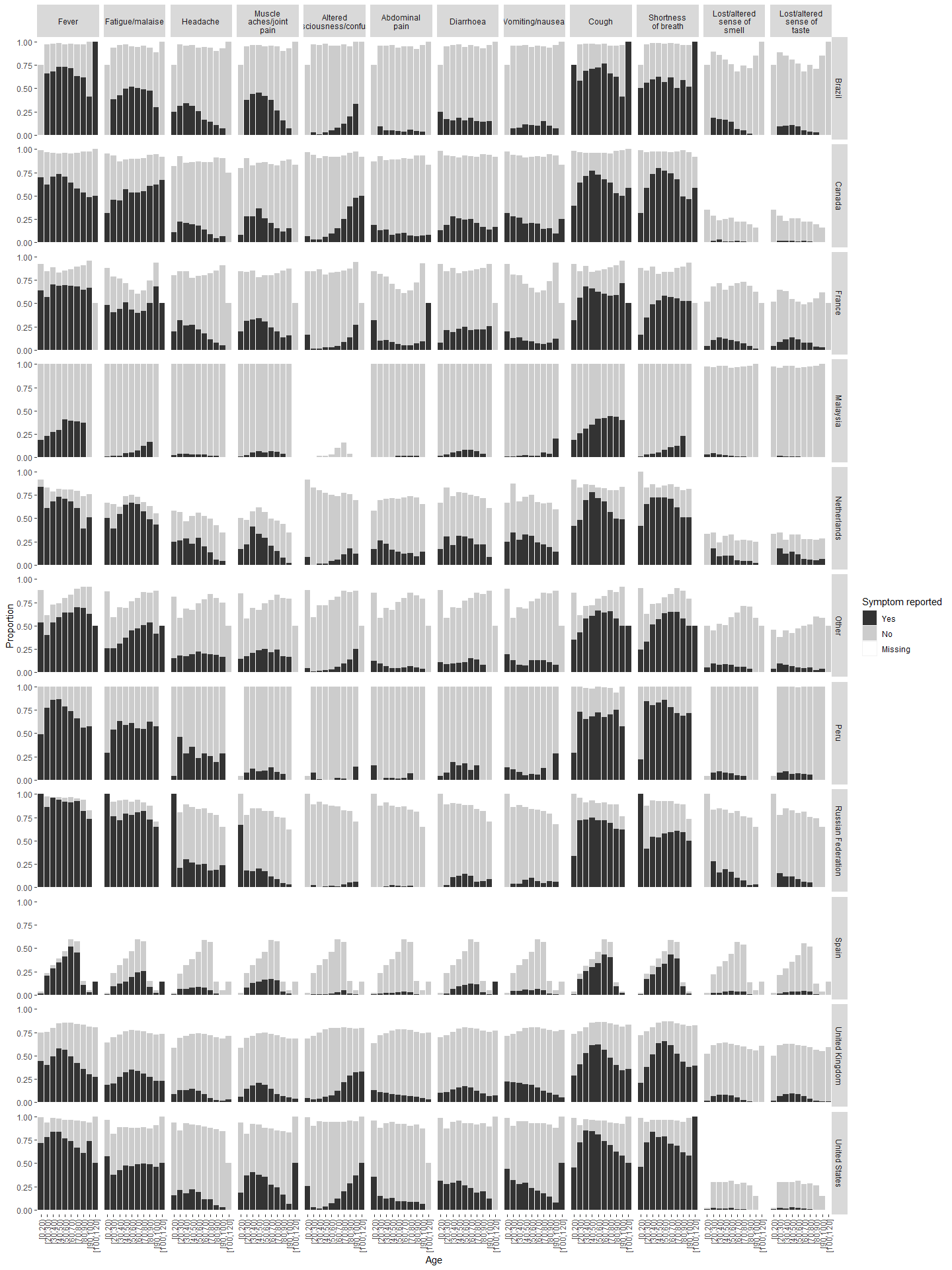


**
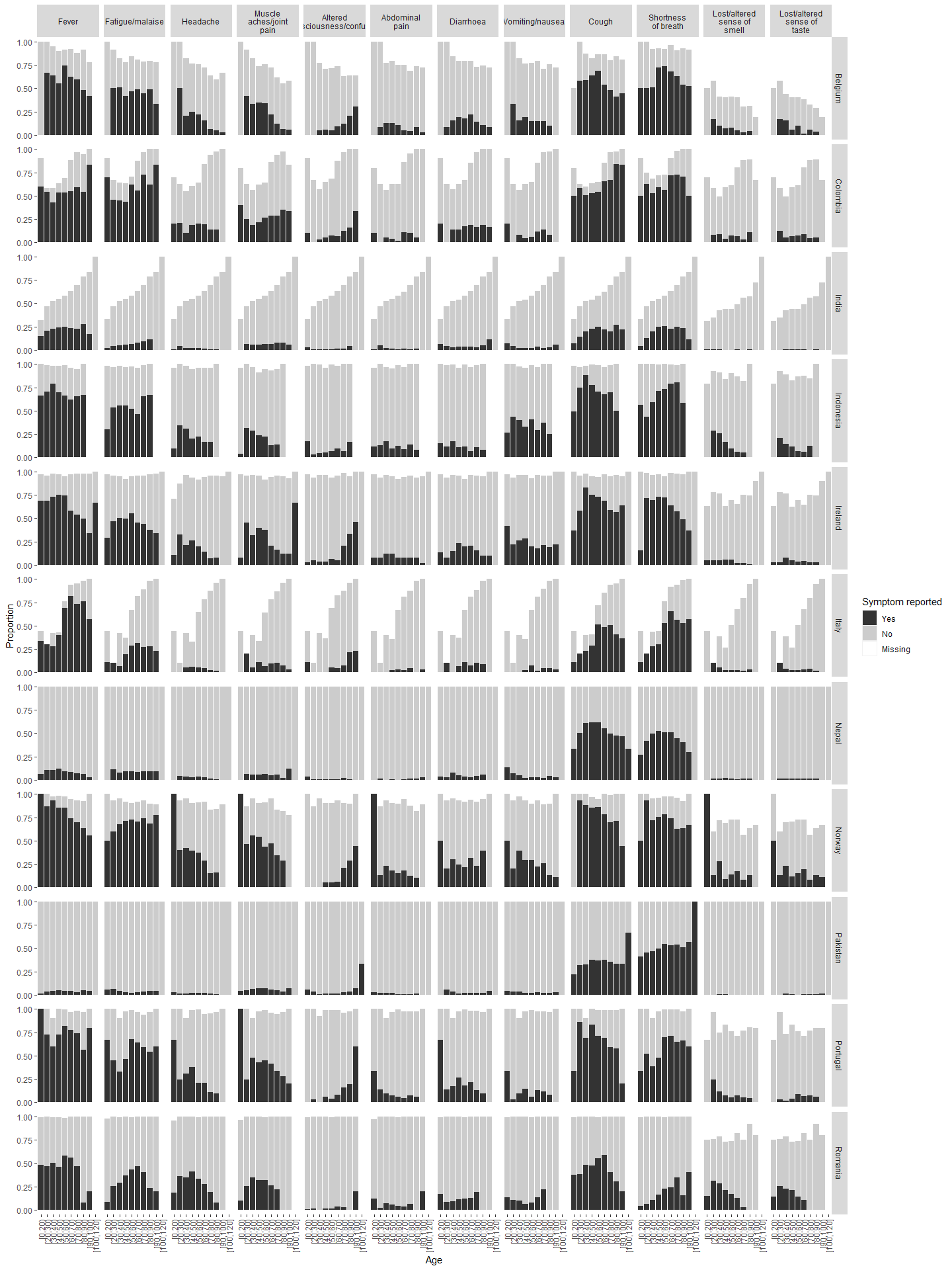
**

**Figure S3: Proportions reporting each symptom by age among individuals with confirmed SARS-CoV-2**


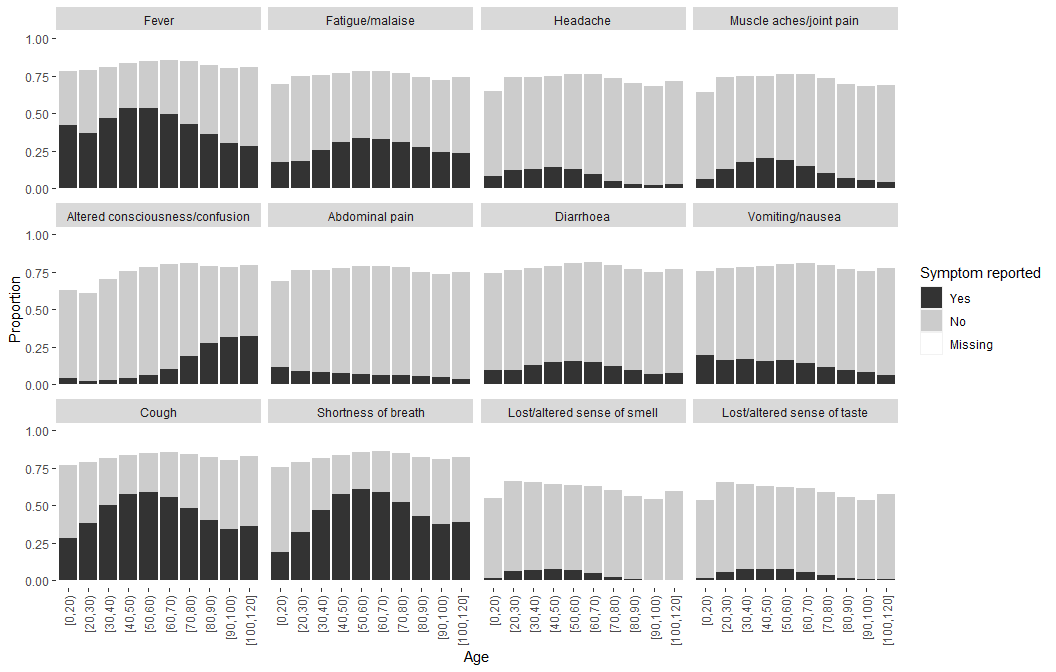


**Figure S4: Proportions reporting each symptom by age among individuals without confirmed SARS-CoV-2**


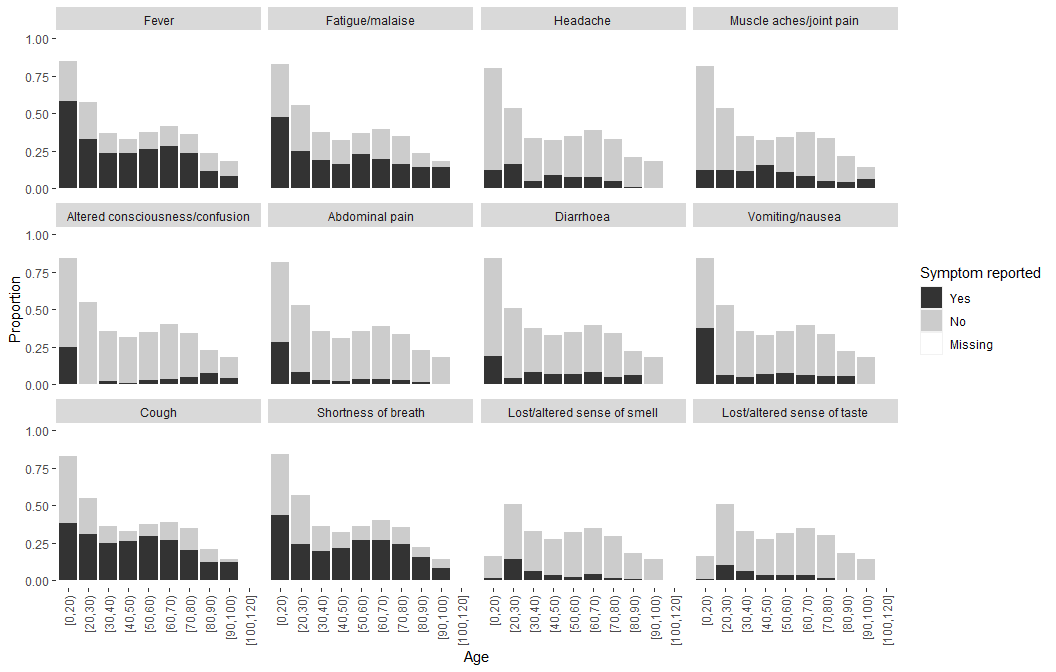


**Figure S5: Proportions with each symptom by SARS-CoV-2 status**


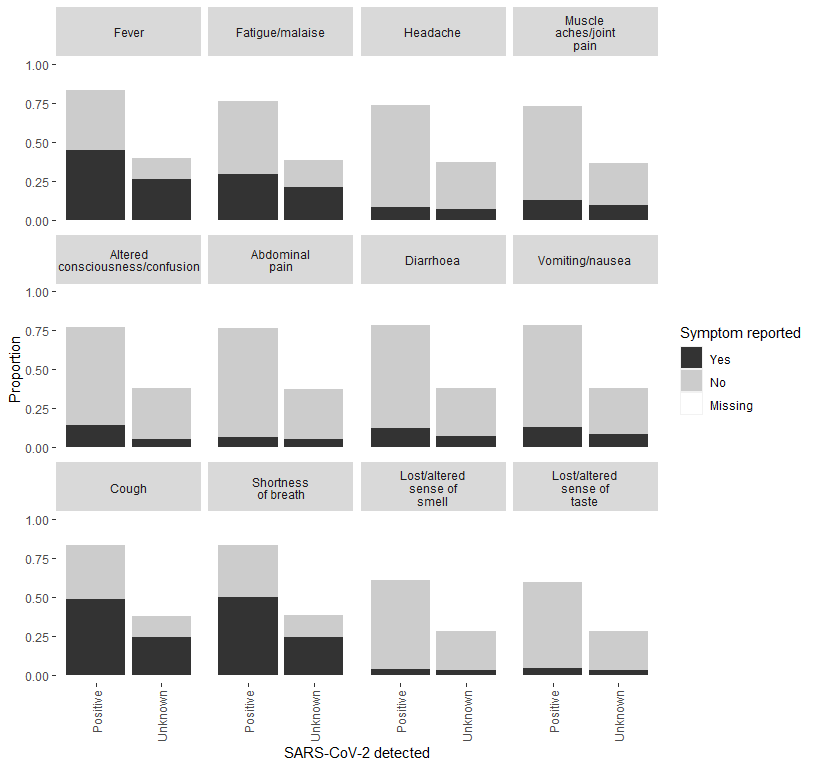


**Figure S6: Proportions meeting each symptom definition by SARS-CoV-2 status**


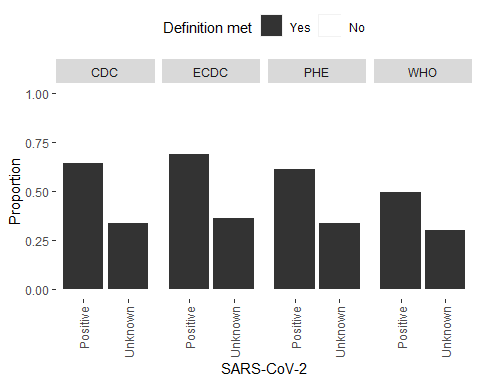


**Figure S7: Proportions meeting each symptom definition by age and SARS-CoV-2 status**


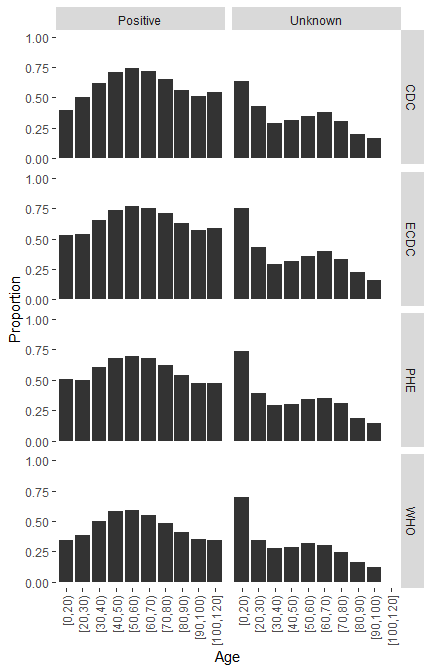


**Table S4: Routine laboratory test results.** Patients with lymphocyte count plus neutrophil count greater than total white blood cell count were excluded.

|  | N | Mean (SD) | Median (IQR) |
| --- | --- | --- | --- |
| **Inflammation** |  |  |  |
| C-Reactive Protein (mg/L) | 116186 (85.3) | 92.5 (75.9) | 74.0 (30.0 to 138.0) |
| White blood cell count ($\times{10}^{9}$ cells per L) | 136210 (100.0) | 8.0 (3.5) | 7.2 (5.4 to 9.8) |
| Neutrophil count ($\times{10}^{9}$ cells per L) | 136210 (100.0) | 6.2 (3.2) | 5.4 (3.8 to 7.9) |
| Lymphocyte count ($\times{10}^{9}$ cells per L) | 136210 (100.0) | 1.1 (0.6) | 0.9 (0.6 to 1.3) |
|  |  |  |  |
| **Liver function tests** |  |  |  |
| Alanine aminotransferase (U/L) | 78871 (57.9) | 35.3 (25.8) | 27.0 (18.0 to 44.0) |
| Aspartate aminotransferase (U/L) | 13910 (10.2) | 46.1 (32.3) | 35.0 (24.0 to 58.0) |
| Total bilirubin ($\mu$mol/L) | 81141 (59.6) | 10.5 (5.8) | 9.0 (6.0 to 13.0) |
|  |  |  |  |
| **Kidney function tests** |  |  |  |
| Urea (mmol/L) | 115521 (84.8) | 8.2 (5.7) | 6.3 (4.5 to 9.7) |

**Figure S8: Routine laboratory test values by age.** Patients with lymphocyte count plus neutrophil count greater than total white blood cell count were excluded.

##
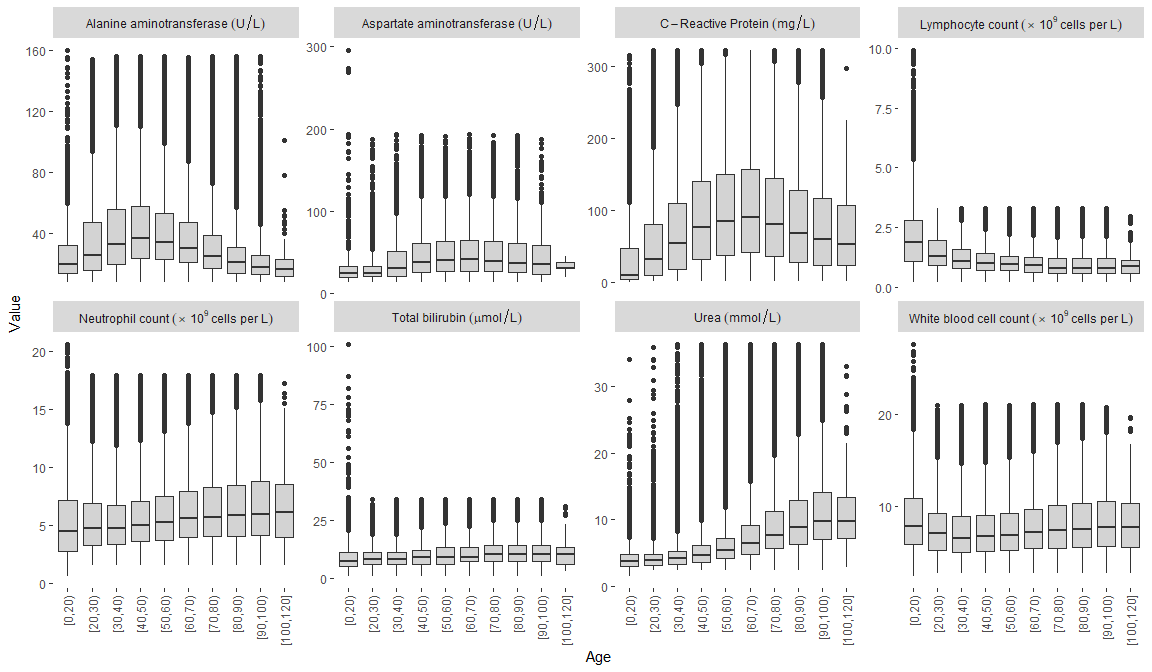


**Table S5: Proportions of individuals with pre-existing comorbidities and risk factors.** Numbers are counts with proportions within columns in parentheses.

| Comorbidity/risk factor | Total N |  | N (%) |
| --- | --- | --- | --- |
| HIV | 516038 (74.8) | No | 489262 (71.0) |
|  |  | Yes | 26776 (3.9) |
|  |  | (Missing) | 173534 (25.2) |
| Asthma | 527448 (76.5) | No | 481010 (69.8) |
|  |  | Yes | 46438 (6.7) |
|  |  | (Missing) | 162124 (23.5) |
| Chronic cardiac disease | 527207 (76.5) | No | 456351 (66.2) |
|  |  | Yes | 70856 (10.3) |
|  |  | (Missing) | 162365 (23.5) |
| Chronic haematological disease | 236353 (34.3) | No | 227259 (33.0) |
|  |  | Yes | 9094 (1.3) |
|  |  | (Missing) | 453219 (65.7) |
| Chronic kidney disease | 520706 (75.5) | No | 479304 (69.5) |
|  |  | Yes | 41402 (6.0) |
|  |  | (Missing) | 168866 (24.5) |
| Chronic neurological disorder | 243583 (35.3) | No | 218373 (31.7) |
|  |  | Yes | 25210 (3.7) |
|  |  | (Missing) | 445989 (64.7) |
| Chronic pulmonary disease | 524841 (76.1) | No | 478651 (69.4) |
|  |  | Yes | 46190 (6.7) |
|  |  | (Missing) | 164731 (23.9) |
| Dementia | 237004 (34.4) | No | 212905 (30.9) |
|  |  | Yes | 24099 (3.5) |
|  |  | (Missing) | 452568 (65.6) |
| Diabetes | 540089 (78.3) | No | 398075 (57.7) |
|  |  | Yes | 142014 (20.6) |
|  |  | (Missing) | 149483 (21.7) |
| Hypertension | 525201 (76.2) | No | 315577 (45.8) |
|  |  | Yes | 209624 (30.4) |
|  |  | (Missing) | 164371 (23.8) |
| Immunosuppression | 122184 (17.7) | No | 117731 (17.1) |
|  |  | Yes | 4453 (0.6) |
|  |  | (Missing) | 567388 (82.3) |
| Liver disease | 252823 (36.7) | No | 244957 (35.5) |
|  |  | Yes | 7866 (1.1) |
|  |  | (Missing) | 436749 (63.3) |
| Malignant neoplasm | 523087 (75.9) | No | 498318 (72.3) |
|  |  | Yes | 24769 (3.6) |
|  |  | (Missing) | 166485 (24.1) |
| Malnutrition | 227297 (33.0) | No | 222552 (32.3) |
|  |  | Yes | 4745 (0.7) |
|  |  | (Missing) | 462275 (67.0) |
| Obesity | 307983 (44.7) | No | 259906 (37.7) |
|  |  | Yes | 48077 (7.0) |
|  |  | (Missing) | 381589 (55.3) |
|  |  |  |  |
|  |  |  |  |
|  |  |  |  |
| Other comorbidities | 629649 (91.3) | No | 523745 (76.0) |
|  |  | Yes | 105904 (15.4) |
|  |  | (Missing) | 59923 (8.7) |
| Rare diseases and inborn errors of metabolism | 121808 (17.7) | No | 121177 (17.6) |
|  |  | Yes | 631 (0.1) |
|  |  | (Missing) | 567764 (82.3) |
| Rheumatologic disorder | 236500 (34.3) | No | 211935 (30.7) |
|  |  | Yes | 24565 (3.6) |
|  |  | (Missing) | 453072 (65.7) |
| Smoking | 202397 (29.4) | No | 137341 (19.9) |
|  |  | Yes | 65056 (9.4) |
|  |  | (Missing) | 487175 (70.6) |
| Transplantation | 122518 (17.8) | No | 120912 (17.5) |
|  |  | Yes | 1606 (0.2) |
|  |  | (Missing) | 567054 (82.2) |
| Tuberculosis | 324692 (47.1) | No | 313308 (45.4) |
|  |  | Yes | 11384 (1.7) |
|  |  | (Missing) | 364880 (52.9) |
| Pregnancy | 268169 (38.9) | No | 249500 (36.2) |
|  |  | Yes | 18669 (2.7) |
|  |  | (Missing) | 421403 (61.1) |

**Figure S9: Prevalence of pre-existing comorbidities and risk factors by age**

**
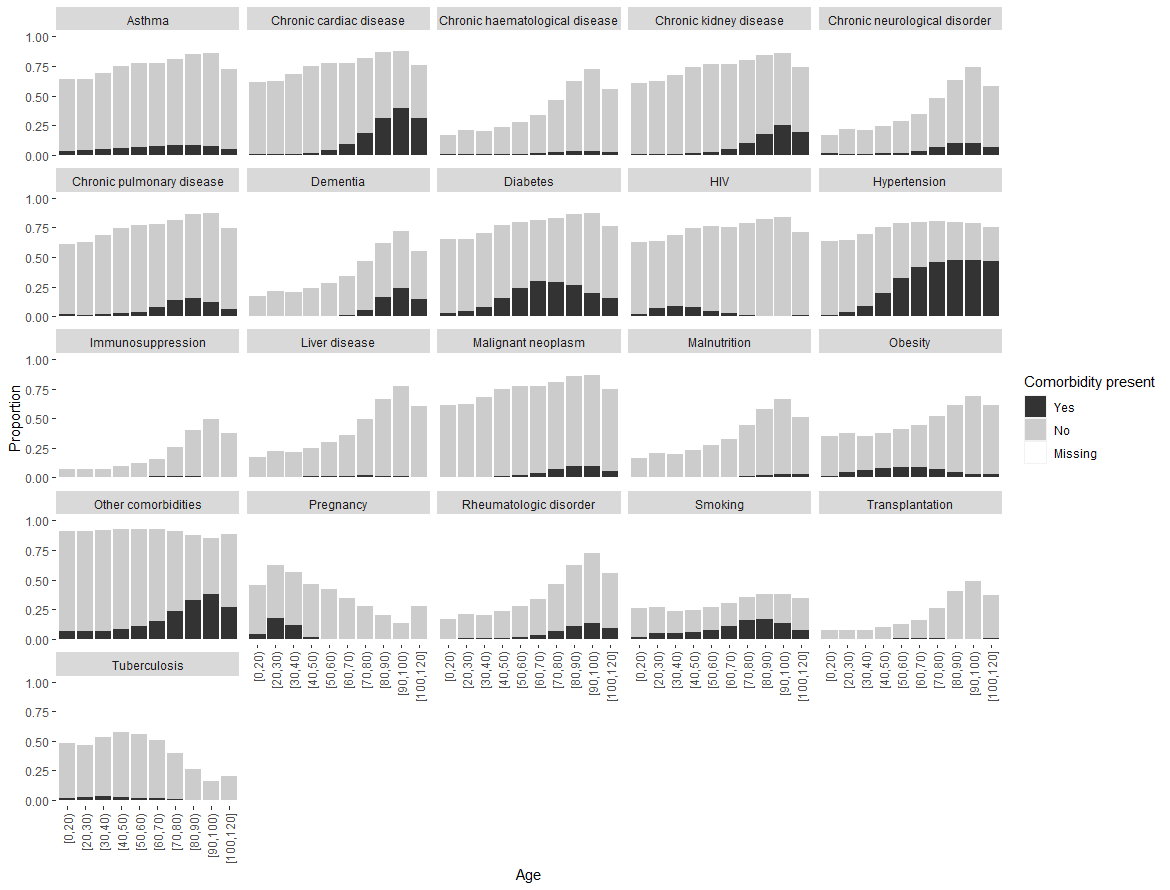
**

**Table S6: Number and proportion of patients receiving each treatment**. Numbers are counts with proportions within columns in parentheses.

| Treatment | Total N |  | N (%) |
| --- | --- | --- | --- |
| Agents acting on the renin angiotensin system | 259355 (92.4) | No | 249684 (88.9) |
|  |  | Yes | 9671 (3.4) |
|  |  | (Missing) | 21395 (7.6) |
| Antibacterial agents | 255031 (90.8) | No | 56736 (20.2) |
|  |  | Yes | 198295 (70.6) |
|  |  | (Missing) | 25719 (9.2) |
| Antifungal agents | 255096 (90.9) | No | 240697 (85.7) |
|  |  | Yes | 14399 (5.1) |
|  |  | (Missing) | 25654 (9.1) |
| Antimalarial agents | 11196 (4.0) | No | 6811 (2.4) |
|  |  | Yes | 4385 (1.6) |
|  |  | (Missing) | 269554 (96.0) |
| Antiviral agents | 251359 (89.5) | No | 199787 (71.2) |
|  |  | Yes | 51572 (18.4) |
|  |  | (Missing) | 29391 (10.5) |
| Convalescent plasma | 170135 (60.6) | No | 167377 (59.6) |
|  |  | Yes | 2758 (1.0) |
|  |  | (Missing) | 110615 (39.4) |
| Corticosteroids | 262385 (93.5) | No | 135994 (48.4) |
|  |  | Yes | 126391 (45.0) |
|  |  | (Missing) | 18365 (6.5) |
| Extracorporeal membrane oxygenation | 265971 (94.7) | No | 264035 (94.0) |
|  |  | Yes | 1936 (0.7) |
|  |  | (Missing) | 14779 (5.3) |
| Immunosuppressants | 14330 (5.1) | No | 7220 (2.6) |
|  |  | Yes | 7110 (2.5) |
|  |  | (Missing) | 266420 (94.9) |
| Inhaled nitric oxide | 239255 (85.2) | No | 238044 (84.8) |
|  |  | Yes | 1211 (0.4) |
|  |  | (Missing) | 41495 (14.8) |
| Inotropes/vasopressors | 257227 (91.6) | No | 233837 (83.3) |
|  |  | Yes | 23390 (8.3) |
|  |  | (Missing) | 23523 (8.4) |
| Interleukin inhibitors | 169937 (60.5) | No | 165310 (58.9) |
|  |  | Yes | 4627 (1.6) |
|  |  | (Missing) | 110813 (39.5) |
| Neuromuscular blocking agents | 58965 (21.0) | No | 49102 (17.5) |
|  |  | Yes | 9863 (3.5) |
|  |  | (Missing) | 221785 (79.0) |
| Off-label/compassionate use medications | 173749 (61.9) | No | 167408 (59.6) |
|  |  | Yes | 6341 (2.3) |
|  |  | (Missing) | 107001 (38.1) |
| Other interventions | 65024 (23.2) | No | 57060 (20.3) |
|  |  | Yes | 7964 (2.8) |
|  |  | (Missing) | 215726 (76.8) |
| Other treatments administered for COVID-19 including experimental or compassionate use | 18353 (6.5) | No | 16360 (5.8) |
|  |  | Yes | 1993 (0.7) |
|  |  | (Missing) | 262397 (93.5) |
| Anticoagulant | 33588 (12.0) | No | 12120 (4.3) |
|  |  | Yes | 21468 (7.6) |
|  |  | (Missing) | 247162 (88.0) |
| Tracheostomy | 250516 (89.2) | No | 243343 (86.7) |
|  |  | Yes | 7173 (2.6) |
|  |  | (Missing) | 30234 (10.8) |

**Table S7: HRs and 95% CIs for death by each comorbidity or risk factor.** The reference group is not having the particular comorbidity/risk factor*.* Models were adjusted for age and age${}^{2}$, stratified by sex and country. n = 689,572

| Comorbidity/risk factor | HR | 95% CI |
| --- | --- | --- |
| AIDS/HIV | 1.77 | (1.72, 1.81) |
| Asthma | 0.99 | (0.97, 1.01) |
| Chronic cardiac disease | 1.26 | (1.24, 1.29) |
| Chronic haematological disease | 1.17 | (1.12, 1.22) |
| Chronic kidney disease | 1.46 | (1.44, 1.49) |
| Chronic neurological disorder | 1.12 | (1.09, 1.15) |
| Chronic pulmonary disease | 1.26 | (1.24, 1.29) |
| Dementia | 1.16 | (1.13, 1.19) |
| Diabetes | 1.35 | (1.34, 1.37) |
| Hypertension | 1.18 | (1.17, 1.20) |
| Immunosuppression | 1.34 | (1.26, 1.43) |
| Liver disease | 1.28 | (1.22, 1.35) |
| Malignant neoplasm | 1.29 | (1.26, 1.32) |
| Malnutrition | 1.18 | (1.11, 1.25) |
| Obesity | 1.24 | (1.21, 1.27) |
| Other comorbidities | 1.17 | (1.15, 1.19) |
| Rare diseases and inborn errors of metabolism | 1.41 | (1.18, 1.69) |
| Rheumatologic disorder | 0.99 | (0.96, 1.02) |
| Smoking | 1.10 | (1.07, 1.12) |
| Transplantation | 1.52 | (1.37, 1.68) |
| Tuberculosis | 1.56 | (1.50, 1.62) |
| Pregnancy | 0.36 | (0.32, 0.40) |

**Table S8: HRs and 95% CIs for death by each symptom.** The reference group is not having the particular symptom*.* Models were adjusted for age and age${}^{2}$, stratified by sex and country. n = 290,750

| Symptom | HR | 95% CI |
| --- | --- | --- |
| Abdominal pain | 0.84 | (0.81, 0.88) |
| Altered consciousness/confusion | 1.32 | (1.29, 1.35) |
| Anorexia | 1.09 | (0.99, 1.20) |
| Asymptomatic | 0.78 | (0.70, 0.86) |
| Bleeding | 1.00 | (0.94, 1.07) |
| Chest pain | 0.84 | (0.81, 0.87) |
| Conjunctivitis | 0.86 | (0.71, 1.04) |
| Cough | 1.16 | (1.13, 1.18) |
| Diarrhoea | 0.93 | (0.91, 0.96) |
| Ear pain | 0.99 | (0.79, 1.24) |
| Fatigue/malaise | 1.06 | (1.04, 1.08) |
| Headache | 0.67 | (0.64, 0.70) |
| Fever | 1.18 | (1.16, 1.20) |
| Inability to walk | 1.84 | (1.67, 2.03) |
| Lost/altered sense of smell | 0.68 | (0.64, 0.72) |
| Lost/altered sense of taste | 0.79 | (0.75, 0.83) |
| Lymphadenopathy | 1.45 | (1.27, 1.65) |
| Muscle aches/joint pain | 0.88 | (0.86, 0.91) |
| Runny nose | 0.98 | (0.91, 1.06) |
| Seizures | 0.96 | (0.87, 1.06) |
| Severe dehydration | 1.36 | (1.31, 1.41) |
| Shortness of breath | 1.76 | (1.72, 1.80) |
| Skin rash | 1.30 | (1.22, 1.37) |
| Sore throat | 0.99 | (0.94, 1.03) |
| Vomiting/nausea | 0.81 | (0.79, 0.83) |
| Wheezing | 1.40 | (1.35, 1.45) |

**Figure S10: Proportion receiving each treatment by age**


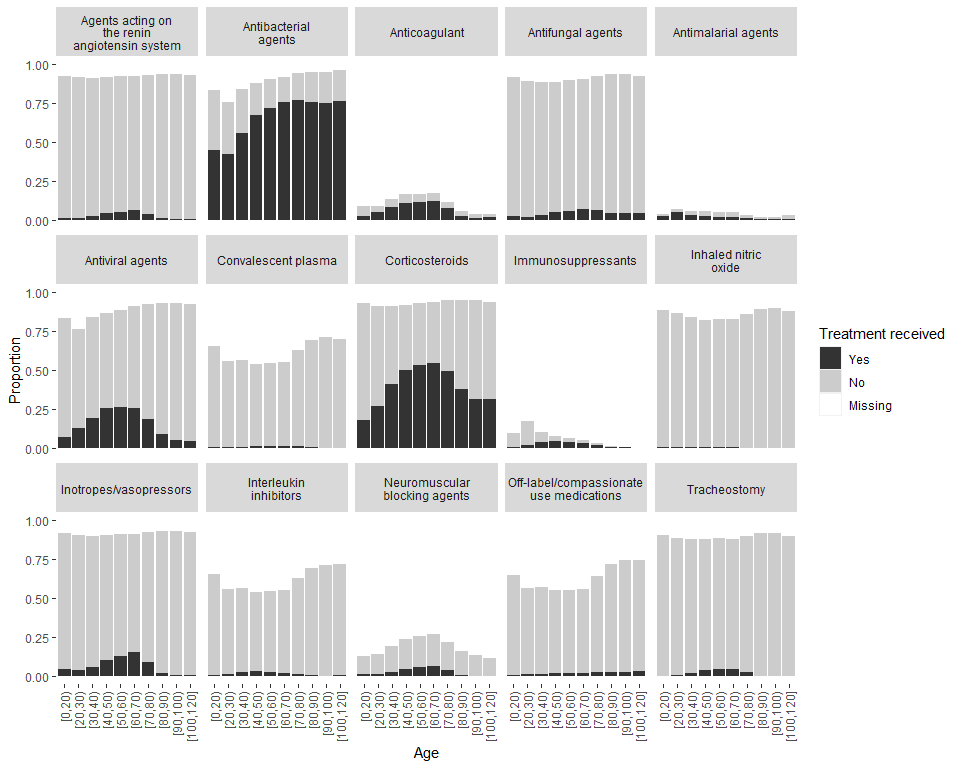


**Figure S11: Use of corticosteroids over time in all patients and in patients who did and did not receive oxygen therapy.** The green line shows the month that results on corticosteroids from the RECOVERY trial were published.

##
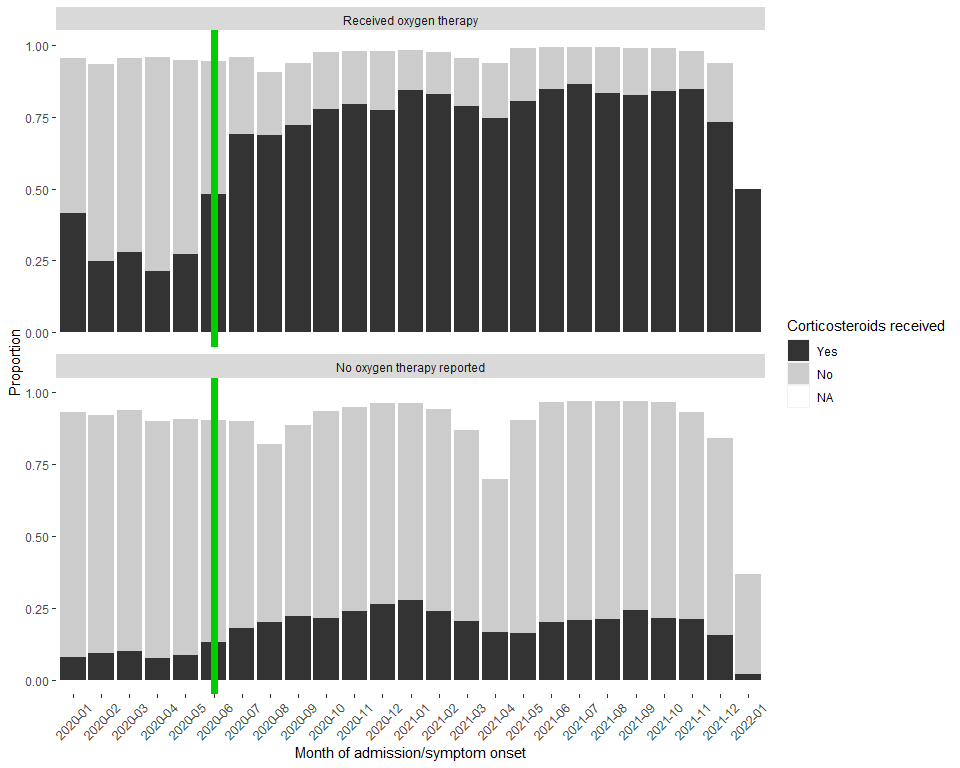


**Figure S12: Case fatality ratio (CFR) by month of admission in each country**

**
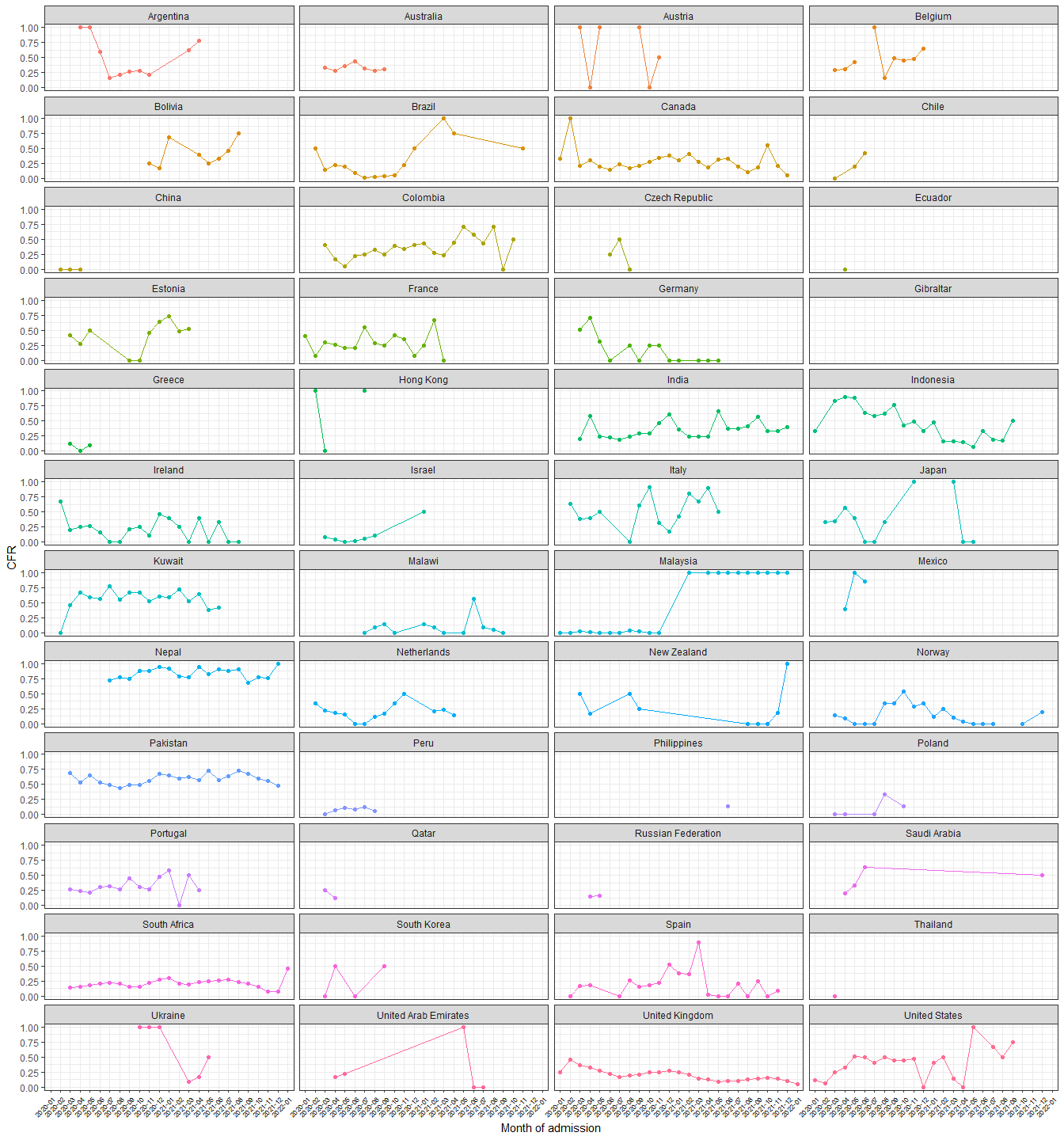
**

**Figure S13: Cumulative incidence curves of death and discharge**

##
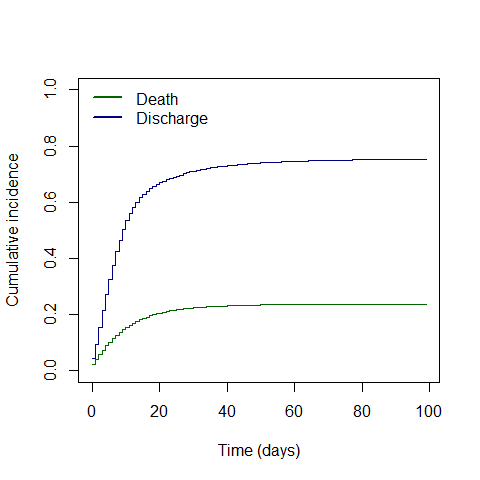


**Figure S14: Hazard ratios (HRs) and 95% confidence intervals (CIs) for death by age group.** Adjusted for sex and stratified by country; HRs are plotted on logarithmic scale.


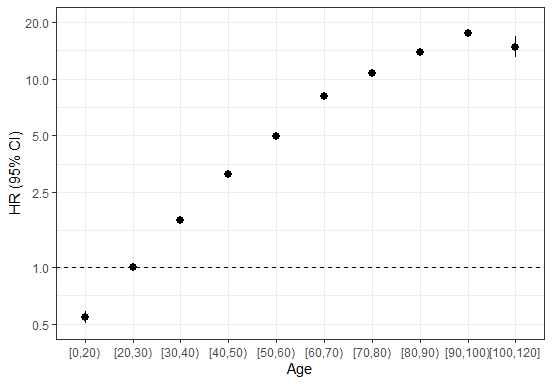


**Figure S15: HRs and 95% CIs for death per 10 years higher age by country.** Model stratified by sex.


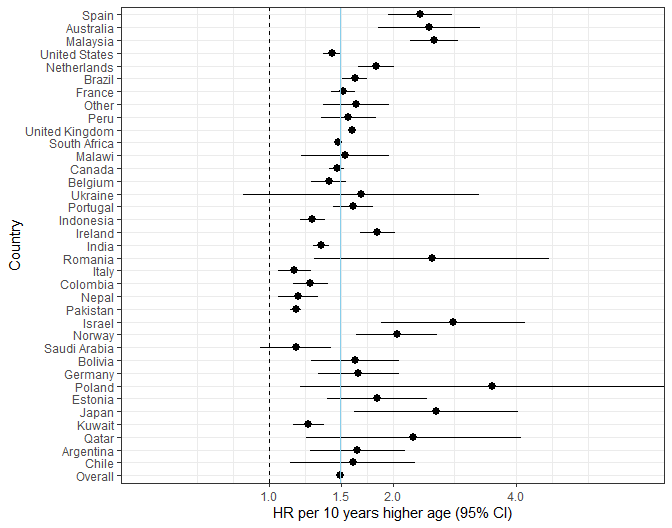


**Figure S16: HRs and 95% CIs for death for males vs females by country.** Model adjusted for age (numeric).


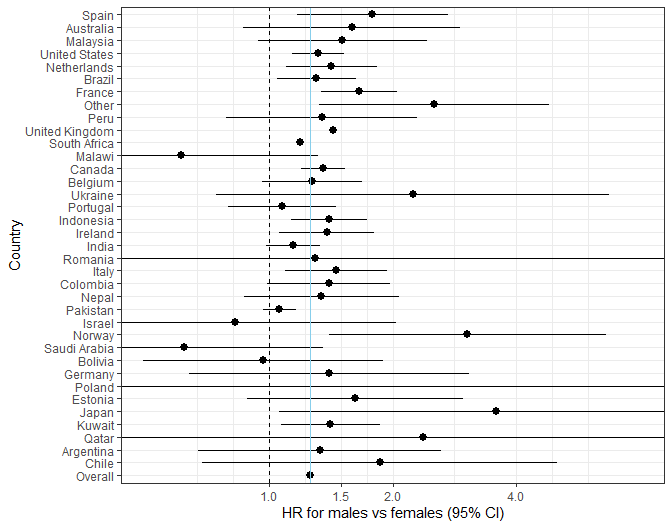


**Figure S17: HRs for death by each comorbidity or risk factor in (A) 2020 and (B) 2021** Adjusted for age and age^2^, stratified by sex and country

*
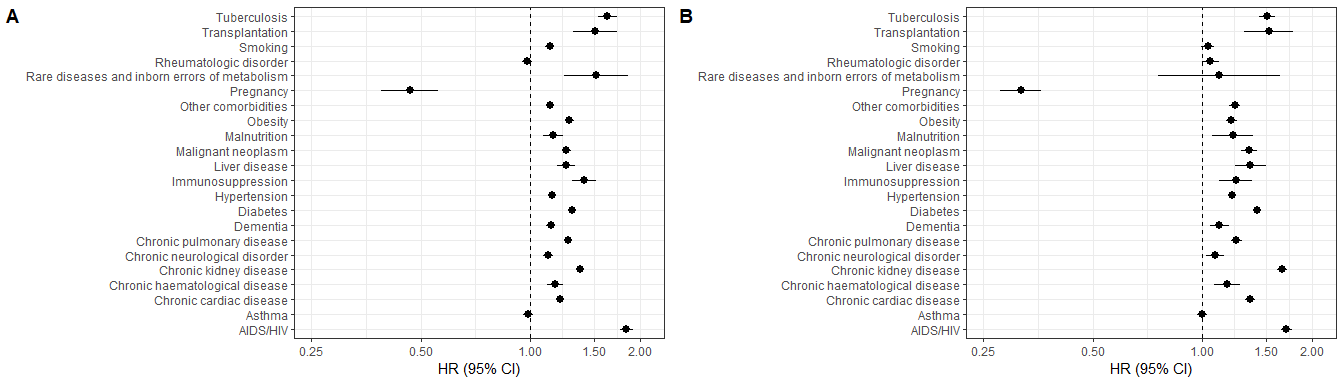
*

**Figure S18: HRs by age of ICU admission in (A) United Kingdom, (B) South Africa, (C) other countries, and of IMV use in (D) United Kingdom, (E) South Africa, (F) other countries.** Adjusted for sex.

**
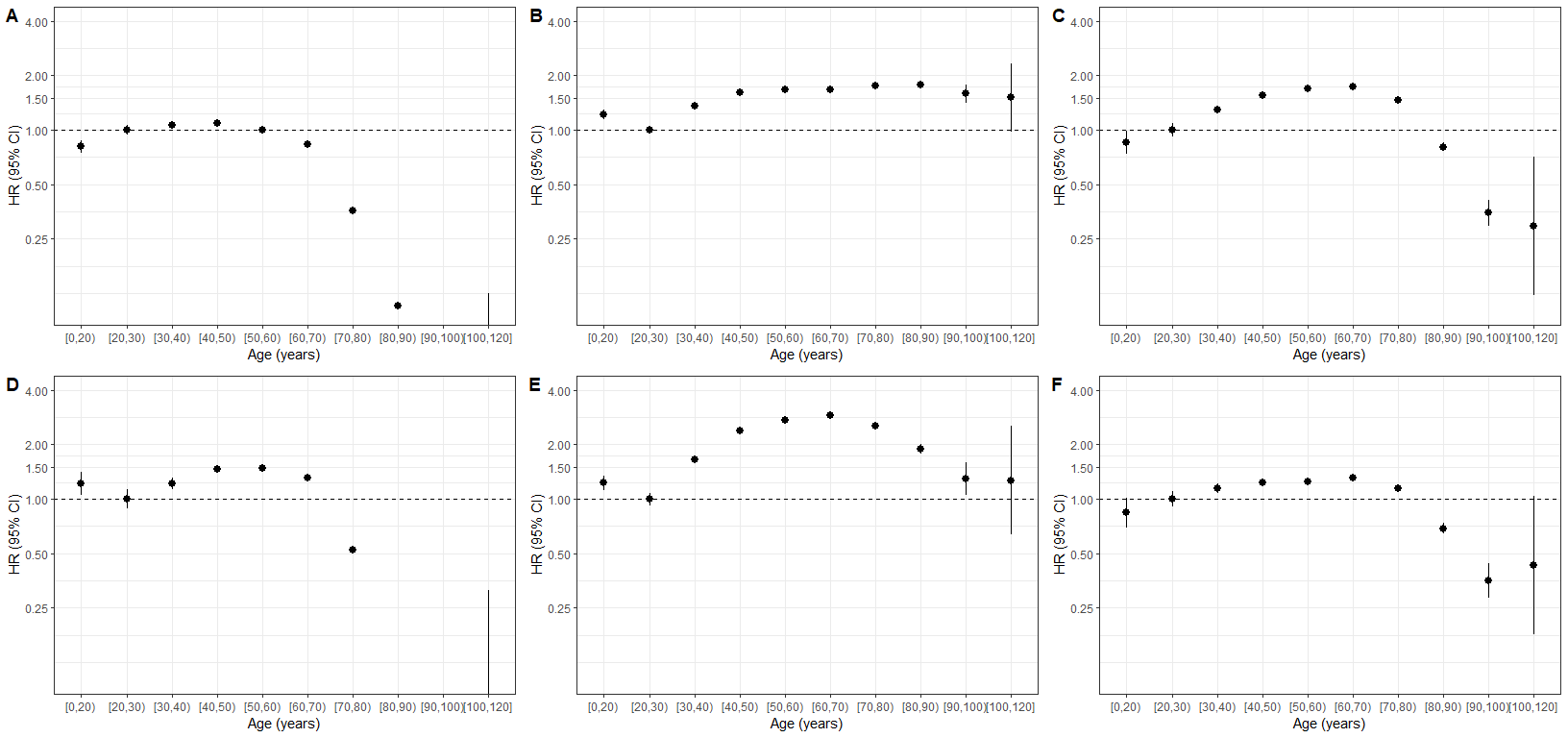
**

## Supplementary methods

**Case Report Forms**

The ISARIC-WHO Case Report Forms (CRFs) were used to collect data for individuals presenting with suspected or confirmed COVID-19. The CRFs are a part of the ISARIC/WHO Clinical Characterisation Protocol, a harmonised protocol for investigation of severe acute infections by pathogens of public health importance. Three versions of the CRF were available for use: the ISARIC WHO Clinical Characterisation Protocol for Severe Emerging Infections UK (CCP-UK) CRF, the COVID‐19 CORE CRF, and the COVID-19 RAPID CRF. The CCP-UK CRF was used to capture data for patients in UK hospitals, while the CORE and RAPID CRFs were used to capture data for patients outside the UK. The CORE CRF is a comprehensive form with variables collected to understand a broad spectrum of disease and management, while the RAPID CRF includes fewer variables than the CORE CRF and was designed for use where data collection resources are limited. CRFs were completed electronically through ISARIC’s Research Electronic Data Capture (REDCap®, version 8.11.11, Vanderbilt University, Nashville, Tenn.), hosted by the University of Oxford. Several regions did not use the ISARIC-WHO CRFs to collect data, but contributed data to the ISARIC data platform through their local CRFs or independent studies. All data submitted to the ISARIC data platform were harmonised to the CDISC SDTM standard (Study Data Tabulation Model) (version 1.7, Clinical Data Interchange Standards Consortium, Austin, Tex.).

For this study, data for 214,723 patients from 214 sites were collected using the CCP-UK CRF, 41,959 patients from 239 sites using the CORE CRF, and 6167 patients from 93 sites using the RAPID CRF. Other patient data were collected using locally-developed CRFs, usually based on the ISARIC CRFs.

**Variables used**

Age was categorized into 10-year groups, with age <20 and $\geq$ 100 into single groups.

Non-invasive ventilation was defined as bilevel positive airway pressure (BIPAP), continuous bilevel positive airway pressure (CPAP) or other unspecified type of non-invasive ventilation.

The following COVID-19 symptom definitions were used:

- World Health Organization (WHO):
  1. A combination of acute fever and cough,
  Or
  2. A combination of three or more of: fever, cough, general weakness and fatigue, headache, myalgia, sore throat, coryza, dyspnoea, anorexia, nausea and vomiting, diarrhoea, altered mental status
- Centers for Disease Control and Prevention (CDC), United States:
  1. At least two of: fever, chills (not available), rigors (not available), myalgia, headache, sore throat, new olfactory and taste disorder,
  Or
  2. At least one of: cough, shortness of breath, difficulty breathing (not available)
- Public Health England
  New cough, or temperature $\geq$ 37.8 ${}^{\circ}$C, or a loss or change in sense of smell or taste
- European Centre for Disease Prevention and Control
  At least one of: cough, fever, shortness of breath, sudden onset anosmia, ageusia or dysgeusia

**References:**

1. CDC. Coronavirus Disease 2019 (COVID-19) 2020 Interim Case Definition. 2021. Available at: https://ndc.services.cdc.gov/case-definitions/coronavirus-disease-2019-2021/.

2. ECDC. Case definition for coronavirus disease 2019 (COVID-19), as of 3 December 2020. 2020. Available at: https://www.ecdc.europa.eu/en/covid-19/surveillance/case-definition.

3. UKHSA. COVID-19: investigation and initial clinical management of possible cases. 2020. Available at: https://www.gov.uk/government/publications/wuhan-novel-coronavirus-initial-investigation-of-possible-cases/investigation-and-initial-clinical-management-of-possible-cases-of-wuhan-novel-coronavirus-wn-cov-infection.

4. WHO. World Health Organization. WHO COVID-19: Case definition. Updated in Public health surveillance for COVID-19. 2020. Available at: https://www.who.int/publications/i/item/WHO-2019-nCoV-Surveillance_Case_Definition-2020.2.

**Author contributions**

**CRediT author statement (based on Brand et al., 2015, doi: 10.1087/20150211)**

**Conceptualization**: CK, PLO, LM, EAD, CAD, AR, MH, MGS, GC, AH, LS, MGP, PH

**Methodology**: CK, PLO, LM, EAD, CAD

**Software and formal analysis**: CK, ME, BWC, EAD, MH, JW

**Data curation**: BWC, LM

**Administration**: BWC, LM

**Writing - original draft**: CK, PLO, JD, JB, AD, LS, LM, AR, MGP, BWC

**Visualization**: CK, EAD, JB

**Writing - review and editing:** All authors.
